# Vitamin and Amino Acid Auxotrophy in Anaerobic Consortia Operating under Methanogenic Conditions

**DOI:** 10.1128/mSystems.00038-17

**Published:** 2017-10-31

**Authors:** Valerie Hubalek, Moritz Buck, BoonFei Tan, Julia Foght, Annelie Wendeberg, David Berry, Stefan Bertilsson, Alexander Eiler

**Affiliations:** a Department of Ecology and Genetics, Limnology and Science for Life Laboratory, Uppsala University, Uppsala, Sweden; b Department of Forest Mycology and Plant Pathology, Swedish University of Agricultural Sciences (SLU), Uppsala, Sweden; c NBIS, National Bioinformatics Infrastructure Sweden, Uppsala, Sweden; d Department of Biological Sciences, University of Alberta, Edmonton, Alberta, Canada; e Center for Environmental Sensing and Modelling, Singapore-MIT Alliance for Research and Technology, Leipzig, Germany; f Centre for Environmental Research, Environmental Microbiology, Leipzig, Germany; g Department of Microbiology and Ecosystem Science, Division of Microbial Ecology, University of Vienna, Vienna, Austria; h eDNA Solutions AB, Mölndal, Sweden; University of Tennessee, Knoxville

**Keywords:** Black Queen hypothesis, metagenomics, petroleum, syntrophy

## Abstract

Syntrophy among *Archaea* and *Bacteria* facilitates the anaerobic degradation of organic compounds to CH_4_ and CO_2_. Particularly during aliphatic and aromatic hydrocarbon mineralization, as in the case of crude oil reservoirs and petroleum-contaminated sediments, metabolic interactions between obligate mutualistic microbial partners are of central importance. Using micromanipulation combined with shotgun metagenomic approaches, we describe the genomes of complex consortia within short-chain alkane-degrading cultures operating under methanogenic conditions. Metabolic reconstruction revealed that only a small fraction of genes in the metagenome-assembled genomes encode the capacity for fermentation of alkanes facilitated by energy conservation linked to H_2_ metabolism. Instead, the presence of inferred lifestyles based on scavenging anabolic products and intermediate fermentation products derived from detrital biomass was a common feature. Additionally, inferred auxotrophy for vitamins and amino acids suggests that the hydrocarbon-degrading microbial assemblages are structured and maintained by multiple interactions beyond the canonical H_2_-producing and syntrophic alkane degrader-methanogen partnership. Compared to previous work, our report points to a higher order of complexity in microbial consortia engaged in anaerobic hydrocarbon transformation.

**IMPORTANCE** Microbial interactions between *Archaea* and *Bacteria* mediate many important chemical transformations in the biosphere from degrading abundant polymers to synthesis of toxic compounds. Two of the most pressing issues in microbial interactions are how consortia are established and how we can modulate these microbial communities to express desirable functions. Here, we propose that public goods (i.e., metabolites of high energy demand in biosynthesis) facilitate energy conservation for life under energy-limited conditions and determine the assembly and function of the consortia. Our report suggests that an understanding of public good dynamics could result in new ways to improve microbial pollutant degradation in anaerobic systems.

## Abstract (precis)

Microbial interactions between *Archaea* and *Bacteria* mediate many important chemical transformations in the biosphere from degrading abundant polymers to synthesis of toxic compounds. Two of the most pressing issues in microbial interactions are how consortia are established and how we can modulate these microbial communities to express desirable functions. Here, we propose that public goods (i.e., metabolites of high energy demand in biosynthesis) facilitate energy conservation for life under energy-limited conditions and determine the assembly and function of the consortia. Our report suggests that an understanding of public good dynamics could result in new ways to improve microbial pollutant degradation in anaerobic systems.

## INTRODUCTION

Microbial consortia that degrade hydrocarbons under anaerobic conditions hold the potential to influence hydrocarbon profiles with either adverse or beneficial effects on the recovery and refining of oil (1–3). In environmental restoration, microbial degradation is seen as an effective and environmentally friendly method of treatment of oil-contaminated sites. Still, in anaerobic systems, bioremediation efforts are challenged by low efficiency and poor success due to the fast depletion of accessible electron acceptors that in turn leads to long turnover times and incomplete removal of the contaminants in the environment (4). Additionally, there is a severe problem with unwanted contamination of adjacent systems with pollutants such as toxic metals, naphthenic acids, and polycyclic aromatic compounds (5–7) as well as accelerated release of the potent greenhouse gas methane from the biodegradation process (8).

Analyses of chemical profiles and metagenomic data from hydrocarbon-degrading consortia maintained in laboratory-scale cultures (9–14) have identified some of the organisms and metabolic pathways involved in the anaerobic degradation of various hydrocarbons, such as alkanes, benzenes, and naphthalenes (13, 15–20). The anaerobic degradation of hydrocarbons under methanogenic conditions is usually a two- to three-step process involving fermentation either to simple substrates that are subsequently used for methanogenesis (hydrogen, formate, acetate, CO_2_) or to other metabolites (e.g., lactate, ethanol, propionate, butyrate, fatty acids) that can undergo secondary fermentation yielding the aforementioned simple methanogenic substrates (17–19, 21–23).

Secondary consumers in these consortia, living under anaerobic conditions with hydrocarbons as the primary carbon source, have thus far been overlooked (20), and their additional functions in the consortia have remained hidden. Some first predictions can be made based on the Black Queen hypothesis that suggests a role of these secondary consumers as possible providers of essential nutrients to salvage other consortium members from auxotrophies. The Black Queen hypothesis (24) states that the loss of certain energy-expensive functions is an adaptation to a cooperative lifestyle under conditions of nutrient and energy limitation. Accordingly, it has been suggested that the inability of individual community members to synthesize the full range of organic nutrients required for growth may be a favorable trait for communities and consortia operating under energy-limited conditions (25).

Some pioneering work to systematically test interactions and dependencies between auxotrophs has been done using two-member to multimember *Escherichia coli* communities (26–29). Those studies mostly considered pairs of populations interacting in isolation but did not account for the fact that natural communities are composed of a diverse set of interacting populations. Since the number of potential interactions grows dramatically with the number of types in a community, characterizing interactions in such diverse assemblages has proven to be a major challenge (30). Reconstructing metabolic pathways and assigning functions among community members using genomics are now emerging as promising tools for taking the first steps toward a more holistic understanding of the functioning of microbial consortia that mediate anaerobic degradation of hydrocarbons.

## RESULTS AND DISCUSSION

For this study, we extracted high-quality genomes of known syntrophic bacteria from public databases. To identify taxa that have the potential to grow syntrophically, we used gene annotations predicting enzymes involved in reverse and direct electron transfer (see Table S1 in the supplemental material). Many syntrophic bacteria are known to have the capacity to perform reverse electron transport-driven H_2_ production through the oxidation of a low-potential donor (electron confurcation) and reduction of a high-potential acceptor (electron bifurcation), in combination with the high-energy electron carrier ferredoxin (20). Our analysis confirmed a wide distribution of membrane-bound, ion-translocating ferredoxin:NAD^+^ oxidoreductases and confurcating hydrogenases that could directly couple the oxidation of NADH and reduced ferredoxin to produce hydrogen (Table S1). Additionally, most of the putatively syntrophic bacterial isolates lacked important genes involved in known biosynthesis pathways of one or more amino acids and vitamins, indicating that they may rely on precursors (or the cofactors) provided by other bacteria to produce fully functional enzymes with required cofactors and prosthetic groups (Tables S2 and S3). However, there is still the possibility that they are capable of performing biosynthesis in ways that we currently do not know about.

10.1128/mSystems.00038-17.2 TABLE S1  Distribution of membrane-bound, ion-translocating ferredoxin:NAD^+^ oxidoreductases and confurcating hydrogenases that could directly couple the oxidation of NADH and reduced ferredoxin to produce hydrogen. Only genera that could be found in the SCADC are represented. Download TABLE S1, PDF file, 0.02 MB. Copyright © 2017 Hubalek et al. 2017 Hubalek et al. This content is distributed under the terms of the Creative Commons Attribution 4.0 International license.

10.1128/mSystems.00038-17.3 TABLE S2  Amino acid biosynthetic capabilities of selected genomes obtained from MaGe. Prototrophy predictions for each amino acid are based on the “pathway completion” value, i.e., the number of reactions for pathway x in a given organism/total number of reactions in the same pathway defined in the MetaCyc or KEGG databases. A value of 1/0 indicates the presence of all/none of the key enzymes involved in the biosynthesis. This resembles widespread auxotrophy for amino acids as inferred for the SCADC. Download TABLE S2, PDF file, 0.1 MB. Copyright © 2017 Hubalek et al. 2017 Hubalek et al. This content is distributed under the terms of the Creative Commons Attribution 4.0 International license.

10.1128/mSystems.00038-17.4 TABLE S3  Vitamin biosynthesis capabilities of selected genomes obtained from MaGe. Prototrophy predictions for each vitamin (including salvage pathways) are based on the “pathway completion” value, i.e., the number of reactions for pathway x in a given organism/total number of reactions in the same pathway defined in the MetaCyc or KEGG databases. A value of 1/0 indicates the presence of all/none of the key enzymes involved in the biosynthesis. This resembles widespread auxotrophy for vitamins as inferred for the short-chain alkane-degrading culture (SCADC). Download TABLE S3, PDF file, 0.1 MB. Copyright © 2017 Hubalek et al. 2017 Hubalek et al. This content is distributed under the terms of the Creative Commons Attribution 4.0 International license.

Next, we attempted to resolve these interactions further by isolating natural methanogenic consortia. A short-chain alkane-degrading enrichment (SCADC) from mature fine tailings of the Mildred Lake Settling Basin (12) was used to obtain 13 shotgun metagenomes from various subcultures of the original enrichment (for details on the metagenomes, see Table S4). These included eight samples, each containing approximately 10 individual filaments that had been microscopically separated from the bulk culture based on their morphology and the presence or absence of visible epibionts. The physical manipulation for sequencing was an attempt to isolate members of the consortia that exchange cofactors or their precursors when they are in close physical contact in contrast to the free-living community members with peripheral roles in alkane degradation.

10.1128/mSystems.00038-17.5 TABLE S4  Source of metagenome and summary of sequencing data. “+Epibiont” indicates samples that include filaments with epibioints, whereas “-Epibioints” indicates samples without epibionts. Download TABLE S4, PDF file, 0.02 MB. Copyright © 2017 Hubalek et al. 2017 Hubalek et al. This content is distributed under the terms of the Creative Commons Attribution 4.0 International license.

By using the eight samples with extracted filaments and an additional five bulk samples, amplicon sequencing of 16S rRNA genes revealed the identity of the community members. This analysis demonstrated that there were pronounced differences in the identities of the community members on the sorted morphotypes (i.e., with and without observable epibionts); however, no systematic associations between the two different filament types and full samples could be observed (for taxonomic composition data, see Fig. S1 in the supplemental material). Based on shotgun metagenome reads from the 13 samples, we used MEGAHIT to obtain a metagenome coassembly of 805,606 scaffolds totaling ~1 Gbp with an N50 length of 4,902 bp. Downstream coverage and nucleotide composition-based binning of the 150,698 scaffolds of at least 1,000-bp length resulted in 307 bins with 45 matching CheckM estimates of completeness (>70%) and contamination (<5%) and thus representing high-quality metagenome assembled genomes (MAGs; for details on CheckM statistics, see Table S5).

10.1128/mSystems.00038-17.1 FIG S1  Taxonomic composition of the short-chain alkane-degrading culture (SCADC) in the different samples used for shotgun metagenomic sequencing. The 16S rRNA gene data were produced following the procedure outlined by Sinclair et al. (65). Samples with pooled filaments with epibioints (epi) and without epibionts (no), as well as full sample metagenomes (full) and previously published metagenomes (published), are given. Download FIG S1, PDF file, 0.1 MB. Copyright © 2017 Hubalek et al. 2017 Hubalek et al. This content is distributed under the terms of the Creative Commons Attribution 4.0 International license.

10.1128/mSystems.00038-17.6 TABLE S5  CheckM (doi: 10.1101/gr.186072.114) statistics for the 307 bins obtained in this study. High-quality metagenome assembled genomes (MAGs) are defined based on completeness estimates of >70% and contamination of <5% and highlighted in red. Download TABLE S5, PDF file, 0.1 MB. Copyright © 2017 Hubalek et al. 2017 Hubalek et al. This content is distributed under the terms of the Creative Commons Attribution 4.0 International license.

**FIG 1:**
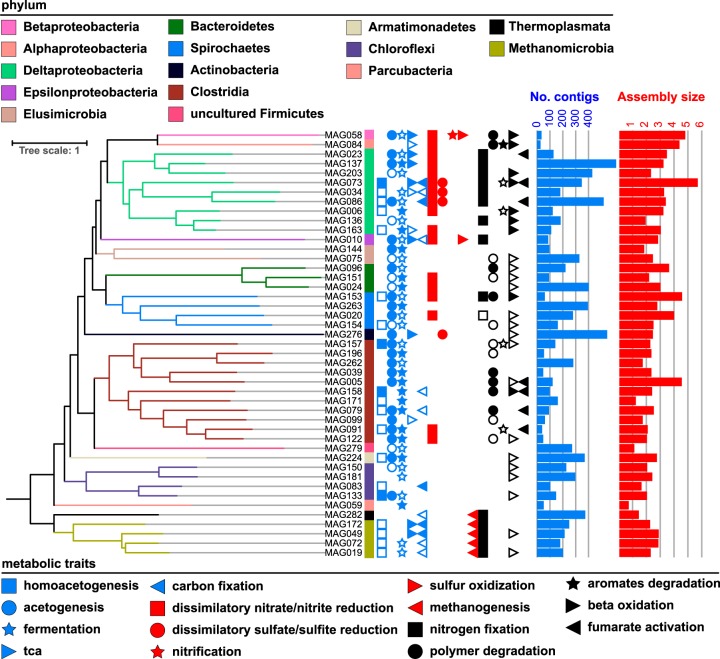
A phylogenomic tree of recovered genomes as computed by PhyloPhlAn (61). The color strip delineates taxonomic affiliation. The symbols show the metabolic traits based on genome annotations. Traits with high support are indicated by closed symbols, while traits with low support have open symbols, and categories with missing symbols indicate that no indications of the trait were found. The two bar charts on the far right show the number of contigs assigned to each bin and the assembly size (in mega-base pairs) contained in the recovered genome bins. tca, tricarboxylic acid cycle.

As indicated by detailed annotations of these MAGs using the “MicroScope” pipeline (31), the most likely hydrocarbon degraders in the culture were putative syntrophic bacteria related to *Syntrophus* (23, 32), *Pelotomaculum* (33, 34), *Desulfobacterium* (34, 35), and *Clostridium* (36, 37), all having the potential for fumarate activation, betaoxidation, and fumarate regeneration. The genes indicative of these pathways encoded alkylsuccinate synthase and benzylsuccinate synthase (C-H activation), methylmalonyl-CoA mutase (carbon skeleton rearrangement), and methylmalonyl-CoA decarboxylase (decarboxylation) for n-alkane activation; enoyl-CoA hydratase, β-hydroxyacyl-CoA dehydrogenase, and acyl-CoA acetyltransferase for beta oxidation; and propionyl-CoA carboxylase, methylmalonyl-CoA mutase, succinyl-CoA synthetase, and succinate dehydrogenase for fumarate regeneration (13). Most genomes encoded enzymes associated with fermentation and coupled pathways for acetogenesis (i.e., reductive acetyl-CoA or Wood-Ljungdahl pathway), a process by which acetate is produced from CO_2_ and an electron donor (Fig. 1). The five methanogen genomes reconstructed from the contig bins belonged to the genera *Methanoculleus* and *Methanosaeta* in the class *Methanomicrobia* and to members of the class *Thermoplasmata*. As previously described, the members of *Methanosaeta* represent acetoclastic methanogens whereas the members of *Methanoculleus* represent hydrogenotrophic methanogens and the members of *Thermoplasmata* represent methylotrophic methanogens (38). Besides variable substrates used for methane production, these microbes all encode complete sets of nitrogen fixation genes. For detailed exploration of the genomes, we direct the reader to the online sources of the “MicroScope” pipeline (31), where individual KEGG and biocyc maps can be inspected for each genome.

**FIG 2:**
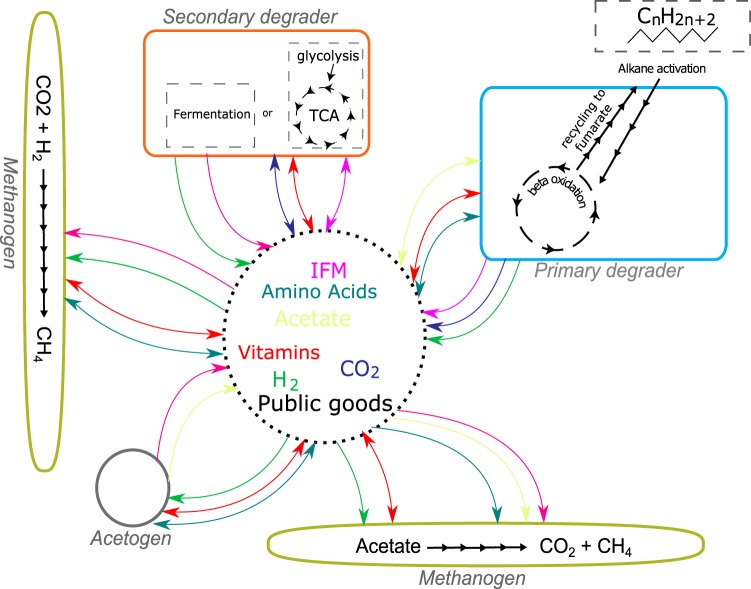
Model of potential metabolic pathways and metabolic cross-feeding in the short-chain alkane-degrading culture (SCADC) representing (green) archaeal filaments, (blue) alkane degraders, (red) intermediate metabolite scavengers and secondary degraders, and (gray) acetogenic bacteria. Arrows show the inferred syntrophic interactions corresponding to transfer of hydrogen (green), carbon dioxide (dark blue), and acetate (light green) to intermediate fermentation metabolites (IFMs; violet) as well as other forms of metabolic interdependencies such as cross-feeding of costly metabolites such as vitamins (red) and amino acids (teal).

In addition to the classical syntrophic interactions described in hydrocarbon-degrading communities, i.e., where one partner produces metabolites such as H_2_, acetate, ethanol, and/or small organic intermediates which are then used by methanogens and secondary degraders, our genomic reconstructions point to additional metabolic dependencies. First, the scavenging of metabolic products derived from detrital biomass is indicated by the presence of genes coding for transporters for a wide diversity of proteins and fatty acids, as well as genes involved in the degradation of intermediate fermentation products (i.e., C1 compounds and alcohols) (Fig. 2). Genomes of these proposed secondary degraders belonged to the deltaproteobacterial genera *Desulfovibrio*, *Desulfomicrobium*, and *Desulfobulbus*, previously reported to use H_2_, organic acids, formate, or short-chain alcohols as electron donors for sulfate reduction (39–41). In addition to their potential for using anaerobic respiration, these genera possessed genes associated with fermentation of a wide range of alcohols and organic acids such as lactate and amino and carboxylic acids, suggesting that they were actually able to grow under these conditions and were not merely remnants from early stages of the culture.

**FIG 3:**
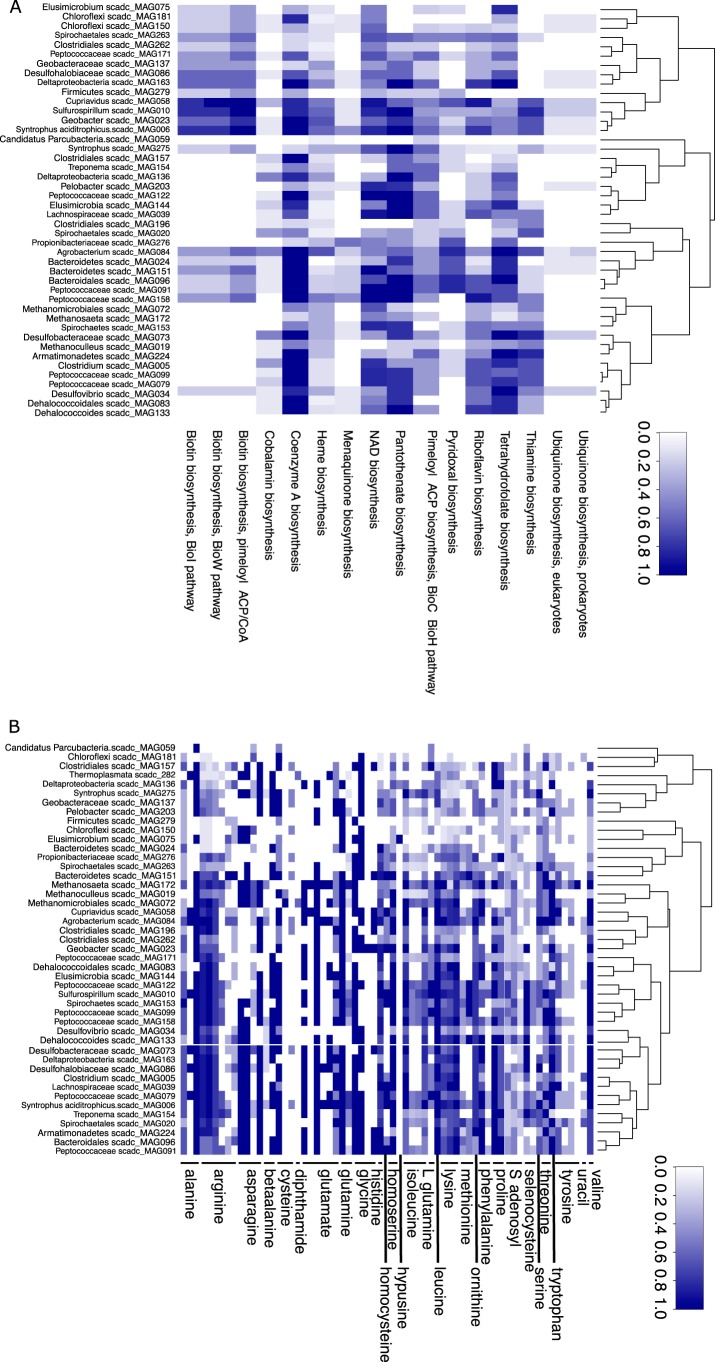
Heat map of vitamin (A) and amino acid (B) biosynthetic capabilities of metagenome assembled genomes (MAGs) obtained from the short-chain alkane-degrading culture (SCADC). Prototrophy predictions for each amino acid and vitamin are based on the “pathway completion” value, i.e., the number of reactions for pathway x in a given organism/total number of reactions in the same pathway defined in the MetaCyc or KEGG databases. A value of 1/0 indicates that all/none of the key enzymes were involved in the biosynthesis. Dendrograms represent clustering based on potential biosynthesis profiles. To verify widespread auxotrophy for vitamins and amino acids in syntrophic prokaryotes, a similar analysis was performed using genomes available from genera representing our taxonomic bins (for details, see Fig. S1). As a definition of absence, we used a defensible threshold value for pathway completeness of less than 0.1.

Like many of their cultivated and genome-sequenced relatives, the MAGs from the putative sulfate reducers in these consortia revealed auxotrophies for vitamins and amino acids (Fig. 3). The absence of such pathways, defined as lacking more than half of the genes in the pathways, needs to be interpreted with caution, because of the partial nature of the individual MAGs and since there are many open reading frames (ORFs) that cannot be assigned to a function. Still, analogous auxotrophies in closely related MAGs and closed genomes from public databases provide strong support for the idea of widespread auxotrophy. This reliance on essential metabolites hints at the current inability to readily isolate the majority of anaerobic microbes in pure culture, which is true for most environmental microbes (42). Examples of essential metabolite pathways whose biosynthetic gene distribution is patchy in the SCADC MAGs are those corresponding to biosynthesis of menaquinones (vitamin K) and ubiquinones (coenzyme Q), cofactors in multiple respiratory reactions. Other examples are vitamins B6 (pyridoxal), B12 (adenosylcobalamin), and H (biotin). Earlier large-scale genome sequence analyses previously suggested that corrinoids, essential cofactors for adenosylcobalamin (43), are widely shared as “common goods” by over 70% of the sequenced bacterial species, while less than 25% of species synthesize corrinoids *de novo* (44, 45). The existence of over a dozen structurally distinct corrinoids produced and used by various *Bacteria* and *Archaea* (46) can thus play a central role in dictating microbial community assembly and functioning, since the structure of the precursor, which can be a benzimidazole or a purine or phenolic compound (46), influences the degree to which a particular corrinoid can be used by a given organism (47, 48).

Likewise, the absence of genes underlying biosynthesis of essential amino acids is apparent in particular taxonomic groups (Fig. 3B). The loss of essential biosynthesis function in various taxonomic groups has previously been associated with energy limitation and nutrient limitation selecting for small cells with reduced genomes such as are found in the open ocean and deep biosphere (49, 50). Particularly costly functions are absent in most members of the SCADC community, resulting in a community critically depending on essential metabolite providers. Biosynthesis of certain vitamins such as adenosylcobalamin and of specific amino acids such as phenylalanine, tyrosine, and tryptophan is energetically costly and requires a large set of genes (51). This loss of function and the presence of subsequent adaptations to a cooperative lifestyle under conditions of nutrient limitation and energy limitation form the foundation for the Black Queen hypothesis (24).

In the context of the SCADC, the Black Queen hypothesis implies that, in addition to quenching of fermentation products by methanogens, other types of public goods such as amino acids and vitamins can be exchanged (Fig. 2). Such public goods are provided by a few taxa carrying the enzymes to perform this energetically costly biosynthesis while benefitting other organisms in their surroundings. The idea of this enhanced cross feeding is logical if one considers the need for metabolic processes to be optimized for reduced metabolic burden at the level of the individual cell living with extreme energy limitations.

We are aware that genomic data have limitations, due to the fact that there are large numbers of poorly understood genes and that these genes may not necessarily be functional even if they are annotated with existing gene models. Furthermore, we cannot reject the notion that the biosynthesis potentials for one or the other metabolite may be biased and that the resulting interactions may thus represent nothing more than genomic predictions. Nevertheless, we were able to show in this study that the level of interactions between the components of syntrophic alkane degrader-methanogen partnerships surpasses those of the previously suggested standard electron transfer and simple carbon compound transfer interactions. The levels of biosynthesis of vitamins and other energy-expensive metabolites seem to be unequally distributed among community members, and we propose that this facilitates energy conservation. It can be further speculated that vitamin and amino acid additions might enhance petroleum bioaugmentation processes in remediation of hydrocarbon-contaminated sites.

## MATERIALS AND METHODS

### Culturing.

A short-chain alkane-degrading culture (SCADC) was enriched from mature fine tailings of the Mildred Lake Settlings Basin as detailed by Tan et al. (12). In short, the primary enrichment cultures were established in multiple replicates by adding 25 ml oil sand tailings to 50 ml of mineral methanogenic medium (52) under a headspace of O_2_-free 30% CO_2_-N_2_ balance gas. After an initial 1-year incubation in the dark at room temperature, the primary cultures were pooled and used to inoculate multiple replicates of first-transfer cultures into fresh methanogenic medium. After a second 6-month incubation, these cultures were pooled as an inoculum for the experimental SCADC enrichment cultures, where 0.1% (vol/vol) of an alkane mixture comprising equal volumes of C6 and C9 n-alkanes was added to the community and the resulting reaction mixture was incubated for at least 4 months at 28°C in the dark, prior to further subculturing (12). The samples analyzed in the present study were from several transfers of this single parent SCADC culture. Three bottles containing 75-ml cultures were each sampled three times using micromanipulation, originally to capture the filamentous bacteria seen to carry attached smaller cocci. Fluorescence in situ hybridization (FISH) using probes MX825b and MX825c (53) revealed that most of the filaments represented *Methanosaeta* with associated bacterial cells.

### Filament sampling.

Micromanipulation was performed using an Eppendorf TransferMan NK 2 micromanipulator at the Department of Microbial Ecology, University of Vienna. Each slide used for micromanipulation was prepared by cleaning with DNA Away surface decontaminant (Thermo, Fisher Scientific) and rinsed in sterile phosphate-buffered saline. Filaments were picked from a 12-µl culture subsample by using a Zeiss Axio Observer.D1 microscope at ×32 magnification, captured in a 4-µm-diameter capillary (TransferTip; Eppendorf), and subsequently transferred into a sterile plastic tube. Approximately 10 filaments were collected from each of eight cultures and pooled according to the presence of coherent morphology.

Samples with pooled filaments (*n* = 8) were then lysed by subjecting them to four freeze-thaw cycles, consisting of freezing at −80°C followed by heat treatment at 70°C for 5 min. These samples were subjected to whole-genome amplification with multiple-displacement amplification (MDA) using a REPLI-g minikit according to the instructions of the manufacturer (Qiagen).

### Whole-culture DNA isolation.

A 100-ml volume from the SCADC culture was filtered through 0.2-µm-pore-size Supor membrane disc filters (Pall). Prior to bead beating, the above-mentioned freeze-thaw cell lysis was conducted, as preceding tests had shown that the filaments needed a more vigorous treatment. The DNA was then isolated from the filters by bead beating using a PowerSoil DNA isolation kit (MoBio) according to the manufacturer’s instructions and was sequenced as described below. No whole-genome amplification with MDA was performed on these samples, except in one case.

### Library preparation and sequencing.

The SciLifeLab SNP/SEQ sequencing facility at Uppsala University performed the library preparation and the sequencing. First, 10 ng of genomic DNA was sheared using a focused ultrasonicator (Covaris E220). The sequencing libraries were prepared using a Thruplex FD Prep kit (Rubicon Genomics) according to manufacturer’s protocols (R40048-08 and QAM-094-002). Library size selection was performed using AMPpure XP beads (Beckman Coulter, Inc.) at a 1:1 ratio with the DNA sample volume. The prepared sample libraries were quantified by using a Kapa Biosystems next-generation sequencing library quantitative PCR (qPCR) kit and by performing analyses using a StepOnePlus (Life Technologies, Inc.) real-time PCR instrument. The quantified libraries were then prepared for sequencing on an Illumina HiSeq sequencing platform with a TruSeq paired-end cluster kit (v3) and Illumina’s cBot instrument to generate a clustered flow cell for sequencing. Sequencing of the flow cell contents was performed using an Illumina HiSeq2500 sequencer with an Illumina TruSeq SBS sequencing kit (v3), following a 2-by-100 indexed high-output run recipe.

### Assembly and binning of metagenome reads.

Reads were filtered based on their quality scores using Sickle (version 1.210) (54). The read set was digitally normalized using Khmer (55) and subsequently assembled with MEGAHIT (56). Contigs were merged into scaffolds by the use of BESST software (57). Coverage was computed by mapping the reads back to the scaffolds using BBMap (B. Bushnell; http://sourceforge.net/projects/bbmap/), and for computing coverage, BEDTools (version 2.18.2) (58) was used. Scaffolds were binned using MetaBAT (59), and the criteria for high-quality bins (corresponding to the so-called MAGs) were based on CheckM (60) estimates of completeness (>70%) and contamination (<5%).

### Processing of mined data.

In addition to the data acquired from the newly sequenced samples, we also accessed three data sets from the same original SCADC, downloaded from the NCBI Short Read Archive (SRA): SRR636559 (a high-depth HiSEQ data set) and SRR634694 and SRR634695 (two Roche 454 data sets). These archival sequences were treated bioinformatically in the same way as the new sequences and were coassembled with them.

### Annotation.

Initial phylogenetic attribution of the MAGs was performed using PhyloPhlAn (61) on proteins predicted with Prokka (version 1.9) (62), supplemented with a custom set of MAGs, single amplified genomes (SAGs), and whole-genome sequences accessed from JGI IMG/M. PFAM annotations were performed using the Pfam database (27.0) (63) and HMMER (64) (version 3.1b1) with default parameters. Detailed genome annotation analyses were performed using the “MicroScope” pipeline (31) with automatic annotations assisted by manual curation as described in the integrated bioinformatics tools and the proposed annotation rules. Reconstructed MAGs were deposited on the MicroScope platform with the identifier “scadc_MAG” and in the European Nucleotide Archive (see below).

### Accession number(s).

Data acquired from the newly sequenced samples were deposited in the European Nucleotide Archive (accession numbers SAMEA104032520 to SAMEA104032529). The full project can be found under accession number PRJEB20591.
