# Supplementary material for: Vitamin and Amino Acid Auxotrophy in Anaerobic Consortia Operating under Methanogenic Conditions
Source: mSystems. 2017 Oct 31;2(5):e00038-17. doi: 10.1128/mSystems.00038-17 (PMC5663940; doi:10.1128/mSystems.00038-17)
Supplement: TABLE S1 [file sys005172144st2.pdf]

genus	Fnr, ion-translocating ferredoxin:NAD+ oxidoreductase	Confurcating H2ase	Direct electron transfer	FeS oxidoreductase	Membranebound FDH	Membranebound H2ase	NADH-linked FDH	Other FDH	Other soluble H2ase	putative electron transfer flavoprotein:quinone oxidoreductase
unclassified	X	X	X	X	X	X	X	X	X	X
Acholeplasma	X	X		X		X	X	X	X	X
Agrobacterium	X	X		X	X	X	X	X	X	X
Anaerolinea	X	X		X	X	X	X	X	X	X
Anaeromyxobacter	X			X			X		X	X
Clostridium	X	X		X		X	X	X	X	X
Cupriavidus	X	X		X	X	X	X	X	X	X
Dehalogenimonas	X	X		X	X	X	X	X	X	X
Desulfobacterium		X		X	X	X	X	X	X	
Desulfobulbus	X	X		X	X	X	X	X	X	X
Desulfomicrobium	X				X	X	X	X	X	
Desulfosporosinus	X	X		X	X	X	X	X	X	X
Desulfovibrio	X	X			X	X	X	X	X	
Geobacter	X	X		X	X	X	X	X	X	X
Marinilabilia	X	X		X	X	X	X	X	X	X
Mesotoga	X	X		X		X	X	X	X	X
Methanoculleus	X	X			X	X	X		X	
Methanolinea	X				X	X	X	X	X	
Methanomassiliicoccus		X				X	X		X	
Methanoregula	X			X	X	X	X	X	X	
Methanosaeta	X	X		X	X	X	X	X	X	
Methanosphaerula		X			X	X	X	X	X	
Methanospirillum	X	X				X	X	X	X	
Paludibacter	X	X		X		X	X	X	X	X
Pedosphaera	X			X		X			X	
Pelobacter	X	X	X	X	X	X	X	X	X	X
Pelotomaculum	X	X		X	X	X	X	X	X	X
Sphaerochaeta	X	X		X		X	X		X	X
Sulfurospirillum	X	X			X	X	X	X	X	
Synergistes	X			X			X		X	X
Syntrophomonas						X	X		X	
Syntrophus	X	X		X	X	X	X	X	X	X
Thiobacillus	X	X			X	X	X	X	X	
