# Supplementary material for: Vitamin and Amino Acid Auxotrophy in Anaerobic Consortia Operating under Methanogenic Conditions
Source: mSystems. 2017 Oct 31;2(5):e00038-17. doi: 10.1128/mSystems.00038-17 (PMC5663940; doi:10.1128/mSystems.00038-17)
Supplement: TABLE S2 [file sys005172144st3.pdf]

	alanine	arganine	asparagine	asparate	cysteine	glutamate	glutamine	glycine	histidine	homocysteine	homoserine	isoleucine	L-glutamine	leucine	lysine	methionine	ornithine	phenylalanine	proline	serine	threonine	tryptophan	tyrosine	valine
Agrobacterium albertimagni AOL15	1	1	1	1	1	1	1	1	0.9	0.5	1	1	0.5	1	1	1	1	1	1	1	1	1	1	1
Agrobacterium arsenijevicii KFB 330	1	1	1	1	1	1	1	1	0.9	0.5	1	1	0.5	1	1	0.8	1	1	1	1	1	1	0.67	1
Agrobacterium radiobacter DSM 30147	1	0.89	0.67	1	1	1	1	1	0.9	0.5	0.67	0.6	1	0.8	0.78	0.2	0.8	0.67	0.33	1	0.5	0.83	1	0.5
Agrobacterium radiobacter K84	0.67	0.78	1	1	1	1	1	1	1	0.5	0.67	0.6	0.5	1	0.89	0.8	0.6	1	1	1	1	1	1	1
Agrobacterium rhizogenes K599	1	0.89	0.33	1	1	1	1	1	0.6	0.5	1	1	0	0.4	0.71	0.6	1	0.33	0.33	1	1	0.5	0.33	1
Agrobacterium scadc_MAG084	1	1	0.33	1	1	1	1	1	0.9	0.5	1	1	0	1	1	0.6	1	0.67	1	1	1	1	0.67	1
Agrobacterium sp. 10MFCol1.1	1	1	1	1	1	1	1	1	0.9	0.5	1	1	0.5	1	1	0.8	1	1	1	1	1	1	0.67	1
Agrobacterium sp. 224MFTsu3.1	1	1	0.67	1	1	1	1	1	1	0.5	1	0.8	1	0.8	1	0.8	1	1	1	1	1	1	1	0.75
Agrobacterium sp. 33MFTa1.1	1	1	0.67	1	1	1	1	1	0.9	0.5	1	1	0.5	1	1	0.6	1	0.67	1	1	1	1	0.67	1
Agrobacterium sp. H13-3	1	1	1	1	1	1	1	1	0.9	0.5	1	1	0.5	1	1	0.8	1	1	1	1	1	1	0.67	1
Agrobacterium sp. KFB 330	1	1	1	1	1	1	1	1	0.9	0.5	1	1	0.5	1	1	0.8	1	1	1	1	1	1	0.67	1
Agrobacterium sp. LC34	1	1	0.67	1	1	1	1	1	0.9	0.5	1	1	0.5	1	1	0.8	1	0.67	1	1	1	1	0.67	1
Agrobacterium sp. R89-1	1	1	0.33	1	1	1	1	1	0.9	0.5	1	1	0.5	1	1	0.8	1	0.67	1	1	1	1	0.67	1
Agrobacterium sp. SUL3	1	1	0.67	1	1	1	1	1	0.9	0.5	1	1	0.5	1	1	0.8	1	1	1	1	1	1	1	1
Agrobacterium sp. UNC420CL41Cvi	1	1	0.67	1	1	1	1	1	0.9	0.5	1	0.8	0.5	0.8	1	0.8	1	0.67	1	1	1	1	0.67	0.75
Agrobacterium tumefaciens 5A v2	1	1	1	1	1	1	1	1	0.9	0.5	1	1	0.5	1	1	0.8	1	0.67	1	1	1	1	0.67	1
Agrobacterium tumefaciens Ach5	1	1	1	1	1	1	1	1	1	0.5	1	1	0.5	1	0.89	0.8	1	0.67	1	1	1	1	0.67	1
Agrobacterium tumefaciens C58	1	1	0.33	1	1	1	1	1	0.9	0.5	1	1	0.5	1	1	0.8	1	1	1	1	1	1	0.67	1
Agrobacterium tumefaciens F2	1	1	1	1	1	1	1	1	0.9	0.5	1	1	0.5	1	1	0.8	1	0.67	1	1	1	1	0.67	1
Agrobacterium tumefaciens GW4	1	1	0.67	1	1	1	1	1	0.9	0.5	1	0.8	0.5	0.8	1	0.8	1	0.67	1	1	1	0.83	0.67	0.75
Agrobacterium tumefaciens LBA4404	1	1	0.67	1	1	1	1	1	0.9	0.5	1	1	0.5	1	1	0.8	1	1	1	1	1	1	0.67	1
Agrobacterium tumefaciens S2	1	1	1	1	1	1	1	1	1	0.5	1	1	0.5	1	1	0.8	1	1	1	1	1	1	0.67	1
Agrobacterium tumefaciens S33	1	1	0.33	1	1	1	1	1	1	0.5	1	1	0	1	0.86	0.8	1	0.67	1	1	1	1	0.67	1
Agrobacterium tumefaciens WRT31	1	1	1	1	1	1	1	1	1	0.5	1	1	0.5	1	1	0.8	1	1	1	1	1	1	0.67	1
Agrobacterium vitis NCPPB 3554	1	1	0.67	1	1	1	1	1	0.9	0.5	1	1	0.5	1	0.89	0.6	1	1	1	1	1	1	1	1
Agrobacterium vitis S4	1	0.89	0.33	1	1	1	1	1	0.9	0.5	0.67	0.8	0.5	1	0.78	0.6	0.8	1	1	1	1	0.83	1	1
Clostridiales scadc_MAG157	1	0.22	1	1	1	0	0	1	0.1	0	0.67	0.6	1	0.2	0.33	0.2	0.2	0.67	1	0.33	1	0.17	0.33	0.75
Clostridiales scadc_MAG196	1	1	0	1	0.5	1	1	1	0.1	0.5	1	0.6	0	0.4	0.44	0.2	0.8	0	0.33	0.67	0.5	0.17	0	0.5
Clostridiales scadc_MAG262	1	0.56	0	1	0.5	1	1	1	0.4	0.5	0.33	0.6	0	0.6	0.43	0	0.6	0	0.67	0.33	0	0.5	0	0.75
Clostridium acetobutylicum ATCC 824	1	1	1	1	1	1	1	1	1	0.5	1	1	0.5	1	1	0.8	0.8	0.67	1	0.67	1	1	0.33	1
Clostridium acetobutylicum DSM 1731	1	1	1	1	1	1	1	1	1	0.5	1	1	0	1	1	1	0.8	0.67	1	0.67	1	1	0.33	1
Clostridium acetobutylicum EA 2018	1	1	1	1	1	1	1	1	0.9	0.5	1	1	0.5	1	1	0.8	0.8	0.67	1	0.67	1	1	0.33	1
Clostridium acidurici 9a	1	1	1	1	1	1	1	1	1	0.5	1	1	0.5	1	0.71	0.6	0.8	1	1	0.33	1	1	0.67	1
Clostridium autoethanogenum DSM 10061	1	1	1	1	1	1	1	1	1	0	1	1	0.5	1	1	0.4	1	0.67	1	0.33	1	1	0.67	1
Clostridium bartlettii CAG 1329	1	1	1	1	1	1	1	1	0.9	0.5	1	0.8	1	0.8	0.86	0.2	0.8	0.33	1	0	1	0.33	0.67	0.75
Clostridium beijerinckii G117	1	1	1	1	1	1	1	1	0.9	1	1	1	0.5	1	0.86	0.6	0.8	1	1	0.67	1	1	0.67	1
Clostridium beijerinckii NCIMB 8052	1	0.89	1	1	1	1	1	1	1	0.5	1	1	0.5	1	0.86	0.8	0.8	0.67	1	0.67	1	1	0.67	1
Clostridium beijerinckii NRRL B-598	1	0.89	1	1	1	1	1	1	0.9	0.5	1	1	0.5	1	0.89	1	0.8	0	1	0.33	1	1	0.67	1
Clostridium bifermentans ATCC 19299	1	0.22	1	1	1	0	0	1	0.2	0	0.67	0.2	0.5	0	1	0.2	0	0.33	0.33	0	0	0.17	0.33	0
Clostridium bifermentans ATCC 638	1	0.22	1	1	1	0	0	1	0.2	0	0.67	0.2	0.5	0	1	0.2	0	0.33	0.33	0	0	0.17	0.33	0
Clostridium botulinum A ATCC 19397	1	0.33	1	1	1	1	1	1	1	0.5	1	0.6	0	0.2	1	0.6	0	0.33	0.33	0.33	1	0	0.33	0.25
Clostridium botulinum A ATCC 3502	1	0.44	1	1	1	1	1	1	1	0.5	1	0.6	0	0.2	1	0.6	0	0.33	0.33	0.33	1	0	0.33	0.25
Clostridium botulinum A Hall	1	0.33	1	1	1	1	1	1	0.8	0.5	1	0.6	0.5	0.2	0.71	0.6	0	0.33	0.33	0.33	1	0	0.33	0.25
Clostridium botulinum B1 Okra	1	0.44	1	1	1	1	1	1	1	0.5	1	0.6	0	0.2	1	0.6	0	0.33	0.33	0.33	1	0	0.33	0.25
Clostridium butyricum 5521	1	1	1	1	1	1	1	1	0.9	1	1	1	0.5	1	1	0	0.8	0.67	1	1	1	0.83	0.33	1
Clostridium butyricum 60E.3	1	1	1	1	1	1	1	1	0.8	1	1	1	0.5	1	1	0.6	0.8	0.67	1	0.67	1	1	0.33	1
Clostridium butyricum DKU-01	1	1	1	1	1	1	1	1	0.9	1	1	1	0.5	1	1	0.4	0.8	0.67	1	1	1	1	0.33	1
Clostridium butyricum E4 BoNT E BL5262	1	1	1	1	1	1	1	1	0.9	1	1	1	0.5	1	1	0.2	0.8	0.67	1	1	1	1	0.33	1
Clostridium carboxidivorans P7	1	0.67	1	1	1	1	1	1	0.8	1	1	1	0.5	1	1	0.4	0.4	1	1	0.33	1	1	0.67	1
Clostridium carboxidivorans P7 PRJNA29495	1	0.78	1	1	1	1	1	1	0.8	0.5	1	0.8	0.5	0.8	0.86	0.4	0.6	0.67	1	0.33	1	1	0.67	0.75
Clostridium celatum DSM 1785	1	1	1	1	1	1	1	1	0.8	1	1	1	0	1	0.86	0.2	0.8	0.67	0.67	0	1	0.17	0.67	1
Clostridium cellulolyticum H10; ATCC 35319	1	0.89	1	1	1	1	1	1	1	0.5	1	0.8	0.5	1	0.71	0.2	0.8	0.67	1	0.33	0.5	1	0.67	1
Clostridium cellulovorans 743B	1	1	1	1	1	1	1	1	0.9	0.5	1	1	0.5	1	1	0.4	1	0.67	1	0.67	1	1	0.67	1
Clostridium cellulovorans 743B PRJNA52819	1	0.89	1	1	1	1	1	1	0.8	0.5	1	1	0.5	1	0.86	0.4	0.6	1	1	0.33	1	1	0.67	1
Clostridium citroniae WAL-17108	1	1	1	1	1	1	1	1	0.7	0.5	1	1	0.5	1	0.86	0	0.8	0.33	1	0.33	0	1	0	1
Clostridium difficile 630	1	0.89	1	1	1	1	1	1	1	0.5	1	0.8	0	0.8	0.86	0.6	0.8	0.67	0.33	0.67	1	0	0.67	0.75
Clostridium difficile CD196	1	0.89	1	1	1	0	1	1	1	0.5	1	0.8	0	0.8	1	0.6	0.8	1	0.33	0.67	1	0	1	0.75
Clostridium difficile MID11 7032989	1	0.89	1	1	1	1	1	1	1	0.5	1	0.8	0	0.8	0.86	0.6	0.8	0.67	0.33	0.33	1	0	0.33	0.75
Clostridium difficile MID12 7032985	1	0.89	1	1	1	1	1	1	1	0.5	1	0.8	0	0.8	0.86	0.6	0.8	0.67	0.33	0.33	1	0	0.67	0.75
Clostridium difficile MID13 7032994	1	1	1	1	1	1	1	1	1	0.5	1	0.8	0.5	0.8	0.86	0.6	1	0.67	0.33	0	1	0.17	0.67	0.75
Clostridium difficile R20291	1	1	1	1	1	0	1	1	1	0.5	1	0.8	0	0.8	1	0.6	1	1	0.33	0.67	1	0	1	0.75
Clostridium hathewayi CAG:224	1	1	1	1	1	1	1	1	0.8	1	1	1	0.5	1	0.86	0.4	0.8	0.33	1	0.67	0.5	0.67	0.33	1
Clostridium hiranonis DSM 13275	1	0.33	1	1	1	1	1	0	0.2	0.5	0.67	0.8	0.5	0.2	0.86	0.4	0	0.33	0.33	0	0	0	0.33	0.75
Clostridium hylemonae DSM 15053	1	1	1	1	1	1	1	1	0.9	0.5	1	1	1	1	0.86	0.2	0.8	0.67	1	0.33	0.5	0.17	0.33	1
Clostridium kluyveri DSM 555	1	0.67	1	1	1	1	0	1	1	0.5	0.67	1	0	1	0.71	0.8	0.6	0.67	1	0.33	1	1	0.33	1

Clostridium kluyveri NBRC 12016	0.67	0.78	1	1	0	1	0	1	0.8	0	0.67	0.8	0	0.8	0.57	0.4	0.8	0	0.33	0	1	0.83	0	0.75
Clostridium lentocellum DSM 5427	1	1	1	1	1	1	1	1	1	0	1	1	0.5	1	1	0.6	1	1	1	1	1	1	1	0.75
Clostridium leptum CAG 27	1	1	1	1	1	1	1	1	0.9	0.5	1	0.8	0.5	1	1	0	0.8	0.33	1	0.33	1	1	0.33	1
Clostridium leptum DSM 753	1	0.89	1	1	1	1	1	1	0.9	0.5	1	0.8	0.5	1	1	0	0.8	0.33	1	0.33	1	1	0.33	1
Clostridium ljungdahlii ATCC 49587	1	1	1	1	1	1	1	1	1	0.5	1	1	0.5	1	1	0.6	0.8	0.67	1	0.67	1	1	1	1
Clostridium nexile CAG:348	1	1	1	1	1	1	1	1	0.6	0.5	1	0.8	0	1	0.86	0	0.8	0.33	1	0.33	0.5	1	0	1
Clostridium novyi NT	1	1	1	1	1	1	1	1	0.9	0.5	1	1	0.5	0.2	0.86	0.4	0.8	1	1	0.67	1	0.5	0.67	1
Clostridium papyrosolvens C7	1	1	1	1	1	1	1	1	1	1	1	0.8	0.5	1	1	0.2	0.8	0.67	1	0.67	0.5	1	0.67	1
Clostridium pasteurianum BC1	1	1	1	1	1	1	1	1	0.9	0.5	1	1	0	1	1	0.8	1	0.67	1	0.33	1	1	0.67	1
Clostridium pasteurianum DSM 525	1	1	1	1	1	1	1	1	0.8	1	1	1	0.5	1	1	0.6	0.8	1	1	0.67	1	1	0.67	1
Clostridium perfringens ATCC 13124	1	0.44	1	1	1	1	1	1	0.2	0	0.67	0.4	0	0.2	0.86	0.2	0	0.67	1	0.33	0	0	0.33	0.25
Clostridium perfringens SM101	1	0.33	1	1	1	1	1	1	0.1	0	0.67	0.4	0	0.2	0.89	0.2	0	0.67	1	0.33	0	0	0.33	0.25
Clostridium perfringens str. 13	1	0.44	1	1	1	1	1	1	0.2	0	0.67	0.4	0	0.2	0.86	0.2	0	0.67	1	0.33	0	0	0.33	0.25
Clostridium phytofermentans ISDg	1	1	1	1	1	1	1	1	1	0.5	1	1	0.5	1	1	0.6	1	0.33	1	0.67	0.5	1	0.33	1
Clostridium saccharobutylicum DSM 13864	1	1	1	1	1	1	1	1	1	0	1	1	0.5	1	0.86	0.6	0.8	0.67	1	1	1	1	0.67	1
Clostridium saccharolyticum WM1	1	1	1	1	1	1	1	1	0.9	0	1	1	0.5	1	1	0.4	1	0.33	1	0.67	0.5	1	0.33	1
Clostridium saccharoperbutylacetonicum N1-4(HMT)	1	1	1	1	1	1	1	1	1	0.5	1	1	0.5	1	0.86	0.8	0.8	1	1	1	1	1	1	1
Clostridium saccharoperbutylacetonicum N1-4(HMT) ATCC 27021	1	1	1	1	1	1	1	1	0.8	1	1	1	0.5	1	0.89	0.4	0.8	1	1	1	1	1	1	1
Clostridium scadc_MAG005	1	1	1	1	1	1	1	1	0.9	0.5	1	1	0.5	1	1	0	0.8	0.33	1	0.33	0.5	0.67	0.33	1
Clostridium sp. ASF356	1	1	1	1	1	1	1	1	0.7	0.5	1	0.8	0.5	0.8	1	0.4	0.8	0.67	1	0.67	1	0.5	0.33	0.75
Clostridium sp. ASF502	1	1	1	1	1	1	1	1	0.8	0.5	1	1	0.5	1	0.86	0.2	0.8	0.67	1	0.33	0.5	1	0.33	1
Clostridium sporogenes ATCC 15579	1	0.44	1	1	1	0	1	1	0.8	0	1	0.6	0.5	0.2	0.86	0.6	0	0.67	0.33	0	1	0	0.33	0.5
Clostridium sporogenes PA 3679	1	0.44	1	1	1	0	1	1	0.8	0	1	0.6	0.5	0.2	0.86	0.6	0	0.67	0.33	0.33	1	0	0.33	0.5
Clostridium sticklandii DSM 519	1	0.89	1	1	1	1	1	1	1	0.5	1	1	0	1	0.86	0.4	0.6	1	0.33	0.67	1	0.83	0.67	1
Clostridium symbiosum ATCC 14940	1	1	1	1	1	1	1	0	0.2	0.5	1	1	0.5	1	0.86	0.4	0.8	0.67	1	0.67	0.5	1	0.67	1
Clostridium termitidis CT1112 CT1112	1	1	1	1	1	1	1	1	1	1	1	0.8	0.5	1	1	0.6	0.8	0.67	1	0.67	0.5	1	0.67	1
Clostridium tetani E88	1	0.33	1	1	1	1	1	0	0.2	0.5	1	0.4	0.5	0.2	0.86	0.4	0	0.33	1	0.67	1	0	0.33	0.25
Clostridium tetanomorphum DSM 665	1	1	1	1	1	1	1	0	1	0	1	1	0.5	1	1	0.6	0.8	0.33	1	0.67	1	1	0.33	1
Clostridium thermocellum ATCC 27405	1	1	1	1	1	1	1	1	1	0.5	1	0.8	0	1	1	0.8	0.8	0.67	1	1	0.5	0.83	0.67	1
Clostridium thermocellum BC1	1	1	1	1	1	1	1	1	1	1	1	0.8	0.5	1	1	0.6	0.8	0.67	1	0.33	0.5	1	0.67	1
Clostridium thermocellum DSM 2360	1	1	1	1	1	1	1	1	1	1	1	0.8	0.5	1	1	0.2	0.8	0.67	1	0.33	0.5	1	0.67	1
Clostridium thermocellum JW20	1	1	1	1	1	1	1	1	1	1	1	0.8	0.5	1	1	0.2	0.8	0.67	1	0.33	0.5	1	0.67	1
Clostridium tunisiense TJ	1	0.22	1	1	1	1	1	0	0.2	0	1	1	0	0.2	1	0.4	0	0.33	0.33	0	0	0	0	1
Clostridium tyrobutyricum DSM 2637Â	1	0.89	1	1	1	1	0	1	0.8	1	1	1	0.5	1	1	0.6	0.6	0.67	1	0	1	1	1	1
Clostridium tyrobutyricum DSM 2637 ATCC 25755	1	1	1	1	1	1	0	1	0.8	1	1	1	0.5	1	1	0.6	1	1	1	0.67	1	1	0.67	1
Clostridium tyrobutyricum DSM 2637	1	0.89	1	1	1	1	0	1	0.8	1	1	1	0.5	1	0.86	0.4	0.6	0.67	1	0	1	1	1	1
Clostridium tyrobutyricum UC7086	1	0.89	1	1	1	1	0	1	0.8	1	1	1	0.5	1	1	0.6	0.6	0.67	1	0	1	1	1	1
Clostridium ultunense DSM 10521	1	0.11	1	1	1	0	0	1	0.1	0	1	0.6	0.5	0.8	0.71	0.2	0.2	0.33	0	0.33	1	0	0.33	0.5
Cupriavidus basilensis OR16	1	0.89	0.33	1	1	1	1	1	0.8	1	1	1	0.5	1	0.89	0.4	0.8	1	1	1	1	0.83	1	1
Cupriavidus metallidurans CH34	1	1	0.33	1	1	1	1	1	0.9	1	1	1	0.5	1	1	0.8	1	1	1	1	1	1	1	1
Cupriavidus metallidurans tb_SCADC008	1	1	0.67	1	1	1	0	1	0.7	1	1	1	0.5	1	1	0.4	1	1	1	1	1	1	1	1
Cupriavidus necator N-1	1	1	1	1	1	1	1	1	0.9	1	1	1	0.5	1	1	0.8	1	1	1	1	1	1	1	1
Cupriavidus scadc_MAG058	1	0.89	0.33	0	1	1	1	1	0.7	1	0.67	1	0	1	0.86	0.2	0.8	1	0.67	0.67	0.5	1	1	1
Cupriavidus sp. amp6	1	1	0.33	0	1	1	1	1	0.7	1	1	1	0.5	1	1	0.4	1	1	1	1	1	1	1	1
Cupriavidus sp. BIS7	1	0.89	1	1	1	1	1	1	0.9	1	1	1	0	1	0.89	0.4	1	1	1	1	1	1	1	1
Cupriavidus sp. HMR-1	1	1	0.33	1	1	1	1	1	0.7	1	1	1	0.5	1	1	0.4	1	1	1	1	1	1	1	1
Cupriavidus sp. HPC(L)	1	1	1	0	1	1	1	1	0.7	1	1	1	0	1	1	0.4	1	1	1	1	1	1	1	1
Cupriavidus sp. UYMMa02A	1	0.89	0.33	1	0.5	1	1	1	0.9	1	1	0.8	0	0.8	0.86	0.6	1	1	0.33	0.33	0	0.67	1	0.75
Cupriavidus sp. UYPR2.512	1	1	1	0	1	1	1	1	0.7	1	1	1	0.5	1	1	0.4	1	1	1	1	1	1	1	1
Cupriavidus sp. WS	1	1	0.67	1	1	1	1	1	0.8	1	1	1	0.5	1	1	0.4	1	1	1	1	1	1	1	1
Cupriavidus taiwanensis LMG19424	1	0.89	0	1	1	1	1	1	1	1	1	1	0	1	0.89	0.6	1	1	1	1	1	1	1	1
Dehalobacter sp. FTH1	1	1	1	1	1	1	1	1	1	1	1	1	1	1	1	0.4	0.8	1	1	1	1	1	1	1
Dehalobacter sp. UNSWDHB	1	1	1	1	1	1	1	1	1	0.5	1	0.8	1	1	0.86	0.4	0.8	1	1	0.67	1	1	0.67	1
Dehalococcoidales scadc_MAG083.x	0.33	1	0.33	0	1	1	1	1	0.8	0	1	0.8	0.5	1	1	0	0.8	1	1	0.33	0.5	0.17	0.67	1
Dehalococcoides ethenogenes 195.x	1	1	0.33	1	1	1	1	1	0.8	0	1	0.8	0.5	1	1	0	0.8	1	1	0.33	0.5	1	0.67	1
Dehalococcoides mccartyi BTF08.x	0	1	0.33	1	1	1	1	1	0.8	0	1	0.8	0.5	1	1	0	0.8	0.67	1	0.33	0.5	1	0.67	0.75
Dehalococcoides mccartyi DCMB5.x	1	1	0.33	1	1	1	1	1	0.9	0	1	0.8	0.5	1	1	0	0.8	0.67	1	0.33	0.5	1	0.67	0.75
Dehalococcoides scadc_MAG133.x	1	0.89	1	0	1	1	0	1	0.7	0	1	0.8	0	1	1	0.4	0.8	1	1	0.33	0.5	1	0.67	1
Dehalococcoides sp. BAV1.x	0.33	1	0.33	1	1	1	1	1	0.9	0	1	0.8	0.5	1	1	0	0.8	0.67	1	0.67	0.5	1	0.67	1
Dehalococcoides sp. CBDB1.x	1	1	0.33	1	1	1	1	1	0.8	0	1	0.8	0.5	1	1	0	0.8	1	1	0.33	0.5	1	0.67	1
Dehalococcoides sp. GT.x	0.33	1	0.67	1	1	1	1	1	0.7	0	1	0.8	0.5	1	1	0	1	0.33	1	0.33	0.5	1	0.33	1
Dehalococcoides sp. VS.x	1	1	0.33	1	1	1	1	1	0.8	0	1	0.8	0.5	1	1	0	0.8	1	1	0.33	0.5	1	0.67	1
Dehalogenimonas lykanthroporepellens BL-DC-9	1	1	0.67	1	1	1	1	1	0.7	0.5	1	0.8	0.5	1	1	0	1	0.33	1	0.33	1	1	0.33	1
Dehalogenimonas lykanthroporepellens tb_SCADC011	1	1	1	0	1	1	1	1	0.9	0	1	0.8	0.5	1	1	0.4	0.8	1	1	0.33	0.5	1	0.67	1
Desulfitobacterium dehalogenans ATCC 51507	1	1	1	1	1	1	0	1	1	0.5	1	1	0	1	1	0.8	0.8	0.67	1	0.67	1	1	0.67	1
Desulfitobacterium dichloroeliminans LMG P-21439	1	1	1	1	1	1	0	1	1	0.5	1	1	0	1	1	0.8	0.8	0.67	1	0.67	1	0.17	0.67	1

Desulfitobacterium hafniense DCB-2	1	1	1	1	1	1	0	1	1	0.5	1	1	0.5	1	1	0.6	1	0.67	1	0.67	1	0.83	0.67	0.75
Desulfitobacterium hafniense DP7	1	1	1	1	1	1	0	1	1	1	1	1	1	1	0.86	0.2	0.8	0.67	0.67	0.33	1	1	0.67	1
Desulfitobacterium hafniense PCP-1	1	1	1	1	1	1	0	1	1	1	1	1	1	1	0.86	0.2	0.8	0.67	0.67	0.33	1	1	0.67	1
Desulfitobacterium hafniense TCP-A	1	1	1	1	1	1	0	1	1	1	1	1	1	1	0.86	0.2	0.8	0.67	1	0.33	1	1	0.67	1
Desulfitobacterium hafniense Y51	0.33	0.33	0.33	1	0	1	0	1	0.5	0	0.67	0.8	0	1	0.43	0.2	0.2	0	0.33	0.33	0.5	0.67	0	0.75
Desulfitobacterium sp. PCE1	1	1	1	1	1	1	0	1	1	1	1	1	1	1	0.86	0.4	0.8	1	1	0.33	1	1	0.67	1
Desulfobacca acetoxidans DSM 11109	1	1	1	1	1	1	0	1	0.9	0	1	1	0.5	1	1	0	1	1	1	0.33	0.5	1	0.67	1
Desulfobacter postgatei 2ac9	1	1	1	1	0.5	1	0	1	0.8	0	1	0.8	0.5	1	1	0.2	0.8	0.67	1	1	0.5	1	0.67	1
Desulfobacteraceae scadc_MAG073	1	1	1	1	1	1	0	1	0.9	0	1	0.8	0.5	1	0.86	0	0.8	0.67	1	1	0	1	0.67	1
Desulfobacterium autotrophicum HRM2	1	1	1	1	1	1	0	1	0.9	0.5	1	0.8	0.5	1	0.86	0.4	1	0.67	1	1	0.5	1	0.67	1
Desulfobacula toluolica Tol2	1	1	1	1	1	1	0	1	0.8	0	1	0.8	0.5	1	1	0.2	0.8	1	1	1	0.5	1	0.67	1
Desulfobulbus propionicus DSM 2032	1	1	1	1	1	1	0	1	0.8	1	1	0.8	0	1	1	0.2	0.8	0.67	1	1	0.5	1	0.33	1
Desulfobulbus propionicus tb_SCADC006	1	1	1	1	1	1	0	1	0.9	1	1	0.8	0.5	1	1	0.2	0.8	0.67	1	0.67	0.5	0.67	0.33	1
Desulfomicrobium baculatum DSM 4028	1	1	1	1	1	1	0	1	0.8	1	1	0.8	0.5	1	1	0.4	1	0.33	1	0.33	0.5	1	0.33	1
Desulfomicrobium baculatum tb_SCADC001	0.33	0.56	1	0	0	1	0	1	0.4	0	0.33	0.6	0	0.4	0.57	0.2	0.2	0	0.67	0	0	0.17	0	0.75
Desulfosporosinus acidiphilus SJ4	1	1	1	1	1	1	0	1	1	0.5	1	0.8	0	1	1	0.6	0.8	0.67	1	0.67	1	0.83	0.67	1
Desulfosporosinus meridiei DSM 13257 PRJNA224116	1	1	1	1	1	1	0	1	1	0.5	1	1	0	1	1	0.8	0.8	0.67	1	0.67	1	1	0.67	1
Desulfosporosinus orientis DSM 765	1	1	1	1	1	1	0	1	0.9	0.5	1	1	0	1	1	0.4	1	0.67	1	0.67	1	1	0.67	1
Desulfosporosinus sp. OT	1	1	1	1	1	1	0	1	1	0	1	1	1	1	1	0.4	0.8	1	1	0.33	1	1	0.67	1
Desulfosporosinus youngiae DSM 17734	1	0.78	1	1	1	1	0	1	1	0.5	1	1	0.5	1	1	0.6	0.6	1	1	0.67	1	1	0.67	1
Desulfotomaculum acetoxidans DSM 771 PRJNA224116	1	1	1	1	1	1	0	1	1	0	1	0.8	0.5	1	1	0.2	1	0.33	1	0.67	1	1	0.33	1
Desulfotomaculum alcoholivorax DSM 16058	1	1	1	1	1	1	0	1	0.9	0	1	1	0.5	1	1	0	0.8	1	1	0.67	1	0.67	0.67	1
Desulfotomaculum carboxydivorans CO-1-SRB	1	1	1	1	1	1	0	1	1	0	1	1	0.5	1	1	0.2	1	0.67	1	0.67	1	1	0.67	1
Desulfotomaculum gibsoniae DSM 7213	1	1	1	1	1	1	0	1	0.9	0	1	0.8	0	1	1	0.2	1	0.67	1	0.33	1	1	0.67	1
Desulfotomaculum hydrothermale Lam5 = DSM 18033	1	1	1	1	1	1	0	1	1	0	1	1	1	1	1	0	0.8	0.67	1	0.33	1	0.67	0.67	1
Desulfotomaculum hydrothermale Lam5(T)	1	1	1	1	1	1	0	1	1	0	0.67	1	0.5	0.8	0.57	0	0.8	0.67	1	0.33	1	1	0.67	1
Desulfotomaculum kuznetsovii DSM 6115	1	1	1	1	1	1	0	1	1	0	1	1	0.5	1	1	0.2	1	0.67	1	0.33	1	1	0.67	1
Desulfotomaculum reducens MI-1	1	0.89	1	1	1	1	0	1	1	0	1	1	0.5	1	1	0.4	0.8	0.67	1	0.33	1	1	0.67	1
Desulfotomaculum ruminis DSM 2154	1	0.89	1	1	1	1	1	1	0.9	0.5	1	1	0.5	1	1	0.6	0.8	0.67	1	0.33	1	1	0.67	1
Desulfovibrio aespoeensis Aspo-2	1	1	1	1	1	1	1	1	0.8	0.5	1	0.8	0.5	1	1	0.4	1	0.67	1	0.67	0.5	1	0.67	1
Desulfovibrio africanus PCS	1	1	1	1	1	1	1	1	0.8	0	1	1	0.5	1	1	0.4	0.8	1	1	0.33	0.5	1	0.67	1
Desulfovibrio africanus Walvis Bay	1	0.89	1	1	1	1	1	1	0.9	0	1	1	0.5	1	1	0.2	0.8	0.67	1	0.33	0.5	1	0.67	1
Desulfovibrio alkalitolerans DSM 16529	1	1	1	1	1	1	1	1	0.8	0.5	1	1	0.5	1	1	0.2	0.8	1	1	0.33	0.5	1	0.67	1
Desulfovibrio cf. magneticus IFRC170	1	1	1	1	1	1	1	1	0.8	1	1	1	0.5	1	1	0.4	0.8	1	1	0.33	0.5	0.67	0.67	1
Desulfovibrio desulfuricans ND132	1	0.89	1	1	1	1	1	1	0.8	1	1	0.8	0.5	1	1	0.2	0.8	0.67	1	0.67	1	1	0.67	1
Desulfovibrio desulfuricans subsp. aestuarii DSM 17919	1	1	0.33	1	1	1	1	1	0.9	0	1	0.8	0.5	1	1	0.4	0.8	1	1	0	0.5	0.83	0.67	1
Desulfovibrio desulfuricans subsp. desulfuricans ATCC 27774	1	1	1	1	1	1	1	1	0.9	0.5	1	1	0.5	1	1	0.4	1	0.67	1	0.33	0.5	1	0.67	1
Desulfovibrio desulfuricans subsp. desulfuricans DSM 642	1	1	1	1	1	1	1	1	0.9	0	1	1	0.5	1	1	0.4	0.8	0.67	1	0.33	0	0.67	0.33	1
Desulfovibrio desulfuricans subsp. desulfuricans G20	1	1	1	1	1	1	1	1	0.9	0	1	1	0.5	1	1	0.4	0.8	0.33	1	0.67	0.5	1	0.33	1
Desulfovibrio fructosivorans tb_SCADC002	1	0.33	1	1	0.5	1	1	1	0.5	0	0.33	0	0	0.6	0.57	0.2	0.4	0.33	0	0	0	0.5	0	0
Desulfovibrio fructosovorans JJ	1	1	1	1	1	1	1	1	0.9	1	1	0.8	0.5	1	0.86	0.4	0.8	1	1	0.33	0.5	1	0.67	1
Desulfovibrio gigas DSM 1382 = ATCC 19364	1	0.89	1	1	1	1	1	1	0.9	1	1	0.8	0	1	1	0.4	0.8	0.33	1	0.33	1	0.83	0.33	1
Desulfovibrio hydrothermalis AM13 = DSM 14728	1	1	1	1	1	1	1	1	0.8	0	1	1	0.5	1	1	0.6	0.8	1	1	0.33	0.5	1	0.67	1
Desulfovibrio inopinatus DSM 10711	1	1	1	1	1	1	1	1	0.8	0.5	1	1	0.5	1	1	0.4	0.8	1	1	0.33	0.5	1	0.67	1
Desulfovibrio longus DSM 6739	1	1	1	1	1	1	1	1	0.8	0	1	1	0.5	1	1	0	1	1	1	0.33	0.5	0.83	0.67	1
Desulfovibrio magneticus Maddingley MBC34	1	1	1	1	1	1	1	1	0.8	1	1	1	0.5	1	1	0.4	0.8	1	1	0.33	0.5	0.67	0.33	1
Desulfovibrio magneticus RS-1	1	1	1	1	1	1	1	1	0.8	1	1	1	0.5	1	1	0.6	1	1	1	0.33	0.5	1	0.67	1
Desulfovibrio magneticus str. Maddingley MBC34	1	0.78	1	1	1	1	1	1	0.8	0.5	1	1	0.5	1	1	0.2	0.6	1	1	0.33	0.5	0.67	0.67	1
Desulfovibrio oxyclinae DSM 11498	1	1	1	1	1	1	1	1	0.8	0	1	0.8	0.5	1	1	0	0.8	0.67	1	0.33	0.5	1	0.67	0.75
Desulfovibrio piezophilus C1TLV30	1	1	1	1	1	1	1	1	0.9	1	1	1	0.5	1	1	0.4	0.8	1	1	0.67	0.5	1	0.67	1
Desulfovibrio piger ATCC 29098	1	1	0.33	1	1	1	1	1	0.8	0.5	1	1	0.5	1	1	0.2	0.8	0.33	1	0	0	0.67	0	1
Desulfovibrio putealis DSM 16056	1	1	1	1	1	1	1	1	0.8	1	1	1	0.5	1	1	0	0.8	1	1	0.33	0.5	1	0.67	1
Desulfovibrio salexigens DSM 2638	1	1	1	1	1	1	1	1	0.8	0	1	0.8	0.5	1	1	0.6	1	0.33	1	0.33	1	1	0.33	1
Desulfovibrio scadc_MAG034	1	0.89	1	1	1	1	1	1	0.8	0	1	1	0.5	1	0.86	0	0.8	1	1	0	0.5	0.83	0.67	1
Desulfovibrio sp. 3_1_syn3	1	1	1	1	1	1	1	1	0.9	0.5	1	1	0.5	1	1	0.6	0.8	0.67	1	0.33	0.5	1	0.67	1
Desulfovibrio sp. A2	1	1	1	1	1	1	1	1	0.9	0	0.67	1	0.5	1	0.86	0.4	0.8	1	1	0	0.5	0.5	0.67	1
Desulfovibrio sp. Dsv1	1	0.67	1	1	1	1	1	1	0.6	0.5	1	1	0.5	0.8	1	0.6	0.4	0.33	0.67	0	0	0.33	0	1
Desulfovibrio sp. FW1012B	1	1	1	1	1	1	1	1	0.9	1	1	1	0.5	1	1	0	0.8	1	1	0.33	0.5	1	0.67	1
Desulfovibrio sp. J2	1	1	0.33	1	1	1	1	1	0.9	1	1	0.8	0	1	0.71	0.4	1	0.67	1	0.67	0.5	1	0.67	1
Desulfovibrio sp. U5L	1	0.78	1	1	1	1	1	1	0.9	0.5	1	1	0.5	1	1	0.2	0.6	1	1	0.33	0.5	0.67	0.67	1
Desulfovibrio sp. X2	1	1	1	1	1	1	1	1	0.8	0.5	1	0.8	0.5	1	1	0.2	0.8	1	1	0.33	0.5	1	0.67	1
Desulfovibrio vulgaris DP4	1	1	1	1	1	1	1	1	0.9	0	1	0.8	0.5	1	1	0.4	0.8	0.67	1	0.33	0.5	1	0.67	1
Desulfovibrio vulgaris Hildenborough	1	1	1	1	1	1	1	1	0.9	0	1	1	0.5	1	1	0.4	1	0.67	1	0.33	0.5	1	0.67	1
Desulfovibrio vulgaris Miyazaki F	1	1	0	1	1	1	1	1	0.9	0	1	0.8	0	1	1	0.4	1	0.67	1	0.67	0.5	1	0.67	1
Desulfovibrio vulgaris RCH1	1	1	1	1	1	1	1	1	0.8	0	1	0.8	0.5	1	1	0.4	1	0.67	1	0.67	0.5	1	0.67	1
Elusimicrobia scadc_MAG144	1	1	0.33	1	1	1	1	1	0.6	0	1	0.8	0.5	1	1	0	0.8	1	0.67	0.33	0.5	1	0.67	1

Elusimicrobia sp AM014	1	1	0.33	1	1	1	1	1	0.8	1	0.67	0.4	0.5	0.8	0.86	0.4	0.8	0.67	1	0.33	0.5	1	0.67	0.5
Elusimicrobium minutum Pei191	1	0.22	0.67	1	1	1	1	1	0.9	0	1	0	0.5	0.4	1	0.2	0.2	0	1	0.67	1	0.17	0	0.25
Elusimicrobium scadc_MAG075	1	0.11	1	0	0.5	0	1	1	0	0	0.33	0	0	0	0.29	0	0	0.33	0	0.33	0.5	0	0.33	0.25
Geobacter bemidjiensis Bem	1	1	1	1	1	1	1	1	0.9	1	1	1	0	1	1	0.6	1	1	1	0.67	0.5	1	0.67	1
Geobacter daltonii FRC-32	1	1	1	1	1	1	1	1	0.9	1	1	1	0.5	1	1	0.4	1	0.67	1	0.67	0.5	1	0.67	1
Geobacter lovleyi SZ	1	1	1	1	1	1	1	1	0.9	1	1	1	0.5	1	1	0.6	1	0.67	1	0.67	0.5	1	0.67	1
Geobacter lovleyi tb_SCADC012	1	0.78	0.33	1	0.5	1	1	1	0.9	1	1	1	0.5	1	0.86	0.4	0.8	0.67	1	0	1	0.83	0.33	1
Geobacter metallireducens GS-15	1	1	1	1	1	1	1	1	1	1	1	1	0	1	1	0.4	0.8	1	1	0.67	0.5	1	0.67	1
Geobacter scadc_MAG023	1	0.67	0.33	1	1	1	1	1	0.9	1	1	1	0.5	1	1	0	0.6	0.67	1	0.33	0.5	1	0.33	1
Geobacter sp. M18	1	1	1	1	1	1	1	1	0.8	0.5	1	1	0.5	1	1	0.4	1	0.67	1	0.67	0.5	1	0.67	1
Geobacter sp. M21	1	1	1	1	1	1	1	1	0.9	1	1	1	0.5	1	1	0.2	1	0.67	1	0.33	0.5	1	0.67	1
Geobacter sulfurreducens KN400	1	1	1	1	1	1	1	1	1	1	1	1	0	1	1	0.4	0.8	1	1	0.67	0.5	1	0.67	1
Geobacter sulfurreducens PCA	1	1	1	1	1	1	1	1	1	1	1	1	0	1	1	0.4	0.8	1	1	0.67	0.5	1	0.67	1
Geobacter uraniireducens Rf4	1	1	1	1	1	1	1	1	1	1	1	1	0.5	1	1	0.4	1	0.67	1	0.33	0.5	1	0.67	1
Geobacteraceae scadc_MAG137	1	0.56	0	1	1	0	1	1	0.8	0.5	0.67	0.4	0	0.8	0.57	0	0.6	0.67	0.67	0.67	0.5	0.5	0.33	0.5
Mesotoga prima MesG1.Ag.4.2	1	0.22	1	1	1	1	1	1	0.1	0	0.67	0	0	0	1	0.4	0.2	0.67	1	0.33	1	0	0.67	0
Methanoculleus bourgensis MS2 type strain:MS2	1	1	1	1	0.5	1	1	1	0.9	0	1	0.8	0.5	0.6	0.86	0.4	1	1	0.33	0.67	0.5	1	0.67	1
Methanoculleus marisnigri JR1	1	0.89	1	1	1	1	1	1	0.9	0	1	0.8	0.5	1	1	0.4	0.8	0.67	0.33	0.67	0.5	1	0.67	1
Methanoculleus scadc_MAG019	1	0.56	1	1	1	1	1	1	0.6	0.5	1	0.2	0.5	0.8	0.71	0	0.2	0.33	0	0.67	0.5	1	0	0.25
Methanolinea tarda tb_SCADC013	1	0.89	1	1	0.5	1	0	1	0.9	0	1	0.8	0.5	0.8	1	0	1	1	0	0.67	0.5	1	1	0.75
Methanomassiliicoccus luminyensis B10	1	1	1	1	1	1	1	1	0.9	1	1	0.8	0.5	1	1	0.2	1	0.67	1	0.67	1	1	0.67	1
Methanomicrobiales scadc_MAG072	1	1	1	1	0	1	1	1	0.8	0	1	1	0.5	1	1	0	0.8	1	0	0.67	0.5	1	0.67	1
Methanoregulaceae sp TB045	1	0.44	1	1	0.5	1	0	0	0.4	0	0.67	0	0.5	0.4	0.43	0.8	0.8	0.33	0	0.33	0	0.5	0.33	0
Methanosaeta concilii GP-6	1	1	1	1	0.5	1	1	1	0.8	0	1	1	0.5	1	1	0	1	1	0.33	0.67	0.5	1	0.67	1
Methanosaeta scadc_MAG172	1	0.89	1	1	0	1	1	1	0.5	0	0.33	1	0.5	0.8	0.29	0.2	0.8	0.67	0	0.67	0.5	1	0.67	1
Methanosaeta thermophila PT	1	0.89	0.67	1	0	1	1	1	0.8	0	1	0.8	0.5	1	1	0.2	0.8	0.67	0	0.67	0.5	1	0.67	1
Methanosarcina acetivorans C2A	1	1	1	1	1	1	1	1	0.9	1	1	0.8	0.5	1	0.86	0.2	0.8	1	1	1	0.5	1	0.67	1
Methanosphaera stadtmanae DSM 3091	1	1	1	1	1	1	1	1	0.9	1	1	0.8	0.5	1	1	0.4	0.8	1	0	0.67	0.5	0.67	0.67	1
Methanospirillum hungatei JF-1	1	0.89	1	1	1	1	1	1	0.9	1	1	1	0.5	0.8	1	0.4	0.8	0.67	0.33	0.67	0.5	1	0.67	1
Dehalococcoidales scadc_MAG083.y	0.33	1	0.33	0	1	1	1	1	0.8	0	1	0.8	0.5	1	1	0	0.8	1	1	0.33	0.5	0.17	0.67	1
Dehalococcoides ethenogenes 195.y	1	1	0.33	1	1	1	1	1	0.8	0	1	0.8	0.5	1	1	0	0.8	1	1	0.33	0.5	1	0.67	1
Dehalococcoides mccartyi BTF08.y	0	1	0.33	1	1	1	1	1	0.8	0	1	0.8	0.5	1	1	0	0.8	0.67	1	0.33	0.5	1	0.67	0.75
Dehalococcoides mccartyi DCMB5.y	1	1	0.33	1	1	1	1	1	0.9	0	1	0.8	0.5	1	1	0	0.8	0.67	1	0.33	0.5	1	0.67	0.75
Dehalococcoides scadc_MAG133.y	1	0.89	1	0	1	1	0	1	0.7	0	1	0.8	0	1	1	0.4	0.8	1	1	0.33	0.5	1	0.67	1
Dehalococcoides sp. BAV1.y	0.33	1	0.33	1	1	1	1	1	0.9	0	1	0.8	0.5	1	1	0	0.8	0.67	1	0.67	0.5	1	0.67	1
Dehalococcoides sp. CBDB1.y	1	1	0.33	1	1	1	1	1	0.8	0	1	0.8	0.5	1	1	0	0.8	1	1	0.33	0.5	1	0.67	1
Dehalococcoides sp. GT.y	0.33	1	0.67	1	1	1	1	1	0.7	0	1	0.8	0.5	1	1	0	1	0.33	1	0.33	0.5	1	0.33	1
Dehalococcoides sp. VS.y	1	1	0.33	1	1	1	1	1	0.8	0	1	0.8	0.5	1	1	0	0.8	1	1	0.33	0.5	1	0.67	1
Paludibacter bin 2	0.33	0	0	0	0	1	0	1	0.1	0	0.33	0.4	0	0.2	0.57	0.2	0	0.33	0.67	0.33	0.5	0	0	0.25
Pelobacter carbinolicus DSM 2380	1	1	0	1	1	1	1	1	1	1	1	1	0	1	1	0.4	1	1	1	0.67	1	1	0.67	1
Pelobacter scadc_MAG203	1	0.67	0	1	1	1	1	1	0.5	0.5	0.67	0.4	0	0.2	0.57	0.2	0.6	0.67	0.33	0.33	0	1	0.33	0.5
Pelotomaculum thermopropionicum SI	1	1	1	1	1	1	1	1	1	1	1	1	0	1	1	0.2	0.8	1	1	0.67	1	1	0.67	1
Peptococcaceae bacterium RM	1	0.11	1	1	1	1	1	1	1	0.5	0.67	0.8	0.5	1	0.71	0.4	0	0.67	1	0.33	1	0.67	0.67	1
Peptococcaceae scadc_MAG079	1	1	1	1	1	1	1	1	0.8	1	0.67	1	1	1	0.71	0.4	0.8	1	1	0.33	1	1	0.33	1
Peptococcaceae scadc_MAG091	1	0.56	1	1	1	1	1	1	1	1	1	0.6	0	1	1	0.4	0.4	1	1	0.67	0.5	1	0.67	0.75
Peptococcaceae scadc_MAG099	1	1	1	1	1	1	1	1	0.8	0	1	0.8	1	1	0.86	0.4	0.8	0	0.67	0.33	1	1	0.33	1
Peptococcaceae scadc_MAG122	1	1	0	1	1	1	1	1	1	0.5	1	1	1	1	0.86	0.4	0.8	0.33	1	0.67	0.5	0.17	0.67	1
Peptococcaceae scadc_MAG158	1	1	1	1	1	1	1	1	0.9	0	1	1	0.5	1	1	0.4	0.8	0.67	1	0.33	1	1	0.67	1
Peptococcaceae scadc_MAG171	1	0.89	0	0	0	1	1	0	0.9	0	0.67	0.4	0.5	0.8	0.43	0	0.6	0.67	0.33	0.33	0	0.83	0.67	0.5
Sphaerochaeta globosa Buddy	1	1	1	1	1	1	1	1	1	0	1	0.8	0	1	0.86	0.6	1	0.67	1	1	0.5	0.17	0.67	1
Sphaerochaeta pleomorpha Grapes	1	1	1	1	1	1	1	1	0.8	0.5	1	0.8	0	1	0.86	0.4	1	0.67	1	0.67	0.5	1	0.67	1
Syntrophomonas wolfei subsp. wolfei Goettingen	1	0.78	1	1	1	1	1	1	1	0	1	0.8	1	1	0.71	0.4	0.8	0.67	1	0.67	1	1	0.67	1
Syntrophus aciditrophicus SB	1	1	1	1	1	1	1	1	0.8	0.5	1	0.8	1	1	1	0.2	0.8	0.33	1	1	0.5	1	0.33	1
Syntrophus aciditrophicus scadc_MAG006	1	1	1	1	1	1	1	1	0.8	1	1	0.8	0.5	0.8	1	0.2	0.8	0.33	1	1	0.5	1	0.33	1
Syntrophus aciditrophicus tb_SCADC009	1	1	1	1	1	1	1	1	0.8	1	1	1	0.5	1	1	0.6	0.8	1	1	1	0.5	1	0.67	1
Syntrophus scadc_MAG275	1	0.56	1	1	1	1	0	1	0.5	0.5	0.33	1	0.5	0.8	0.14	0	0.6	0	0.33	1	0	0	0.33	1
Thermanaerovibrio acidaminovorans DSM 6589	1	0.89	0.67	1	1	1	1	1	0.8	1	1	0.8	0.5	1	0.89	0.2	0.8	0.33	0.67	0	1	0.17	0.33	1
Thermomicrobium roseum DSM 5159	1	0.78	0.33	1	0.5	1	1	1	1	1	1	1	0.5	1	0.71	0.2	0.6	1	0.33	0.33	1	1	0.67	1
Thiobacillus denitrificans ATCC 25259	1	1	1	0	1	1	1	1	1	0	1	1	0	1	1	0.8	0.8	0.67	1	1	0.5	1	0.67	1
Myxococcus fulvus HW-1	1	0.78	1	1	1	1	0	1	1	0.5	1	0.6	0.5	0.2	0.89	0	0.8	0.33	0.33	1	1	1	1	0.5
Myxococcus stipitatus DSM 14675	1	0.78	1	1	1	1	0	1	1	0.5	1	0.6	0.5	0.2	0.89	0	0.8	0.67	0.33	1	1	1	1	0.5
Myxococcus xanthus DK 1622	1	0.78	1	1	1	1	0	1	1	0.5	1	0.6	0.5	1	0.89	0	0.8	1	0.33	1	1	1	1	0.5
