# Supplementary material for: Vitamin and Amino Acid Auxotrophy in Anaerobic Consortia Operating under Methanogenic Conditions
Source: mSystems. 2017 Oct 31;2(5):e00038-17. doi: 10.1128/mSystems.00038-17 (PMC5663940; doi:10.1128/mSystems.00038-17)
Supplement: TABLE S3 [file sys005172144st4.pdf]

	1,4-dihydroxy-2-naphthoate	5,6-dimethylbenzimidazole	6-hydroxymethyl-dihydropterin diphosphate	7-keto-8-aminopelargonate	adenosylcobalamin	adenosylcobalamin salvage from cobalamin	adenosylcobalamin salvage from cobinamide	biotin	coenzyme A	coenzyme M
Agrobacterium albertimagni AOL15	0.14	0.67	0.6	0.73	0.71	1	0.83	0	1	0
Agrobacterium arsenijevicii KFB 330	0.29	1	0.6	0.82	0.81	1	0.83	1	1	0
Agrobacterium radiobacter DSM 30147	0.14	0	0.4	0.64	0.62	1	0.5	1	1	0
Agrobacterium radiobacter K84	0.14	0.67	0.6	0.64	0.57	1	0.5	0.67	0.5	0
Agrobacterium rhizogenes K599	0.43	0.67	0.6	0.64	0.67	1	0.67	1	0.5	0
Agrobacterium scadc_MAG084	0.43	1	0.2	0.64	0.81	1	0.83	0.67	1	0
Agrobacterium sp. 10MFCol1.1	0.29	1	0.6	0.82	0.81	1	0.83	1	1	0
Agrobacterium sp. 224MFTsu3.1	0.29	0.67	0.6	0.82	0.71	1	0.83	1	1	0
Agrobacterium sp. 33MFTa1.1	0.43	1	0.6	0.82	0.76	1	0.83	1	1	0
Agrobacterium sp. H13-3	0.43	0.67	0.6	0.82	1	1	1	1	1	0.14
Agrobacterium sp. KFB 330	0.29	1	0.6	0.82	0.81	1	0.83	1	1	0
Agrobacterium sp. LC34	0.29	1	0.6	0.82	0.76	1	0.83	1	1	0
Agrobacterium sp. R89-1	0.14	1	0.6	0.73	0.76	1	0.83	0.67	1	0
Agrobacterium sp. SUL3	0.29	1	0.6	0.82	0.76	1	0.83	1	1	0
Agrobacterium sp. UNC420CL41Cvi	0.29	1	0.6	0.82	0.76	1	0.83	1	1	0
Agrobacterium tumefaciens 5A v2	0.29	0.67	0.6	0.82	0.76	1	0.83	1	1	0
Agrobacterium tumefaciens Ach5	0.43	0.67	0.6	0.55	0.67	1	0.5	1	1	0
Agrobacterium tumefaciens C58	0.29	0.67	0.6	0.82	0.76	1	0.83	1	1	0
Agrobacterium tumefaciens F2	0.43	0.67	0.6	0.64	0.71	1	0.83	1	1	0
Agrobacterium tumefaciens GW4	0.29	0.67	0.6	0.82	0.71	1	0.83	1	1	0
Agrobacterium tumefaciens LBA4404	0.14	1	0.6	0.82	0.76	1	0.83	1	1	0
Agrobacterium tumefaciens S2	0.29	1	0.6	0.82	0.81	1	0.83	1	1	0
Agrobacterium tumefaciens S33	0.29	1	0.6	0.64	0.81	1	0.67	1	0.75	0
Agrobacterium tumefaciens WRT31	0.29	1	0.6	0.82	0.81	1	0.83	1	1	0.14
Agrobacterium vitis NCPPB 3554	0.14	1	0.6	0.82	0.76	1	0.83	0.67	1	0.14
Agrobacterium vitis S4	0.14	0.67	0.6	0.73	0.63	1	0.83	0.67	0.5	0.14
Clostridiales scadc_MAG157	0	0	0	0.27	0.63	1	0	0	1	0
Clostridiales scadc_MAG196	0	0	0	0.18	0.63	0	0	0	0.25	0
Clostridiales scadc_MAG262	0	0	0.2	0.5	0	0	0	0	0.25	0
Clostridium acetobutylicum ATCC 824	0	0	0.6	0.55	0.63	0	0	1	1	0
Clostridium acetobutylicum DSM 1731	0	0	0.6	0.55	0.67	0	0	1	1	0
Clostridium acetobutylicum EA 2018	0	0	0.6	0.45	0.67	0	0	1	1	0
Clostridium acidurici 9a	0	0	0.6	1	0.76	0	0	1	1	0
Clostridium autoethanogenum DSM 10061	0	0	0.6	0.36	0.75	1	0	0.33	0.75	0
Clostridium bartlettii CAG 1329	0	0	0.6	0.27	0.63	1	0	0	1	0
Clostridium beijerinckii G117	0	0	0.6	0.36	0.38	0	0	0.33	1	0
Clostridium beijerinckii NCIMB 8052	0.14	0	0.6	0.55	0.57	1	0	0.33	1	0
Clostridium beijerinckii NRRL B-598	0.14	0	0.6	0.36	0.62	1	0	0.33	1	0
Clostridium bifermentans ATCC 19299	0	0	0.6	0.27	0.57	1	0	0.67	1	0
Clostridium bifermentans ATCC 638	0	0	0.6	1	0.57	1	0	0.67	1	0
Clostridium botulinum A ATCC 19397	0.14	0	0.4	0.55	0.75	1	0	0.67	1	0
Clostridium botulinum A ATCC 3502	0.14	0	0.4	0.55	0.62	1	0	0.67	1	0
Clostridium botulinum A Hall	0.14	0	0.4	0.55	0.75	1	0	0.67	1	0
Clostridium botulinum B1 Okra	0.14	0	0.4	0.55	0.75	1	0	0.67	1	0
Clostridium butyricum 5521	0	0	0.6	0.27	0.56	1	0	1	1	0
Clostridium butyricum 60E.3	0	0	0.6	0.27	0.56	0	0	1	1	0
Clostridium butyricum DKU-01	0	0	0.6	0.27	0.56	0	0	1	1	0
Clostridium butyricum E4 BoNT E BL5262	0	0	0.6	0.27	0.56	1	0	1	1	0
Clostridium carboxidivorans P7	0	0	0.6	0.27	0.63	1	0	0.33	1	0
Clostridium carboxidivorans P7 PRJNA29495	0	0	0.6	0.27	0.38	1	0	0	0.75	0
Clostridium celatum DSM 1785	0	0	0.6	0.27	0.5	0	0	0	1	0
Clostridium cellulolyticum H10; ATCC 35319	0.14	0	0.6	0.55	0.62	1	0	0.33	1	0
Clostridium cellulovorans 743B	0.14	0	0.6	0.36	0.75	1	0	1	0.75	0
Clostridium cellulovorans 743B PRJNA52819	0	0	0.6	0.36	0.63	0	0	0.67	1	0
Clostridium citroniae WAL-17108	0	0	0.2	0.27	0.38	0	0	0	1	0
Clostridium difficile 630	0	0	0.6	0.45	0.63	1	0	0.33	1	0
Clostridium difficile CD196	0	0	0.6	0.45	0.71	1	0.83	0.33	0.5	0
Clostridium difficile MID11 7032989	0	0	0.6	0.45	0.63	1	0.83	0.33	0.5	0
Clostridium difficile MID12 7032985	0	0	0.6	0.45	0.63	1	0.83	0.33	1	0
Clostridium difficile MID13 7032994	0	0	0.6	0.27	0.67	1	0.83	0.33	1	0
Clostridium difficile R20291	0	0	0.6	0.45	0.76	1	0.83	0.33	0.5	0
Clostridium hathewayi CAG:224	0	0	0.2	0.27	0.5	1	0.5	0	0.75	0
Clostridium hiranonis DSM 13275	0	0	0.6	0.27	0.62	0	0.5	0	1	0
Clostridium hylemonae DSM 15053	0	0	0.2	0.27	0.52	0	0.5	0	1	0
Clostridium kluyveri DSM 555	0.14	0	0.6	0.55	0.75	1	1	1	1	0

Clostridium kluyveri NBRC 12016	0	0	0.2	0.36	0.5	0	0.67	0.67	1	0
Clostridium lentocellum DSM 5427	0.14	0	0	0.27	0.63	1	0.67	0.33	0.75	0
Clostridium leptum CAG 27	0	0	0.4	0.45	0.13	0	0.17	0	0.5	0
Clostridium leptum DSM 753	0	0	0.4	0.45	0.13	0	0.17	0	0.5	0
Clostridium ljungdahlii ATCC 49587	0.14	0	0.6	0.45	0.88	1	1	0.33	1	0
Clostridium nexile CAG:348	0	0	0.2	0.27	0	0	0	0	1	0
Clostridium novyi NT	0	0	0.6	0.55	0.63	1	0.83	1	1	0
Clostridium papyrosolvens C7	0	0	0.6	0.55	0.5	0	0.67	0.33	1	0
Clostridium pasteurianum BC1	0.86	0	0.6	0.5	0.57	1	0.5	1	1	0
Clostridium pasteurianum DSM 525	0.86	0	0.6	0.27	0.75	1	1	0.67	1	0
Clostridium perfringens ATCC 13124	0	0	0.6	0.45	0.57	1	0.5	0.67	1	0
Clostridium perfringens SM101	0	0	0.6	0.45	0.62	1	0.5	0.33	1	0
Clostridium perfringens str. 13	0	0	0.6	0.45	0.62	1	0.5	0.67	1	0
Clostridium phytofermentans ISDg	0	0	0.2	0.27	0.62	1	0.5	0.33	0.5	0
Clostridium saccharobutylicum DSM 13864	0.14	0	0.6	0.55	0.62	1	0.5	1	1	0
Clostridium saccharolyticum WM1	0.14	0	0	0.27	0.57	1	0.5	0	0.75	0
Clostridium saccharoperbutylacetonicum N1-4(HMT)	0.14	0	0.4	0.55	0.71	1	1	1	1	0
Clostridium saccharoperbutylacetonicum N1-4(HMT) ATCC 27021	0	0	0.6	0.36	0.71	1	1	0.67	1	0
Clostridium scadc_MAG005	0.14	0	0.6	0.27	0.67	0	0.83	0	1	0
Clostridium sp. ASF356	0	0	0	0.27	0.75	0	1	0	1	0
Clostridium sp. ASF502	0	0	0.6	0.27	0.13	1	0.17	0	0.5	0
Clostridium sporogenes ATCC 15579	0	0	0.4	0.27	0.63	1	0.83	0.67	1	0
Clostridium sporogenes PA 3679	0	0	0.4	0.27	0.63	1	0.83	0.67	1	0
Clostridium sticklandii DSM 519	0	0	0.6	0.45	0.63	1	0.83	0	1	0
Clostridium symbiosum ATCC 14940	0.14	0	0	0.27	0.38	1	0.5	0.67	0.5	0
Clostridium termitidis CT1112 CT1112	0.14	0	0.6	0.36	0.63	1	0.83	0	1	0
Clostridium tetani E88	0	0	0	0.55	0.67	1	0.83	0.67	1	0
Clostridium tetanomorphum DSM 665	0	0	0.6	0.45	0.75	1	1	1	1	0
Clostridium thermocellum ATCC 27405	0.14	0	0.6	0.64	0.75	1	1	1	1	0
Clostridium thermocellum BC1	0	0	0.6	0.64	0.63	1	1	0.67	1	0
Clostridium thermocellum DSM 2360	0	0	0.6	0.64	0.63	1	1	0.67	0.75	0
Clostridium thermocellum JW20	0	0	0.6	0.64	0.63	1	1	0.67	1	0
Clostridium tunisiense TJ	0	0	0.2	0.27	0.5	0	0.67	0.67	1	0
Clostridium tyrobutyricum DSM 2637Â	0	0	0.4	0.27	0.75	1	1	0.67	1	0
Clostridium tyrobutyricum DSM 2637 ATCC 25755	0	0	0.4	0.36	0.76	1	0.83	1	1	0
Clostridium tyrobutyricum DSM 2637	0	0	0.4	0.27	0.75	1	1	0.67	1	0
Clostridium tyrobutyricum UC7086	0	0	0.4	0.27	0.75	1	1	0.67	1	0
Clostridium ultunense DSM 10521	0.14	0	0.2	0.55	0.63	1	0.83	0	1	0
Cupriavidus basilensis OR16	0.14	0.67	0.8	1	0.33	1	0.33	1	1	0
Cupriavidus metallidurans CH34	0.29	0.67	0.6	1	1	1	1	1	1	0.14
Cupriavidus metallidurans tb_SCADC008	0.29	1	0.8	1	0.67	1	0.67	1	1	0
Cupriavidus necator N-1	0.29	0.67	0.8	1	1	1	0.83	1	1	0.29
Cupriavidus scadc_MAG058	0.29	1	0.6	1	0.67	1	0.67	1	1	0
Cupriavidus sp. amp6	0.43	0.67	0.8	1	0.75	1	0.83	1	1	0
Cupriavidus sp. BIS7	0.29	0.33	0.8	0.82	1	1	1	1	1	0
Cupriavidus sp. HMR-1	0.14	1	0.8	1	0.67	1	0.67	1	1	0
Cupriavidus sp. HPC(L)	0.14	0.67	0.6	1	0.44	1	0.5	1	1	0
Cupriavidus sp. UYMMa02A	0.43	0.67	0.8	0.64	0.38	1	0.33	0.33	0.25	0
Cupriavidus sp. UYPR2.512	0.43	0.67	0.8	0.91	0.75	1	1	1	1	0.14
Cupriavidus sp. WS	0.29	0.67	0.8	0.82	0.56	1	0.5	1	1	0
Cupriavidus taiwanensis LMG19424	0.43	0.67	0.8	0.64	0.88	1	1	1	1	0.14
Dehalobacter sp. FTH1	0.86	0	0.6	0	0.56	1	0.5	0	1	0
Dehalobacter sp. UNSWDHB	0.86	0	0.6	0	0.75	1	1	0	1	0
Dehalococcoidales scadc_MAG083.x	0	0	0.4	0	0.5	1	0.83	0	1	0
Dehalococcoides ethenogenes 195.x	0.14	0	0.4	0	0.75	1	1	0	1	0
Dehalococcoides mccartyi BTF08.x	0	0	0.4	0	0.88	1	1	0	1	0
Dehalococcoides mccartyi DCMB5.x	0	0	0.4	0	0.88	1	1	0	1	0
Dehalococcoides scadc_MAG133.x	0.14	0	0.4	0	0.56	1	0.83	0	1	0
Dehalococcoides sp. BAV1.x	0.14	0	0.4	0	0.63	1	0.67	0	1	0
Dehalococcoides sp. CBDB1.x	0.14	0	0.4	0	0.67	1	0.83	0	1	0
Dehalococcoides sp. GT.x	0.14	0	0.4	0	0.75	1	0.83	0	0.75	0
Dehalococcoides sp. VS.x	0.14	0	0.4	0	0.88	1	1	0	1	0
Dehalogenimonas lykanthroporepellens BL-DC-9	0.14	0	0.4	0	0.63	1	0.67	0	0.75	0
Dehalogenimonas lykanthroporepellens tb_SCADC011	0.14	0	0.6	0	0.56	1	0.83	0.33	1	0
Desulfitobacterium dehalogenans ATCC 51507	0.86	0	0.6	0.27	0.88	1	1	0	1	0
Desulfitobacterium dichloroeliminans LMG P-21439	0.14	0	0.6	0.27	0.63	1	0.67	0.67	1	0

Desulfitobacterium hafniense DCB-2	0.71	0	0.6	0.45	0.67	1	0.83	0	1	0
Desulfitobacterium hafniense DP7	0.71	0	0.6	0.27	0.75	1	1	0	1	0.14
Desulfitobacterium hafniense PCP-1	0.57	0	0.6	0.27	0.75	1	1	0	1	0.14
Desulfitobacterium hafniense TCP-A	0.57	0	0.6	0.27	0.67	1	0.83	0	1	0.14
Desulfitobacterium hafniense Y51	0.71	0	0	0.27	0.75	1	1	0	0.25	0
Desulfitobacterium sp. PCE1	0.86	0	0.6	0.27	0.75	1	1	0.33	1	0
Desulfobacca acetoxidans DSM 11109	0	0	0.4	0.5	0.75	1	0.83	1	0.75	0
Desulfobacter postgatei 2ac9	0.43	0	0.4	0.36	0.63	1	0.83	0	1	0
Desulfobacteraceae scadc_MAG073	0	0	0.4	0.73	0.63	1	0.83	0	1	0
Desulfobacterium autotrophicum HRM2	0.14	0	0.2	0.45	0.75	1	0.83	0.33	1	0.14
Desulfobacula toluolica Tol2	0.29	0	0.4	0.64	0.63	1	0.83	1	1	0
Desulfobulbus propionicus DSM 2032	0.14	0	0.4	0.5	0.75	1	0.83	1	1	0
Desulfobulbus propionicus tb_SCADC006	0	0	0.4	0.73	0.52	1	0.83	1	1	0
Desulfomicrobium baculatum DSM 4028	0.14	0	0.4	0.5	0.63	1	0.67	1	1	0
Desulfomicrobium baculatum tb_SCADC001	0	0	0.2	0.36	0.25	1	0.33	0	0.5	0
Desulfosporosinus acidiphilus SJ4	0.14	0	0.6	0.27	0.75	1	0.83	0.33	1	0
Desulfosporosinus meridiei DSM 13257 PRJNA224116	0.14	0	0.6	0.5	0.88	1	1	0.33	1	0.14
Desulfosporosinus orientis DSM 765	0.29	0	0.6	1	0.67	1	0.67	1	1	0.14
Desulfosporosinus sp. OT	0	0	0.6	0.5	0.63	1	0.83	0.67	0.5	0.14
Desulfosporosinus youngiae DSM 17734	0	0	0.6	0.64	0.75	1	1	0	1	0.14
Desulfotomaculum acetoxidans DSM 771 PRJNA224116	0.29	0	0.6	0.45	0.75	1	0.83	1	0.75	0
Desulfotomaculum alcoholivorax DSM 16058	0.29	0	0.6	0.5	0.63	1	0.83	1	1	0.14
Desulfotomaculum carboxydivorans CO-1-SRB	0	0	0.6	0.5	0.75	1	0.83	1	0.75	0.14
Desulfotomaculum gibsoniae DSM 7213	0.29	0	0.6	0.5	0.67	1	0.67	1	1	0.14
Desulfotomaculum hydrothermale Lam5 = DSM 18033	0.14	0	0.6	0.27	0.75	1	1	0.33	1	0.14
Desulfotomaculum hydrothermale Lam5(T)	0.14	0	0.6	0.45	0.75	1	1	0.33	1	0.14
Desulfotomaculum kuznetsovii DSM 6115	0.14	0	0.6	1	0.71	1	0.67	1	0.75	0.14
Desulfotomaculum reducens MI-1	0	0	0.6	0.45	0.75	1	0.83	0.33	1	0.14
Desulfotomaculum ruminis DSM 2154	0.14	0	0.6	0.27	0.75	1	0.83	0.33	1	0.14
Desulfovibrio aespoeensis Aspo-2	0.14	0	0.2	0.5	0.52	0	0.33	1	0.75	0.14
Desulfovibrio africanus PCS	0	0	0.4	0.82	0.52	0	0.67	0.33	1	0
Desulfovibrio africanus Walvis Bay	0.14	0	0.4	0.5	0.52	0	0.33	1	1	0.14
Desulfovibrio alkalitolerans DSM 16529	0	0	0.4	0.64	0.33	0	0.33	0.33	0.5	0
Desulfovibrio cf. magneticus IFRC170	0	0	0.4	0.82	0.48	0	0.67	1	0.5	0.14
Desulfovibrio desulfuricans ND132	0.14	0	0.4	0.36	0.52	0	0.33	0.33	0.75	0.14
Desulfovibrio desulfuricans subsp. aestuarii DSM 17919	0	0	0.4	0.73	0.52	0	0.5	1	0.75	0
Desulfovibrio desulfuricans subsp. desulfuricans ATCC 27774	0.29	0	0.4	1	0.67	1	0.67	1	0.75	0
Desulfovibrio desulfuricans subsp. desulfuricans DSM 642	0.14	0	0.4	0.91	0.63	1	0.83	0.67	1	0
Desulfovibrio desulfuricans subsp. desulfuricans G20	0.14	0	0.4	0.64	0.63	1	0.67	1	1	0
Desulfovibrio fructosivorans tb_SCADC002	0	0	0.2	0.64	0.33	0	0.17	0.33	0.25	0
Desulfovibrio fructosovorans JJ	0	0	0.4	0.73	0.43	0	0.33	1	0.5	0.14
Desulfovibrio gigas DSM 1382 = ATCC 19364	0	0	0.2	0.5	0.63	1	0.67	0.33	1	0
Desulfovibrio hydrothermalis AM13 = DSM 14728	0.14	0	0.4	0.82	0.52	0	0.5	1	1	0
Desulfovibrio inopinatus DSM 10711	0	0	0.4	0.91	0.25	0	0.33	0.67	0.5	0.14
Desulfovibrio longus DSM 6739	0	0	0.4	0.55	0.43	0	0.33	0.33	1	0.14
Desulfovibrio magneticus Maddingley MBC34	0	0	0.4	0.82	0.52	0	0.67	1	1	0.14
Desulfovibrio magneticus RS-1	0.14	0	0.4	0.82	0.62	0	0.67	1	1	0.14
Desulfovibrio magneticus str. Maddingley MBC34	0	0	0.4	0.82	0.38	0	0.67	1	0.5	0.14
Desulfovibrio oxyclinae DSM 11498	0	0	0.4	0.64	0.38	0	0.33	1	0.5	0.14
Desulfovibrio piezophilus C1TLV30	0	0	0.2	0.64	0.52	0	0.5	0.33	1	0.14
Desulfovibrio piger ATCC 29098	0	0	0.2	0.73	0.38	0	0.67	0.33	0.25	0
Desulfovibrio putealis DSM 16056	0	0	0.4	0.73	0.33	0	0.33	0.67	1	0
Desulfovibrio salexigens DSM 2638	0.14	0	0.4	0.5	0.52	0	0.33	1	0.75	0
Desulfovibrio scadc_MAG034	0	0	0.2	0.73	0.33	0	0.33	0.33	1	0.14
Desulfovibrio sp. 3_1_syn3	0.14	0	0.4	1	0.63	0	0.83	1	0.75	0
Desulfovibrio sp. A2	0	0	0.4	0.64	0.63	1	0.67	1	0.75	0
Desulfovibrio sp. Dsv1	0	0	0.4	0.55	0.5	0	0.67	0.67	0.5	0
Desulfovibrio sp. FW1012B	0	0	0.4	0.82	0.43	0	0.33	1	0.5	0.14
Desulfovibrio sp. J2	0.14	0	0.4	0.5	0.52	0	0.33	0.33	1	0.14
Desulfovibrio sp. U5L	0	0	0.4	0.73	0.38	0	0.67	1	0.5	0.14
Desulfovibrio sp. X2	0	0	0.4	0.73	0.38	0	0.33	1	0.5	0
Desulfovibrio vulgaris DP4	0	0	0.4	0.64	0.62	1	0.5	1	1	0
Desulfovibrio vulgaris Hildenborough	0	0	0.4	0.82	0.63	1	0.83	1	0.75	0
Desulfovibrio vulgaris Miyazaki F	0.14	0	0.4	0.64	0.62	1	0.5	1	1	0
Desulfovibrio vulgaris RCH1	0.14	0	0.4	0.5	0.63	1	0.67	1	0.75	0
Elusimicrobia scadc_MAG144	0	0	0.2	0.45	0.5	1	0.83	0	0.75	0

Elusimicrobia sp AM014	0.14	0	0.6	0.64	0.13	1	0.17	0.33	0.5	0
Elusimicrobium minutum Pei191	0.14	0	0.4	0.5	0.05	0	0	0	0.5	0
Elusimicrobium scadc_MAG075	0	0	0.2	0.5	0.05	0	0	0	0.5	0
Geobacter bemidjiensis Bem	0.29	0	0.4	1	0.88	1	1	1	1	0
Geobacter daltonii FRC-32	0.29	0	0.4	0.64	0.75	1	0.83	1	1	0
Geobacter lovleyi SZ	0.14	0	0.4	0.64	0.71	1	0.83	1	1	0
Geobacter lovleyi tb_SCADC012	0	0	0.4	0.91	0.63	1	1	1	1	0
Geobacter metallireducens GS-15	0.29	0	0.4	0.82	0.76	1	1	1	1	0
Geobacter scadc_MAG023	0.14	0	0.4	0.73	0.63	1	1	0.67	1	0
Geobacter sp. M18	0.14	0	0.4	0.64	0.63	1	0.67	1	0.75	0
Geobacter sp. M21	0.29	0	0.4	0.5	0.75	1	0.83	1	0.75	0
Geobacter sulfurreducens KN400	0.14	0	0.4	0.82	0.75	1	1	1	1	0
Geobacter sulfurreducens PCA	0.14	0	0.4	0.82	0.76	1	1	1	1	0
Geobacter uraniireducens Rf4	0.14	0	0.4	0.55	0.75	1	0.83	1	1	0
Geobacteraceae scadc_MAG137	0	0	0.4	0.36	0.25	0	0.33	0.67	0	0
Mesotoga prima MesG1.Ag.4.2	0	0	0.2	0	0.13	1	0	0	0.5	0.14
Methanoculleus bourgensis MS2 type strain:MS2	0	0	0	0	0.63	1	0	0	0.75	0
Methanoculleus marisnigri JR1	0	0	0.2	0	0.48	0	0	0	0.75	0
Methanoculleus scadc_MAG019	0	0	0.6	0	0.52	0	0	0	0.75	0
Methanolinea tarda tb_SCADC013	0	0	0.6	0	0.48	0	0	0	0.75	0
Methanomassiliicoccus luminyensis B10	0	0	0.6	0	0.57	1	0	0	0.75	0.14
Methanomicrobiales scadc_MAG072	0	0	0.6	0	0.38	0	0	0	0.75	0
Methanoregulaceae sp TB045	0	0	0	0	0.29	0	0	0	0.25	0
Methanosaeta concilii GP-6	0	0	0	0	0.57	0	0	0	0.5	0.14
Methanosaeta scadc_MAG172	0	0	0.4	0	0.43	0	0	0	0.25	0.14
Methanosaeta thermophila PT	0	0	0.2	0	0.52	0	0	0	0.75	0.14
Methanosarcina acetivorans C2A	0	0	0.2	0	0.57	1	0	0	0.75	0.14
Methanosphaera stadtmanae DSM 3091	0	0	0.2	0	0.43	0	0	0	0.75	0.43
Methanospirillum hungatei JF-1	0	0	0.2	0	0.52	1	0	0	0.75	0
Dehalococcoidales scadc_MAG083.y	0	0	0.4	0	0.5	1	0.83	0	1	0
Dehalococcoides ethenogenes 195.y	0.14	0	0.4	0.18	0.75	1	1	0	1	0
Dehalococcoides mccartyi BTF08.y	0	0	0.4	0.36	0.88	1	1	0	1	0
Dehalococcoides mccartyi DCMB5.y	0	0	0.4	0.36	0.88	1	1	0	1	0
Dehalococcoides scadc_MAG133.y	0.14	0	0.4	0.18	0.56	1	0.83	0	1	0
Dehalococcoides sp. BAV1.y	0.14	0	0.4	0.27	0.63	1	0.67	0	1	0
Dehalococcoides sp. CBDB1.y	0.14	0	0.4	0.36	0.67	1	0.83	0	1	0
Dehalococcoides sp. GT.y	0.14	0	0.4	0.27	0.75	1	0.83	0	0.75	0
Dehalococcoides sp. VS.y	0.14	0	0.4	0.18	0.88	1	1	0	1	0
Paludibacter bin 2	0	0	0.2	0.5	0	0	0	0	0	0
Pelobacter carbinolicus DSM 2380	0.14	0	0.4	1	0.75	1	1	1	1	0
Pelobacter scadc_MAG203	0	0	0.2	0.45	0.05	0	0	0	0.25	0
Pelotomaculum thermopropionicum SI	0.14	0	0.6	0.55	0.57	1	0.83	0.67	1	0
Peptococcaceae bacterium RM	0.86	0	0.6	0.55	0.13	0	0.17	0	1	0
Peptococcaceae scadc_MAG079	0	0	0.6	0.45	0.5	1	0.5	0	1	0
Peptococcaceae scadc_MAG091	0.14	0	0.6	0.64	0.13	1	0.17	0	1	0
Peptococcaceae scadc_MAG099	0	0	0.6	0.45	0.13	1	0.17	0	1	0
Peptococcaceae scadc_MAG122	0	0	0.4	0.45	0.25	1	0.33	0	1	0
Peptococcaceae scadc_MAG158	0.29	0	0.6	0.5	0.25	1	0.33	0.33	1	0
Peptococcaceae scadc_MAG171	0	0	0.6	0.55	0.38	0	0.67	0.33	0.75	0
Sphaerochaeta globosa Buddy	0	0	0	0.36	0.5	1	0.67	0	0.5	0
Sphaerochaeta pleomorpha Grapes	0.14	0	0.6	0.27	0.5	1	0.67	0	0.5	0
Syntrophomonas wolfei subsp. wolfei Goettingen	0	0	0.6	0.55	0.25	1	0.33	0.33	1	0
Syntrophus aciditrophicus SB	0.71	0	0.4	0.55	0.14	1	0.17	1	1	0
Syntrophus aciditrophicus scadc_MAG006	0.71	0	0.4	0.5	0.19	1	0.17	1	1	0
Syntrophus aciditrophicus tb_SCADC009	0.71	0	0.4	0.64	0.63	1	1	1	1	0
Syntrophus scadc_MAG275	0.29	0	0.2	0.5	0.25	1	0.33	0	0.25	0
Thermanaerovibrio acidaminovorans DSM 6589	0.14	0	0.4	0.27	0.63	1	0.67	1	0.75	0
Thermomicrobium roseum DSM 5159	0.57	0	0.6	0.5	0.25	1	0.17	0.33	1	0
Thiobacillus denitrificans ATCC 25259	0	0	0.6	0.5	0.88	1	0.83	0.67	1	0
Myxococcus fulvus HW-1	0.71	0	0.6	0.5	0.22	1	0	1	1	0
Myxococcus stipitatus DSM 14675	0.71	0	0.6	0.5	0.56	1	0	1	0.75	0
Myxococcus xanthus DK 1622	0.71	0	0.6	0.82	0.25	1	0	1	1	0

di-trans,poly-cis-undecaprenyl phosphate	flavin	folate	formylTHF	geranyl diphosphate	glutathione	guanylyl molybdenum cofactor	heme	L-ascorbate	lipoate	lipoate salvage	menaquinol-8	menaquinol-9	methylerythritol phosphate	mevalonate	molybdenum cofactor	NAD	NAD salvage	octaprenyl diphosphate
1	0.67	1	0.83	1	1	1	1	0	1	0	1	0	1	0.29	0.43	0.83	0.75	1
1	0.56	0.8	0.75	1	1	1	0.75	0.33	1	0	1	0	0.89	0.14	0.57	1	0.63	1
1	0.22	1	0.67	1	1	1	0.75	0.33	1	0	1	1	0.78	0.14	0.57	1	0.38	1
1	0.78	1	0.58	1	1	1	1	0.33	1	0	1	1	0.78	0.14	0.57	0.83	0.5	1
1	0.44	0.2	0.67	1	1	1	0.5	0	1	0	1	1	0.78	0.29	0.29	0.83	0.5	0
0	0.44	0.6	0.67	1	1	1	0.75	0	0.5	0	1	0	0.67	0.14	0.29	0.83	0.5	1
1	0.44	0.8	0.75	1	1	1	0.75	0	1	0	1	0	0.89	0.14	0.71	1	0.63	1
1	0.44	1	0.75	1	1	1	0.75	0.33	1	0	1	1	0.89	0.14	0.43	1	0.5	1
1	0.89	0.8	0.75	1	1	1	0.75	0	1	0	1	0	0.89	0.14	0.43	0.83	0.63	1
1	0.78	0.8	0.75	1	1	1	1	0	1	0	1	1	0.89	0.14	0.71	1	0.5	1
1	0.56	0.8	0.75	1	1	1	0.75	0.33	1	0	1	0	0.89	0.14	0.57	1	0.63	1
1	0.67	0.8	0.75	1	1	1	0.75	0	1	0	1	1	0.89	0.14	0.43	1	0.63	1
1	0.67	0.8	0.67	1	1	1	0.75	0.33	1	0	1	0	0.89	0.14	0.57	0.33	0.63	1
1	0.78	0.8	0.75	1	1	1	0.75	0	1	0	1	1	0.89	0.14	0.43	0.83	0.63	1
1	0.56	0.8	0.67	1	1	1	0.75	0.33	1	0	1	1	0.89	0.14	0.43	0.83	0.5	1
1	0.78	0.8	0.75	0	1	1	0.75	0	1	0	1	1	0.89	0.14	0.43	1	0.5	1
1	0.89	0.8	0.67	1	1	0	0.75	0.67	1	1	1	1	0.89	0.14	0.14	1	0.5	0
1	0.89	0.8	0.75	1	1	1	0.75	0	1	0	1	1	0.89	0.14	0.71	1	0.5	1
1	0.78	0.8	0.75	0	1	1	0.75	0.33	1	0	1	1	0.89	0.14	0.43	1	0.5	1
1	0.78	0.8	0.75	0	1	1	0.75	0	1	0	1	1	0.89	0.14	0.14	0.83	0.63	1
1	0.67	0.8	0.75	1	1	1	0.75	0	1	0	1	0	0.89	0.14	0.71	0.83	0.63	1
1	0.78	0.8	0.75	1	1	1	0.75	0.33	1	0	1	1	0.89	0.14	0.43	1	0.63	1
1	0.89	0.8	0.67	1	1	1	0.75	0	1	0	1	0	0.89	0.14	0.29	1	0.63	0
1	0.67	0.8	0.75	1	1	1	0.75	0	1	0	1	0	0.89	0.29	0.57	1	0.63	1
1	0.89	1	0.83	1	1	1	0.75	0	1	0	1	0	0.89	0.14	0.71	0.83	0.5	1
1	0.78	1	0.83	1	1	1	1	0	1	0	1	0	0.78	0.14	0.71	1	0.5	1
1	0	1	0.5	1	0.5	1	0.25	0	0	0	0	0	0.78	0.29	0.71	0.33	0.25	0
1	0	0.4	0.42	1	0.33	0	0.25	0	0.5	1	0	0	0.89	0	0.29	0.33	0.25	0
1	0.22	0.2	0.17	1	0.67	1	0	0	0.5	1	0	0	0.44	0.14	0.14	0.33	0.25	0
1	0.89	1	0.67	1	0.67	0	0.25	0	0	0	0	0	0.89	0.14	0.43	1	0.63	0
1	0.89	1	0.67	1	0.67	0	0.25	0	0	0	0	0	1	0.29	0.43	1	0.63	0
1	0.78	1	0.67	1	0.67	0	0.25	0	0	0	0	0	1	0.29	0.29	1	0.63	0
1	0.89	1	0.75	0	1	1	0.5	0	1	0	1	1	0.89	0	0.57	1	0.5	0
1	0.89	1	0.67	1	0	1	0.5	0	1	1	1	0	1	0.14	0.14	1	0.63	0
1	0.67	0.2	0.67	1	0	1	0.25	0	0.5	1	0	0	0.89	0	0.29	1	0.38	0
1	0.67	0.4	0.67	1	1	1	0.5	0	0	0	1	0	1	0.29	0.43	1	0.5	0
1	0.56	0.4	0.67	0	1	1	0.5	0	0	0	0	0	1	0.29	0.14	1	0.63	0
1	0.67	1	0.58	1	0.67	1	0.5	0	0	0	0	0	1	0.43	0.29	1	0.63	0
1	0.89	1	0.67	1	0	1	0.5	0	0.5	0	0	0	0.89	0	0.43	0.5	0.5	0
1	0.89	1	0.67	1	0	1	0.5	0	0.5	0	0	0	0.89	0	0.43	0.5	0.5	0
1	0.89	1	0.67	0	0	0	0.5	0	0.5	1	0	0	0.89	0.14	0.57	1	0.63	0
1	0.89	1	0.67	1	0	0	0.5	0	0.5	1	0	0	0.89	0.14	0.14	1	0.63	0
1	0.89	1	0.67	1	0	0	0.5	0	0.5	1	0	0	0.89	0.14	0.57	1	0.63	0
1	0.89	1	0.67	1	0	0	0.5	0	0.5	1	0	0	0.89	0.14	0.71	1	0.63	0
1	0.89	1	0.58	1	1	1	0.25	0	0	0	0	0	0.89	0.14	0.29	0.83	0.5	0
1	0.67	0.4	0.67	1	1	1	0.25	0	0	0	0	0	0.89	0.14	0.29	0.83	0.38	0
1	0.78	1	0.58	1	1	1	0.25	0	0	0	0	0	0.89	0.14	0.29	0.83	0.5	0
1	0.89	1	0.58	1	1	1	0.25	0	0	0	0	0	0.89	0.14	0.29	0.83	0.5	0
1	0.78	1	0.75	1	0.33	1	0.5	0	1	1	1	0	0.89	0.14	0.57	0.67	0.25	0
1	0.89	1	0.75	1	0	1	0.25	0	1	1	1	0	0.78	0.14	0.57	0.67	0	0
1	0.78	0.4	0.58	1	1	1	0.25	0	0	0	0	0	1	0.29	0.29	0.67	0.25	0
1	0.44	0.4	0.58	0	0.5	1	0.25	0	0	0	0	0	0.89	0	0.14	1	0.5	0
1	0.67	0.4	0.67	1	0.5	0	0.5	0	0	0	0	0	0.89	0.14	0.14	1	0.5	0
1	0.67	0.4	0.67	1	1	0	0.5	0	0	0	0	0	0.89	0.14	0.14	0.83	0.25	0
1	0.67	0.4	0.58	1	0	0	0.25	0	0	0	0	0	0.89	0.14	0.14	0.33	0.38	0
1	0.67	1	0.75	1	0	1	0.5	0	0.5	1	0	0	0.89	0.14	0.43	1	0.5	0
1	0.33	1	0.75	1	0	1	0.5	0	0.5	1	0	0	0.89	0	0.43	1	0.5	0
1	0.67	1	0.75	1	0	1	0.5	0	0.5	1	0	0	0.89	0	0.43	0.83	0.5	0
1	0.67	1	0.75	1	0	1	0.5	0	0.5	1	0	0	0.89	0	0.43	1	0.5	0
1	0.67	1	0.75	1	0	1	0.25	0	0.5	1	0	0	0.89	0	0.43	1	0.38	0
1	0.33	1	0.75	1	0	1	0.5	0	0.5	1	0	0	0.89	0	0.43	1	0.5	0
1	0.33	1	0.67	1	0	1	0.25	0	0.5	1	1	0	1	0	0.43	0.5	0.63	0
1	0.89	0.8	0.58	1	0	0	0.25	0	0	0	0	0	0.89	0	0.29	1	0.25	0
1	0.22	1	0.67	1	0.67	1	0.25	0	0.5	1	0	0	0.89	0	0.29	0.83	0.5	0
1	0.67	1	0.75	0	0	0	0.5	0	1	1	0	0	0.89	0	0.14	1	0.63	0

1	0.44	0.4	0.33	0	0	0	0.25	0	0.5	0	0	0	0.78	0	0	0.67	0.38	0
1	0.89	1	0.5	1	0.5	0	0.25	0	0	0	0	0	1	0	0.14	1	0.63	0
1	0	0.4	0.58	1	0	0	0.25	0	0.5	1	0	0	0.89	0	0.14	0.83	0.38	0
1	0.22	0.4	0.58	1	0	1	0.25	0	0.5	1	0	0	0.89	0	0.14	0.83	0.38	0
1	0.67	1	0.75	1	0	1	0.5	0	1	1	1	0	1	0	0.29	0.83	0.63	0
1	0	0.4	0.42	1	0	0	0.25	0	0	0	0	0	0.89	0	0.29	0.83	0.38	0
1	0.89	1	0.5	0	0.67	0	0.25	0	0	0	0	0	0.89	0	0.14	1	0.5	0
1	0.78	1	0.58	1	0.67	0	0.5	0	0	0	0	0	0.89	0	0.14	0.83	0.5	0
1	0.67	1	0.58	1	0	1	0.25	0	0	0	0	0	1	0	0.14	1	0.5	0
1	0.78	1	0.75	1	0	1	0.25	0	1	0	1	0	1	0	0.43	1	0.5	0
1	0.78	1	0.42	1	1	1	0.25	0	0	0	0	0	0.89	0	0.43	1	0.5	0
1	0.67	1	0.42	1	1	1	0.25	0	0	0	0	0	0.89	0	0.43	1	0.5	0
1	0.44	1	0.42	0	1	1	0.25	0	0	0	0	0	0.89	0	0.29	1	0.5	0
1	0.11	0.4	0.75	1	0.33	0	0.25	0	0.5	1	0	0	1	0	0.14	0.67	0.5	0
1	0.89	1	0.67	1	1	1	0.25	0	0	0	0	0	1	0	0.57	0.83	0.5	0
1	0.44	1	0.67	0	0	1	0.25	0	0.5	1	0	0	0.89	0	0.14	0.5	0.5	0
1	0.89	1	0.67	1	1	1	0.5	0	0	0	1	0	1	0	0.43	0.83	0.63	0
1	0.89	1	0.67	1	1	1	0.5	0	0	0	1	0	1	0	0.57	0.83	0.63	0
1	0.67	1	0.5	1	0.67	0	0.25	0	0	0	0	0	0.89	0	0.43	0.83	0.38	0
1	0.67	0.4	0.58	1	0	0	0.25	0	0	0	0	0	0.89	0	0.14	0.33	0.38	0
1	0.67	0.4	0.58	1	0	0	0.25	0	0	1	0	0	0.89	0	0.29	0.83	0.38	0
1	0.89	1	0.67	1	0	0	0.5	0	1	1	0	0	0.89	0.14	0.57	0.83	0.25	0
1	0.78	0.4	0.75	1	0	0	0.5	0	1	1	0	0	0.89	0.14	0.57	1	0.38	0
1	0.89	1	0.75	1	0.33	1	0.5	0	1	1	0	0	0.89	0.14	0.57	1	0.25	0
1	0.89	0.2	0.5	1	0	0	0.5	0	0	0	1	0	0.89	0.14	0.29	0.83	0.38	0
1	0.89	1	0.75	1	0.67	0	0.25	0	1	1	1	1	0.89	0.14	0.29	0.83	0.5	0
1	0.44	0.8	0.42	1	0	1	0.25	0	0.5	1	0	0	0.89	0.14	0.14	0.5	0.63	0
1	0.67	0.8	0.5	1	0	0	0.25	0	0	1	0	0	0.89	0.14	0.14	1	0.63	0
1	0.78	0.4	0.67	1	0	0	0.5	0	0	0	0	0	1	0.14	0.14	1	0.38	0
1	0.67	0.4	0.67	1	0	0	0.25	0	0	0	0	0	0.89	0	0.14	0.83	0.38	0
1	0.67	0.4	0.58	1	0	0	0.25	0	0	0	0	0	0.89	0	0.14	1	0.38	0
1	0.67	0.4	0.58	1	0	0	0.25	0	0	0	0	0	0.89	0	0.14	0.83	0.38	0
1	0.67	0.2	0.25	1	0.33	0	0.5	0	0.5	1	0	0	1	0.29	0.43	1	0.63	0
1	0.89	1	0.67	1	0	1	0.25	0	1	0	0	0	0.89	0.14	0.43	0.67	0.25	0
1	0.89	1	0.67	1	0	1	0.25	0	1	0	0	0	0.89	0.14	0.43	0.83	0.38	0
1	0.89	1	0.58	1	0	1	0.25	0	1	0	0	0	0.89	0.14	0.29	0.67	0.25	0
1	0.89	1	0.67	1	0	1	0.5	0	1	0	0	0	0.89	0.14	0.43	0.67	0.25	0
1	0	1	0.42	1	0.5	1	0.25	0	0.5	1	0	0	0.78	0.71	0.57	0.5	0.63	0
1	0.89	0.8	0.58	1	1	1	0.75	0	1	0	1	0	0.78	0.29	0.86	1	0.5	1
1	0.89	0.8	0.75	1	1	1	0.75	0	1	0	1	0	0.89	0.14	0.86	1	0.63	1
1	0.78	0.8	0.67	1	1	1	0.75	0	1	0	1	0	0.89	0.14	0.71	1	0.63	1
1	0.89	0.8	0.67	1	1	1	0.75	0	1	1	1	0	0.89	0.14	0.71	1	0.63	1
0	0.89	0.8	0.67	1	1	1	0.5	0	0	0	1	0	0.56	0.14	0.71	1	0.63	1
1	0.89	0.8	0.58	1	0.67	1	0.75	0.33	1	0	1	0	1	0.29	0.86	1	0.63	1
1	0.67	0.8	0.58	1	1	1	0.75	0	1	0	1	0	0.89	0.14	0.86	1	0.5	1
1	0.89	0.8	0.58	1	1	1	0.75	0	1	0	1	0	0.89	0.14	0.86	1	0.75	1
1	0.89	0.8	0.58	1	1	1	0.75	0.33	1	0	1	0	0.89	0.29	0.86	0.83	0.75	1
1	0.67	0.8	0.58	0	0.67	1	0.75	0	0.5	0	1	0	0.56	0.14	0.43	0.67	0.5	0
1	0.89	0.8	0.58	1	1	1	0.75	0.67	1	0	1	0	1	0.29	0.86	1	0.75	1
1	0.89	1	0.67	1	1	1	0.75	0	1	0	1	0	0.89	0.14	0.86	0.83	0.63	1
1	0.78	0.8	0.67	1	1	1	0.75	0	1	0	1	0	0.89	0.14	0.71	1	0.5	1
1	0.89	1	0.83	1	0	1	1	0	1	0	1	0	1	0	0.71	1	0.63	0
1	0.89	1	0.67	1	0	1	0.5	0	0.5	1	1	0	0.89	0	0.57	1	0.38	0
1	0.67	1	0.42	1	0	1	0.25	0	0	0	1	1	0.89	0	0.14	1	0.25	0
1	0.89	1	0.42	0	0	1	0.25	0	0	0	0	0	0.89	0	0.14	0.67	0.38	0
1	0.89	1	0.42	0	0	0	0.25	0	0	0	0	0	0.89	0	0	0.83	0.38	0
1	0.89	1	0.42	0	0	0	0.25	0	0	0	0	0	0.89	0	0.14	0.83	0.38	0
1	0.89	0.8	0.58	0	0	1	0.5	0	0	0	0	0	0.78	0	0.71	1	0.25	0
1	0.78	0.4	0.42	0	0	1	0.25	0	0	0	1	0	0.89	0	0	1	0.38	0
1	0.89	1	0.42	0	0	1	0.25	0	0	0	0	0	0.89	0	0.14	0.83	0.38	0
1	0.67	0.4	0.33	0	0	0	0.25	0	0	0	0	0	0.89	0	0	1	0.25	0
1	0.78	1	0.42	0	0	1	0.25	0	0	0	0	0	0.89	0	0.14	0.83	0.38	0
1	0.67	1	0.5	0	0	1	0.5	0	0	0	0	0	0.89	0	0.14	0.83	0.25	0
1	0.89	1	0.75	1	0	1	0.5	0	0	0	1	1	1	0	0.71	1	0.25	0
1	0.78	1	0.67	1	0	1	0.5	0	0.5	1	1	1	0.89	0	0.57	1	0.63	0
1	0.78	1	0.67	1	0	0	0.5	0	0.5	1	1	1	0.89	0	0.29	0.83	0.38	0

1	0.67	1	0.67	1	0	0	0.5	0	0.5	1	0	0	1	0	0.14	0.83	0.25	0
1	0.89	0.4	0.67	1	0	1	0.5	0	0	0	1	0	0.89	0	0.57	1	0.38	0
1	0.89	0.4	0.67	1	0	1	0.5	0	0	1	1	0	0.89	0	0.57	1	0.38	0
1	0.89	0.4	0.67	0	0	1	0.5	0	0	1	1	0	0.89	0	0.57	1	0.38	0
0	0.89	0.4	0.17	0	0	0	0.25	0	0	0	0	0	0.33	0	0	1	0.38	0
1	0.89	0.4	0.75	1	0	1	0.5	0	0.5	1	1	0	0.89	0	0.57	1	0.38	0
1	0.89	1	0.5	1	0	1	0.25	0	0.5	0	1	0	0.89	0	0.14	1	0.38	0
1	0.67	0.4	0.83	1	0	1	0.25	0	1	1	1	0	0.89	0	0.29	1	0.5	0
1	0.67	0.4	0.67	1	0	1	0.25	0	1	0	1	0	0.89	0	0.29	1	0.38	0
1	0.89	1	0.75	0	0	1	0.5	0	1	1	0	0	0.89	0	0.29	1	0.63	1
1	0.89	1	0.75	1	0	1	0.25	0	1	0	1	0	1	0	0.43	1	0.5	1
1	0.89	0.8	0.67	1	0	1	0.75	0	1	0	1	0	1	0	0.43	1	0.5	0
1	0.89	0.8	0.75	1	0	1	0.75	0	1	0	1	0	1	0	0.57	0.83	0.5	0
1	0.67	1	0.58	0	0	1	0.5	0	0.5	1	0	0	0.89	0	0.14	0.67	0.25	0
1	0.22	0.2	0.17	0	0	0	0.25	0	0	0	0	0	0.56	0	0	0.33	0.13	0
1	0.89	1	0.67	1	0	1	0.5	0	0.5	1	1	1	0.89	0	0.57	1	0.5	0
1	0.78	1	0.67	1	0.67	1	0.5	0	0.5	1	1	1	0.89	0	0.57	1	0.63	0
1	0.67	1	0.67	1	0.67	1	0.5	0	1	1	0	0	0.89	0	0.14	0.67	0.5	0
1	0.89	1	0.75	1	0	1	0.5	0	0.5	1	1	0	0.89	0	0.29	1	0.5	0
1	0.89	1	0.67	1	0	1	0.5	0	0.5	0	1	1	0.89	0	0.57	1	0.5	0
1	0.44	1	0.67	1	0	1	0.5	0	0	0	0	0	0.89	0	0.14	0.83	0.38	0
1	0.89	1	0.58	1	0	1	0.25	0	0	0	1	0	1	0	0.29	1	0.5	0
1	0.89	1	0.67	1	0.5	1	0.25	0	1	0	1	0	1	0	0.14	1	0.38	0
1	0.67	1	0.67	1	0	1	0.5	0	1	0	0	0	1	0	0.14	1	0.5	0
1	0.89	0.4	0.58	1	0	1	0.25	0	1	0	1	0	1	0	0.43	1	0.25	0
1	0.89	0.4	0.58	1	0	1	0.25	0	1	0	1	0	1	0	0.29	1	0.25	0
1	0.89	1	0.58	0	0	1	0.5	0	0	0	1	0	1	0	0.14	1	0.25	0
1	0.67	0.4	0.75	1	0	1	0.25	0	1	0	0	0	1	0	0.29	1	0.25	0
1	0.44	1	0.67	1	0.5	0	0.5	0	0.5	1	0	0	1	0	0.14	0.83	0.25	0
1	0.44	0.8	0.58	0	0	1	0.25	0	1	0	0	0	1	0	0.29	1	0.25	0
1	0.67	0.2	0.67	1	0	1	0.25	0	1	0	1	0	1	0	0.43	0.83	0.5	0
1	0.44	0.8	0.5	1	1	1	0.25	0	1	0	0	0	1	0	0.14	0.83	0.38	0
1	0.44	0.2	0.58	1	0	1	0.25	0	1	0	1	0	0.89	0	0.43	0.83	0.5	0
1	0.67	0.2	0.67	1	0	1	0.5	0.33	1	1	1	1	1	0	0.43	0.83	0.5	0
1	0.44	0.8	0.5	0	0	0	0.25	0	1	1	0	0	1	0	0.14	0.83	0.38	0
1	0.67	1	0.67	1	0	1	0.25	0	1	0	1	0	1	0	0.29	1	0.25	0
1	0.44	1	0.58	0	0	1	0.25	0	1	1	1	0	0.89	0	0.14	0.83	0.13	0
1	0.67	0.4	0.58	1	0	1	0.25	0	1	1	1	0	0.89	0	0.29	0.83	0.25	0
1	0.89	0.8	0.58	1	0	1	0.25	0	1	0	1	0	1	0	0.14	1	0.25	0
1	0.33	0.2	0.5	0	0.5	1	0.25	0	0.5	0	1	0	0.22	0	0.29	0.33	0	0
1	0.67	0.8	0.58	0	0.5	1	0.25	0	1	1	1	0	0.89	0	0.29	0.83	0.38	0
1	0.44	0.2	0.67	1	0	1	0.25	0	1	0	0	0	0.89	0	0.29	1	0.25	0
1	0.44	0.8	0.75	1	0	0	0.25	0	1	0	0	0	1	0	0.29	1	0.25	0
1	0.67	0.2	0.67	1	0.5	1	0.25	0	1	1	0	0	1	0	0.43	0.83	0.38	0
1	0.67	0.2	0.67	1	0	1	0.25	0	1	0	1	0	0.89	0	0.43	0.83	0.38	0
1	0.89	1	0.75	1	1	1	0.5	0	1	1	1	0	1	0	0.43	0.83	0.5	1
1	0.89	0.8	0.67	1	1	1	0.25	0	0.5	1	1	0	1	0	0.43	0.83	0.5	1
1	0.67	0.2	0.67	1	0	1	0.5	0	1	1	1	0	0.78	0	0.29	0.83	0.5	0
1	0.44	0.8	0.67	1	0	1	0.25	0	1	0	1	0	0.89	0	0.43	0.83	0.25	0
1	0.44	0.8	0.75	0	0	1	0.25	0	1	0	0	0	1	0	0.43	0.83	0.25	0
1	0.67	0.4	0.5	0	0	0	0	0	0.5	1	1	0	0.89	0	0.29	0.5	0.13	0
1	0.67	0.8	0.58	1	0	1	0.25	0.33	1	0	1	0	1	0	0.57	0.83	0.38	1
1	0.44	0.8	0.58	1	0	0	0.25	0	1	0	0	0	1	0	0.29	1	0.25	0
1	0.67	0	0.5	1	0	1	0	0	0.5	0	1	0	0.67	0	0.43	0.83	0.25	0
1	0.44	1	0.75	1	0	1	0.5	0	0.5	1	1	0	1	0	0.29	0.83	0.38	0
1	0.89	0.8	0.58	1	0	1	0.25	0	1	0	1	0	0.89	0	0.29	0.83	0.25	0
1	0.33	0.4	0.83	0	0	1	0.25	0	0.5	1	1	0	0.33	0	0.29	0.5	0.13	1
1	0.67	0.8	0.67	0	0.5	1	0.5	0.33	1	0	1	0	0.89	0	0.29	0.83	0.5	0
1	0.44	0.8	0.58	1	0.5	1	0.25	0	1	1	0	0	1	0.14	0.14	1	0.38	1
1	0.67	0.2	0.67	1	0	1	0.25	0	1	0	1	0	0.78	0.14	0.29	0.83	0.5	0
1	0.44	0.2	0.58	1	0.5	1	0.25	0	1	1	1	0	0.89	0	0.43	0.83	0.5	0
1	0.44	0.8	0.75	0	0	1	0.25	0	1	0	0	0	1	0.14	0.14	1	0.25	0
1	0.89	0.8	0.67	1	0	1	0.25	0	1	0	1	0	1	0.14	0.29	0.83	0.38	1
1	0.44	0.8	0.75	0	0	0	0.25	0	1	0	1	0	1	0.14	0.14	1	0.25	0
1	0.44	0.8	0.58	1	0	1	0.25	0	1	0	0	0	1	0.14	0.14	0.83	0.25	0
1	0.56	0.8	0.5	0	0	0	0	0	1	0	0	0	0.89	0	0.14	0.83	0.25	0

0	0.89	0.2	0.58	1	0	0	0.25	0	0.5	1	1	1	0.89	0.29	0.14	0.33	0.13	0
1	0	1	0.25	1	0	0	0.25	0	0.5	0	0	0	0.89	0	0.14	0.67	0.25	0
0	0	1	0.5	1	0	0	0.25	0	0	0	0	0	0.78	0.14	0.43	0.67	0.25	0
1	0.89	0.8	0.67	1	0	0	0.75	0	1	1	0	0	0.89	0.14	0.29	1	0.63	1
1	0.44	0.8	0.5	1	0	1	0.75	0	1	1	0	0	0.89	0.14	0.14	1	0.63	0
1	0.67	0.8	0.5	0	0	1	0.75	0	1	1	0	0	0.89	0	0.29	1	0.38	0
0	0.56	0.2	0.42	1	0	1	0.75	0	0.5	0	1	0	0.78	0	0.29	0.83	0.38	1
1	0.89	0.8	0.67	1	0	0	1	0	1	1	1	0	0.89	0.14	0.43	1	0.63	1
1	0.67	0.2	0.58	1	0	1	0.75	0	0.5	0	1	0	0.89	0	0.29	0.67	0.5	1
1	0.67	0.8	0.5	1	0	1	0.75	0	1	1	0	0	0.89	0.14	0.14	0.83	0.5	0
1	0.67	0.8	0.5	0	0	1	0.75	0	1	1	0	0	0.89	0.14	0.14	1	0.63	0
1	0.89	0.8	0.58	0	0	0	0.75	0	1	1	1	0	0.89	0.14	0.71	1	0.63	1
1	0.89	0.8	0.58	1	0	0	0.75	0	1	1	1	0	0.89	0.14	0.71	1	0.63	1
1	0.33	0.8	0.5	1	0	1	0.75	0	1	1	0	0	0.89	0.14	0.29	1	0.63	0
1	0.44	0.6	0.5	0	0.5	0	0.5	0	1	0	0	0	0.44	0	0.29	0.33	0.13	0
1	0	1	0.33	1	0.67	0	0.5	0	1	1	0	0	0.89	0	0.14	0.67	0.5	0
1	0	0.2	0.42	1	1	1	0.25	0	0	0	0	0	0.11	0	0.43	0.5	0.13	0
1	0	0.2	0.42	1	0	1	0	0	0	0	0	0	0.11	0	0.29	0.67	0.13	0
0	0	0.2	0.08	1	0	1	0	0	0	0	0	0	0.11	0	0.57	0	0	0
0	0	0.2	0.33	1	0	1	0	0	0	0	0	0	0.11	0	0.57	0.67	0.13	0
0	0	1	0.58	1	0.33	0	0.75	0	0.5	0	1	0	0.11	0	0.43	0.67	0.25	1
1	0	0.2	0.33	0	0	1	0	0	0	0	0	0	0.11	0	0.57	0.67	0.13	0
1	0	0	0.33	0	0	0	0.25	0	0	0	0	0	0	0	0.29	0.33	0.13	0
1	0	0.2	0.25	1	0.5	1	0	0	0	0	0	0	0.11	0	0.43	0.5	0.13	0
0	0	0.2	0.25	0	0	1	0	0	0	0	0	0	0.11	0	0.57	0.33	0.13	0
1	0	0.2	0.25	1	1	1	0	0	0	0	0	0	0.11	0	0.43	0.67	0.13	0
1	0	0.2	0.33	1	1	1	0.25	0	0	0	1	0	0.11	0	0.43	0.67	0.38	0
0	0	0.2	0.17	1	0.5	0	0	0	0	0	1	0	0.11	0	0.14	0.67	0.38	0
1	0	0.2	0.42	1	0	1	0	0	0	0	0	0	0.11	0	0.29	0.5	0.25	0
1	0.67	1	0.42	1	0	1	0.25	0	0	0	1	1	0.89	0	0.14	1	0.25	0
1	0.89	1	0.42	0	0	1	0.25	0	0	0	0	0	0.89	0	0.14	0.67	0.38	0
1	0.89	1	0.42	0	0	0	0.25	0	0	0	0	0	0.89	0	0	0.83	0.38	0
1	0.89	1	0.42	0	0	0	0.25	0	0	0	0	0	0.89	0	0.14	0.83	0.38	0
1	0.89	0.8	0.58	0	0	1	0.5	0	0	0	0	0	0.78	0.14	0.71	1	0.25	0
1	0.78	0.4	0.42	0	0	1	0.25	0	0	0	1	0	0.89	0	0	1	0.38	0
1	0.89	1	0.42	0	0	1	0.25	0	0	0	0	0	0.89	0	0.14	0.83	0.38	0
1	0.67	0.4	0.33	0	0	0	0.25	0	0	0	0	0	0.89	0	0	1	0.25	0
1	0.78	1	0.42	0	0	1	0.25	0	0	0	0	0	0.89	0	0.14	0.83	0.38	0
0	0.33	0	0.25	0	0	0	0	0	0.5	0	1	0	0.22	0	0	0.33	0.25	0
1	0.89	0.8	0.58	1	0.5	1	0.75	0	1	0	1	0	1	0.14	0.71	1	0.63	1
0	0.33	0	0.33	1	0.5	0	0.25	0	0.5	0	1	0	0.44	0	0.43	0.67	0.38	0
1	0.89	1	0.58	1	0	1	0.25	0	0	0	1	0	0.78	0.14	0.43	0.67	0.25	0
1	0.78	1	0.5	1	0	0	0.5	0	0	0	1	0	0.89	0	0.29	0.67	0.25	0
1	0.89	0.4	0.67	1	0	0	0.25	0	0	0	0	0	1	0.14	0.29	1	0.38	0
1	0.67	0.4	0.83	0	0	0	0.75	0	1	0	1	0	0.78	0.14	0.14	0.83	0.25	0
1	0.89	0.4	0.67	1	0	0	0.25	0	0	0	0	0	0.89	0	0.29	1	0.25	0
1	0.22	1	0.58	1	0	0	0.25	0	0.5	1	0	0	0.89	0.14	0.29	1	0.25	0
1	0.89	1	0.5	1	0	1	0.5	0	0	0	1	0	0.89	0.14	0.57	0.83	0.25	0
0	0.33	0.8	0.25	1	0	1	0.25	0	0	0	0	0	0.78	0.29	0.43	0.67	0.13	0
1	0.22	0.8	0.42	0	0	0	0.25	0	0.5	1	0	0	1	0.14	0.14	0.5	0.38	0
1	0.11	0.8	0.58	1	0.67	0	0.5	0	0.5	1	0	0	0.89	0.29	0.14	0.67	0.5	0
1	0.56	0.4	0.75	0	0	1	0.5	0	0.5	1	0	0	1	0.29	0.29	1	0.25	0
1	0.78	1	0.58	1	0	1	0.25	0	0	0	0	0	0.89	0.14	0.14	1	0.5	0
1	0.89	0.4	0.67	1	0	1	0.5	0	0	0	1	0	0.89	0.14	0.43	0.83	0.13	0
1	0.89	1	0.75	1	0	1	0.25	0	1	0	1	0	0.89	0.29	0.71	1	0.63	0
1	0.11	0.6	0.17	1	0	0	0.25	0	0	0	1	0	0.67	0.14	0.57	0.5	0.25	0
1	0.67	0.4	0.5	0	0	0	0.25	0	0	0	0	0	0.89	0	0.14	0.83	0.25	0
1	0.78	1	0.58	0	0	0	0.75	0	1	1	1	0	0.78	0.14	0.29	0.67	0.63	0
1	0.89	0.8	0.67	1	1	1	0.75	0.33	1	0	1	0	0.89	0	0.29	1	0.25	0
1	0.56	1	0.67	1	1	1	1	0.33	1	0	0	0	0	0.86	0.14	0.83	0.63	0
1	0.56	1	0.67	1	1	1	1	0	1	0	0	0	0	1	0.14	1	0.63	0
1	0.89	1	0.67	1	1	1	1	0	1	0	1	0	0	0.86	0.43	0.83	0.5	0

p-aminobenzoate	phosphopantothenate	pyridoxal 5'-phosphate	pyridoxal 5'-phosphate salvage	S-adenosyl-L-methionine	tetrahydrofolate	tetrahydrofolate salvage	tetrapyrrole	thiamin	thiamin salvage	thio-molybdenum cofactor	thioredoxin	trans, trans-farnesyl diphosphate	ubiquinol
0.5	0.75	0.71	0.8	1	1	1	1	0.67	1	0	0.5	0.67	0.75
0.5	1	0.86	1	1	1	1	1	0.67	1	0	0.5	0.67	0.75
0.5	1	0.71	0.4	1	0.67	1	0.75	1	1	0	0.5	0.67	0.75
0.5	1	0.71	0.8	1	1	0.5	0.75	0.67	1	0	0.5	0.67	0.75
1	0.5	0.71	0.8	1	0.67	1	0.33	0.5	0	0	0.5	0.33	0.63
0.5	1	0.86	0.8	1	0.67	0.5	0.75	0.67	1	0	0.5	0.67	0.75
1	1	0.86	1	1	1	1	1	1	1	0	0.5	0.67	0.63
0.5	1	0.86	0.8	1	0.67	1	1	1	1	0	0.5	0.67	0.63
0.5	1	0.86	0.8	1	0.67	1	1	0.67	1	0	0.5	0.67	0.63
1	1	0.71	1	1	1	1	1	1	1	0	0.5	0.67	0.63
0.5	1	0.86	1	1	1	1	1	0.67	1	0	0.5	0.67	0.75
0.5	0.75	0.86	0.6	1	1	1	1	0.67	1	0	0.5	0.67	0.88
0.5	0.25	0.86	0.6	1	1	1	1	0.5	0	0	0.5	0.67	0.63
0.5	0.75	0.86	0.6	1	1	1	1	0.67	1	0	0.5	0.67	0.88
0.5	0.75	0.86	0.6	1	0.67	1	1	0.67	1	0	0.5	0.67	0.88
0.5	1	0.71	1	1	1	1	1	1	1	0	0.5	0.33	0.63
1	1	0.71	0.8	1	0.67	1	1	0.67	1	1	0.5	0.67	0.63
0.5	0.75	0.71	1	1	1	1	1	0.67	0	0	0.5	0.67	0.75
0.5	0.75	0.71	1	1	1	1	1	0.67	0	0	0.5	0.33	0.5
1	1	0.86	0.6	1	0.67	0.5	1	0.67	1	0	0.5	0	0.88
0.5	0.75	0.86	1	1	1	1	1	0.5	0	0	0.5	0.67	0.75
0.5	1	0.86	1	1	1	1	1	0.67	1	0	0.5	0.67	0.75
1	1	0.71	0.4	1	0.67	1	1	1	1	1	0.5	0.67	0.5
1	1	0.86	1	1	1	1	1	0.67	1	0	0.5	0.67	0.63
0.5	0.75	0.71	1	1	1	1	0.75	1	1	0	0.5	0.67	0.88
0.5	1	0.71	1	1	1	1	0.75	1	1	0	0.5	0.67	0.88
0.5	0.75	0.29	0.4	1	0	1	0.17	0.33	1	0	0.5	1	0
0.5	0	0.43	0	1	0	0.5	0.17	1	1	0	0.5	0.67	0.13
1	0	0	0	1	0.33	0.5	0.17	0	0	0	0.5	0.67	0.25
0.5	1	0.14	0.4	1	1	1	1	1	0	0	0.5	0.67	0
0.5	1	0.14	0.2	1	1	1	1	1	0	0	0.5	1	0.13
0.5	1	0.14	0.2	1	1	1	1	1	0	0	0.5	1	0
1	0.75	0.14	0	1	1	0.5	0.83	1	1	0	0.5	0.33	0.25
1	0.5	0.29	0	1	0.67	1	1	1	1	0	0.5	0.67	0.13
0.5	0.75	0.43	0.4	1	0.33	1	1	0.33	1	0	0.5	0.67	0.13
1	1	0.57	0.4	1	0.67	1	0.83	0.67	1	0	0.5	1	0.13
1	1	0.29	0.2	1	0.67	1	1	1	1	0	0.5	0.67	0.13
1	1	0.29	0.4	1	0.67	1	1	1	1	1	0.5	1	0.25
0.5	0.75	0.14	0.4	1	1	1	1	0.67	1	0	0.5	0.67	0.25
0.5	0.75	0.29	0.8	1	1	1	1	0.67	1	0	0.5	0.67	0.25
0	1	0.43	1	1	0.67	1	0.67	1	1	0	0.5	0	0
0	1	0.43	0.8	1	0.67	1	0.67	1	1	0	0.5	0.67	0
0	1	0.29	1	1	0.67	1	0.67	1	1	0	0.5	0.67	0
0	1	0.43	1	1	0.67	1	0.67	1	1	0	0.5	0.67	0
0.5	1	0.43	0.8	1	0.67	1	0.67	0.67	1	0	0.5	0.67	0.25
0.5	1	0.29	0.2	1	0.67	1	0.83	0.67	1	0	0.5	0.67	0.13
0.5	1	0.29	0.6	1	0.67	1	1	0.67	1	0	0.5	0.67	0.25
0.5	1	0.43	0.8	1	0.67	1	0.83	0.67	1	0	0.5	0.67	0.25
0.5	0.5	0.57	0.4	1	0.67	1	0.83	0.67	1	0	0.5	0.67	0.25
0.5	0.5	0.57	0.4	0	0.67	1	0.17	0.67	1	0	0.5	0.67	0.25
0.5	0.75	0.29	0.4	1	0.67	1	0.83	0.67	1	0	0	1	0.25
1	0.75	0.29	0	1	0.67	1	1	1	1	0	0.5	0.33	0.38
1	1	0.29	0	1	0.67	1	1	1	1	0	0.5	0.33	0.38
1	1	0.29	0	1	0.67	1	0.83	0.67	1	0	0	0.67	0.38
0.5	0.5	0.43	0	1	0.33	1	0.33	0.67	1	0	0.5	0.67	0
1	1	0.14	0	1	0.67	1	0.67	0.67	1	1	0.5	0.67	0.13
1	1	0.14	0.4	1	1	1	0.5	0.67	1	1	0.5	0.67	0.13
1	1	0.14	0	1	0.33	1	0.67	0.67	1	1	0.5	0.67	0.13
1	1	0.14	0	1	0.67	1	0.67	0.67	1	1	0.5	0.67	0.13
1	1	0.29	0.4	1	0.67	1	0.67	0.67	1	1	0.5	0.67	0.25
1	1	0.14	0.4	1	1	1	0.67	0.67	1	1	0.5	0.67	0.13
0.5	0.5	0.57	0.4	1	0.33	1	0.33	0.5	0	0	0.5	1	0.13
0	0.75	0.43	0.8	1	0.67	1	0.5	0.67	1	0	0.5	0.67	0
0	0.5	0.71	0.8	1	0.67	1	0.5	0.67	1	0	0.5	0.67	0
0	1	0.14	0	1	1	1	0.83	0.5	0	0	0.5	0.33	0

0	0.75	0.14	0	1	0	0.5	0.83	0.5	0	0	0	0	0
1	0.75	0.29	0	1	0.33	0.5	1	0.67	1	0	0.5	0.33	0.5
0.5	0.25	0.43	0.4	1	0.33	1	0.17	0.33	1	0	0.5	0.67	0
0.5	0.25	0.43	0.4	1	0.33	1	0.17	0.33	1	0	0.5	0.67	0
1	0.5	0.29	0.2	1	1	1	1	1	1	0	0.5	0.67	0.25
0.5	0.5	0.29	0	1	0.33	1	0.17	0.5	0	0	0.5	0.67	0
0.5	1	0.29	0	1	0.67	1	0.83	1	1	0	0.5	0.33	0
1	0.75	0.57	0.4	1	0.67	1	1	0.67	1	0	0.5	0.67	0.13
1	1	0.43	0.4	1	0.67	1	1	1	1	0	0.5	0.67	0
1	1	0.14	0	1	0.67	1	0.83	0.67	1	0	0	1	0.25
0.5	0.5	0.14	0.4	1	1	1	1	0.67	1	0	0.5	0.67	0
1	0.5	0.14	0.4	1	1	1	1	0.67	1	0	0.5	0.67	0
1	0.5	0.14	0.4	1	1	1	1	0.67	1	0	0.5	0.33	0.13
0	0.25	0.29	0.4	1	0	1	1	0.5	1	0	0.5	0.67	0.25
0.5	1	0.43	0.8	1	1	1	1	0.67	1	0	0.5	1	0
0.5	0.25	0.43	0.4	1	0.33	1	1	1	1	0	0.5	0.33	0.25
1	1	0.57	0.8	1	1	1	1	1	1	0	0.5	1	0.25
0.5	1	0.57	0.8	1	1	1	1	0.67	1	0	0.5	1	0.25
0.5	0.75	0.43	0.8	1	0.67	1	0.17	0.67	1	0	0.5	0.67	0
0	0.75	0.43	0	1	0	1	0.5	0.67	1	0	0.5	0.67	0
0	0.5	0.43	0	1	0.67	1	0.83	0.67	1	0	0.5	0.67	0.13
0	1	0.57	0.8	1	0.67	1	0.5	0.67	1	0	0.5	0.67	0.13
0	1	0.43	0.8	1	0.33	1	0.5	0.5	1	0	0.5	0.67	0
1	0	0.14	0	1	1	1	0.83	0.5	1	0	0.5	0.67	0.13
0.5	0.5	0.57	0.8	1	0.33	1	0.33	0.67	1	0	0.5	0.67	0.13
1	1	0.57	0.4	1	1	1	1	0.67	1	0	0.5	0.67	0
0.5	0.25	0.14	0.4	1	0.33	1	1	0.5	1	0	0.5	0.33	0
1	0.5	0.43	0.6	1	0.67	1	1	0.5	1	0	0.5	0.67	0
0.5	0.75	0.43	0	1	0.67	1	1	1	1	0	0.5	1	0.13
1	0.75	0.43	0	1	0.33	1	0.83	0.67	1	0	0	0.67	0.13
1	0.75	0.43	0	1	0.33	1	1	0.67	1	0	0	0.67	0.13
1	0.75	0.43	0	1	0.33	1	1	0.67	1	0	0	0.67	0.13
0.5	0.75	0.14	0	1	0	1	0.83	0.5	0	0	0.5	1	0.13
0.5	1	0.43	0	1	1	1	1	0.5	1	0	0.5	0.67	0.13
0.5	1	0.29	0	1	1	1	1	1	1	0	0.5	0.67	0
0.5	1	0.43	0	1	1	1	0.83	0.5	1	0	0.5	0.67	0.13
0.5	1	0.43	0	1	1	1	1	0.5	1	0	0.5	0.67	0.13
0	0.25	0	0	1	0	1	0.17	0.33	1	0	0.5	1	0.13
1	1	0.71	0.4	1	1	1	0.67	1	0	0	0.5	0.67	0.75
0.5	0.75	0.71	1	1	1	1	1	1	0	0	0.5	0.67	0.88
0.5	1	0.71	1	1	1	1	1	1	0	0	0.5	0.67	0.88
1	1	0.71	0.4	1	1	1	0.83	1	1	0	0.5	0.67	1
0.5	0.5	0.71	1	1	1	1	1	1	0	0	0.5	0.67	0.75
1	1	0.71	0.4	1	1	1	0.83	0.67	0	1	0.5	1	0.88
1	0.75	0.71	0.4	1	1	1	1	1	0	0	0.5	0.67	1
0.5	0.75	0.71	1	1	1	1	1	0.67	0	0	0.5	0.67	0.75
1	0.75	0.86	0.4	1	1	1	1	0.67	0	1	0.5	0.67	0.88
1	1	0.43	0.4	1	1	1	0.5	0.5	0	0	0.5	0	0.75
1	1	0.86	0.4	1	1	1	0.83	1	1	0	0.5	1	1
1	1	0.71	0.4	1	1	1	0.83	0.67	0	0	0.5	0.67	0.88
1	0.75	0.71	0.4	1	1	1	1	1	0	0	0.5	0.67	1
1	1	0.57	0.6	1	1	1	1	1	1	1	0.5	1	0.13
0.5	0.75	0.43	0.4	1	1	1	1	0.33	1	0	0.5	0.67	0.13
0	0.75	0.14	0	1	0.67	0.5	0.17	1	0	0	0.5	0	0.13
0	0.75	0.14	0	1	0.33	1	0.17	1	0	0	0.5	0	0
0	0.75	0.14	0	1	0.67	1	0.17	1	0	0	0.5	0	0
0	0.75	0.14	0.2	1	0.67	1	0.17	1	0	0	0.5	0	0.13
0.5	1	0	0	1	0.33	1	0.17	1	0	0	0.5	0	0
0	0.75	0.29	0	1	0.33	1	0.17	1	0	0	0.5	0	0
0.5	0.75	0.14	0	1	0.67	1	0.17	1	0	0	0.5	0	0
0.5	0.5	0.14	0	1	0.33	1	0.17	1	0	0	0.5	0	0.25
0	0.5	0.14	0	1	0.67	1	0.17	1	0	0	0.5	0	0.13
0.5	0.75	0.14	0.2	1	0.33	1	1	1	0	0	0.5	0	0.13
1	1	0.14	0	1	0.33	1	0.33	1	1	0	0.5	0.33	0.25
0.5	1	0.29	0.4	1	0.67	1	1	1	1	0	0.5	0.33	0.25
0.5	1	0.43	0.4	1	0.67	1	1	0.5	1	0	0.5	0.33	0.13

1	1	0.43	0.6	1	0.33	1	1	1	1	0	0.5	0.67	0.38
0.5	1	0.57	0.8	1	0.33	1	0.83	0.67	1	0	0.5	0.67	0.25
1	1	0.43	0.4	1	0.33	1	1	0.5	0	1	0	0.67	0.25
1	1	0.57	0.4	1	0.33	1	1	0.67	1	0	0	0	0.5
0	0.75	0.43	0.4	1	0	0	0.83	0.5	0	0	0	0	0
0.5	1	0.29	0.4	1	0.33	1	1	0.67	1	0	0.5	0.67	0.13
0.5	0.5	0.43	0.2	1	0.33	1	1	1	0	0	0.5	0	0.38
0	0.75	0.86	0.4	1	0.67	0.5	0.83	0.67	1	0	0.5	0.67	0.13
0.5	1	0.43	0.4	1	0.33	1	0.67	1	1	0	0.5	0.67	0.5
1	1	0.86	0.8	1	1	1	1	1	1	0	0.5	0	0.5
1	0.75	0.86	0.4	1	1	1	0.83	1	1	0	0.5	1	0.5
1	1	0.71	0.4	1	0.67	1	1	1	0	0	0.5	1	0.25
1	1	0.71	0.4	1	0.67	0.5	1	1	0	0	0.5	1	0.25
1	0.75	0.14	0	1	0.33	1	1	1	1	0	0.5	0.33	0.25
0.5	0.25	0	0	1	0.33	0.5	0.33	0.33	1	0	0.5	0	0.13
1	1	0.43	0.8	1	0.33	1	1	1	1	0	0.5	0.33	0.25
0.5	1	0.43	0.8	1	0.67	1	1	1	1	0	0.5	0.33	0.38
1	1	0.43	0.8	1	0.33	1	1	1	1	0	0.5	0	0.38
1	1	0.43	0.8	1	0.67	1	1	0.67	1	0	0.5	0.67	0.5
1	1	0.29	0.4	1	0.67	1	1	0.67	1	0	0.5	0.67	0.5
1	0.75	0.14	0	1	0.33	1	1	1	1	0	0.5	0.33	0.5
1	0.75	0.14	0	1	0.33	1	1	0.67	1	0	0.5	1	0.38
1	0.75	0.14	0.2	1	0.33	1	1	1	0	0	0.5	0.33	0.25
0.5	0.75	0.14	0	1	0.33	1	1	1	1	0	0.5	0.33	0.13
0.5	0.75	0.14	0	1	0.33	1	0.83	1	1	0	0.5	1	0.25
0.5	0.75	0.29	0	1	0.33	1	0.83	1	1	0	0.5	1	0.25
1	0.75	0.14	0.2	1	0.33	0.5	1	1	0	0	0.5	0.67	0.25
1	0.75	0.14	0	1	0.33	1	1	1	1	0	0.5	0.67	0.38
1	0.5	0.14	0.2	1	0.33	1	1	1	1	0	0.5	0.33	0.25
1	0.75	0.43	0.2	1	0.33	1	1	1	0	0	0.5	0.67	0.25
0.5	0.75	0.43	0	1	0.67	1	1	1	0	0	0.5	1	0.38
1	0.5	0.43	0	1	0.33	0.5	1	1	0	0	0.5	0.67	0.25
1	0.75	0.57	0.4	1	0.33	1	0.83	1	0	0	0.5	0.67	0.38
0.5	0.75	0.43	0	1	0.33	1	1	1	0	0	0.5	1	0.75
1	0.5	0.43	0.2	1	0.33	1	1	1	0	0	0.5	0.67	0.25
1	0.75	0.43	0	1	0.33	1	1	1	0	0	0.5	1	0.25
0.5	0.75	0.14	0	1	0.33	1	1	1	0	0	0.5	0.33	0.25
0.5	0.75	0.29	0	1	0.33	1	1	1	0	0	0.5	0.67	0.5
1	0.75	0.43	0.4	1	0.33	1	1	1	0	0	0.5	1	0.25
1	0.5	0.14	0	1	0	1	0.17	0.5	0	0	0.5	0	0.38
1	0.75	0.43	0	1	0.33	1	0.83	1	0	0	0.5	0	0.38
1	0.75	0.43	0.2	1	0.33	1	1	1	0	0	0.5	0.67	0.25
1	0.75	0.43	0	1	0.33	1	1	1	0	0	0.5	1	0.13
0.5	0.75	0.43	0	1	0.67	1	0.83	1	0	0	0.5	1	0.38
1	0.75	0.57	0	1	0.33	1	0.83	1	0	0	0.5	0.67	0.25
1	1	0.43	0	1	0.67	1	1	1	0	0	0.5	1	0.5
1	0.75	0.43	0	1	0.67	1	1	1	0	0	0.5	1	0.75
0.5	0.75	0.43	0	1	0.33	1	0.83	1	0	0	0.5	1	0.38
1	0.75	0.43	0	1	0.33	1	0.83	1	0	0	0.5	0.67	0.5
0.5	0.75	0.43	0	1	0.33	1	1	1	0	0	0.5	0.67	0.25
0	0.75	0.14	0	1	0.33	0.5	1	0.5	0	0	0.5	0	0.13
1	0.75	0.43	0	1	0.33	1	0.83	1	0	0	0.5	1	0.63
0.5	0.75	0.29	0	1	0.33	1	1	1	0	0	0.5	1	0.38
0.5	0.75	0.29	0	0	0.33	1	0.67	1	0	0	0.5	0.33	0.5
0.5	0.75	0.14	0	1	0.33	1	0.83	1	0	0	0.5	1	0.63
0.5	0.75	0.57	0.8	1	0.67	1	0.83	1	0	0	0.5	0.67	0.38
0.5	0.75	0.14	0.4	1	0	1	0.83	1	0	0	0.5	0	0.38
1	0.75	0.43	0	1	0.33	1	1	1	0	0	0.5	0	0.63
0.5	0.75	0.57	0.4	1	0.33	1	1	1	0	1	0.5	1	0.25
0.5	0.75	0.43	0	1	0.33	1	0.83	1	0	0	0.5	1	0.38
1	0.75	0.57	0.4	1	0.33	1	1	1	0	0	0.5	0.67	0.25
0.5	0.75	0.43	0.4	1	0.33	1	1	1	0	0	0.5	0.67	0.25
0.5	0.75	0.43	0.4	1	0.33	1	1	1	0	0	0.5	0.67	0.38
1	0.75	0.43	0.4	1	0.33	1	1	1	0	0	0.5	0.67	0.25
1	0.5	0.43	0.2	1	0.33	0.5	1	1	0	0	0.5	0.33	0.25
0.5	1	0.43	0	1	0.33	1	0.17	0.5	0	0	0.5	0	0

0.5	0.75	0.43	0	1	0.33	1	0.17	0.5	0	0	0.5	0.67	0.13
0.5	0.25	0.57	0	1	0.33	1	0.17	0.5	1	0	0.5	0.33	0
0.5	0	0.43	0	1	0.33	0.5	0.17	0.33	0	0	0	0	0.13
0	1	0.43	0.2	1	1	1	1	1	0	0	0.5	0.67	0.25
1	1	0.43	0.2	1	0.67	0.5	1	1	0	0	0.5	0	0.38
1	1	0.43	0.2	1	0.33	1	1	1	0	0	0.5	0.33	0.5
0.5	1	0.57	0.4	0	0.67	0.5	0.67	0.67	0	0	0	0.67	0.38
0.5	1	0.43	0	1	1	1	1	1	0	0	0.5	0.67	0.25
1	1	0.43	0	1	0.33	1	1	1	0	0	0.5	0.67	0.38
1	0.5	0.43	0.2	1	0.67	0.5	1	1	0	0	0.5	0	0.5
1	1	0.43	0.2	1	0.67	0.5	1	1	0	0	0.5	0.33	0.5
0.5	1	0.43	0	1	1	1	1	1	0	0	0.5	0.33	0.13
0.5	1	0.43	0	1	1	1	1	1	0	0	0.5	0.67	0.13
1	1	0.43	0.2	1	0.67	0.5	1	1	0	0	0.5	0.33	0.5
0	0.5	0.14	0	1	0.67	1	0.67	0	0	0	0	0.33	0
0.5	0.5	0.29	0	1	0.67	0.5	0.17	0.67	1	0	0.5	0.33	0.13
0	0.25	0.14	0	1	0.33	1	1	1	0	0	0	1	0.13
0	0.25	0	0	1	0.33	1	1	1	0	0	0	1	0.38
0.5	0.5	0	0	1	0.33	0.5	0.83	0.5	0	0	0	0.33	0.13
0	0.25	0	0	1	0.33	0.5	1	1	0	0	0	0.33	0.25
0.5	0.5	0	0	1	0.67	0.5	1	1	0	0	0	1	0.38
0	0.25	0.14	0	1	0.33	1	1	1	0	0	0	0.33	0.13
0	0.5	0	0	0	0.33	0.5	0.83	0.5	0	0	0	0	0
0.5	0.5	0.14	0	1	0.33	1	1	1	0	0	0.5	1	0.13
0	0.25	0.14	0	1	0.33	1	0.67	1	0	0	0.5	0.33	0
0	0	0	0	1	0.33	1	1	0.5	0	0	0.5	1	0.25
0.5	0	0.14	0	1	0.33	1	1	1	0	0	0.5	1	0.38
0.5	0.25	0	0	1	0	0	0.83	0.5	0	0	0.5	1	0.13
0	0	0	0	1	0.33	1	0.83	1	0	0	0	0.33	0.38
0	0.75	0.14	0	1	0.67	0.5	0.17	1	0	0	0.5	0	0.13
0	0.75	0.14	0	1	0.33	1	0.17	1	0	0	0.5	0	0
0	0.75	0.14	0	1	0.67	1	0.17	1	0	0	0.5	0	0
0	0.75	0.14	0.2	1	0.67	1	0.17	1	0	0	0.5	0	0.13
0.5	1	0	0	1	0.33	1	0.17	1	0	0	0.5	0	0
0	0.75	0.29	0	1	0.33	1	0.17	1	0	0	0.5	0	0
0.5	0.75	0.14	0	1	0.67	1	0.17	1	0	0	0.5	0	0
0.5	0.5	0.14	0	1	0.33	1	0.17	1	0	0	0.5	0	0.25
0	0.5	0.14	0	1	0.67	1	0.17	1	0	0	0.5	0	0.13
0	0.25	0.14	0	0	0.33	0	0	0	0	0	0	0	0.38
1	0.75	0.43	0	1	1	1	1	1	0	0	0.5	1	0.13
0.5	0.75	0.14	0	1	0.67	1	0.17	0	0	0	0.5	0.67	0.38
0.5	0.75	0.14	0	1	0.33	1	1	1	1	0	0.5	0.67	0.38
0.5	0.75	0.29	0.4	1	1	1	0.5	0.5	0	0	0.5	0.67	0.13
1	0.75	0.14	0.4	1	0.67	1	0.17	0.5	1	0	0	1	0
1	1	0.86	0.4	1	0.67	1	0.33	0.5	0	0	0.5	0	0.25
0.5	0.75	0.43	0	1	0.33	1	0.17	0.67	1	0	0.5	0.67	0.25
0	1	0.29	0.4	1	0.67	1	0.17	0	0	0	0.5	0.67	0
1	1	0.14	0	1	0.33	1	1	1	0	0	0.5	0.67	0.25
1	0.25	0.29	0	1	0.67	0.5	0.17	0.33	1	0	0	1	0
1	0.25	0.43	0	1	0	1	0.5	0.5	1	0	0.5	0.33	0.38
0.5	0.5	0.29	0.4	1	0.67	1	0.5	0.33	1	0	0.5	0.33	0.25
0.5	1	0.14	0	1	0.33	1	0.67	1	1	0	0.5	0.67	0.25
0.5	1	0.57	0.4	1	0.67	1	1	1	1	0	0.5	0.67	0
1	1	0.57	0.4	1	0.33	1	1	0.5	1	0	0.5	0.67	0.13
1	1	0.57	0.4	1	0.67	1	1	1	1	0	0.5	0.67	0.25
0.5	1	0.14	0	1	0.33	0	0.67	0.33	1	0	0	0.67	0.38
0.5	0.75	0.14	0	1	0.33	1	1	1	1	0	0.5	0.33	0
1	0.5	0.29	0.4	1	0.33	1	1	1	0	0	0.5	0.33	0.25
0.5	0.5	0.57	0	1	1	1	1	1	0	0	0.5	0.67	0.88
0.5	1	0.71	0.4	1	0.67	0.5	1	1	0	0	0.5	1	0
0.5	1	0.71	0.4	1	0.67	0.5	1	1	0	0	0.5	0.67	0.13
0.5	1	0.71	0.4	1	1	1	1	1	0	0	0.5	1	0.13
