# Supplementary material for: Vitamin and Amino Acid Auxotrophy in Anaerobic Consortia Operating under Methanogenic Conditions
Source: mSystems. 2017 Oct 31;2(5):e00038-17. doi: 10.1128/mSystems.00038-17 (PMC5663940; doi:10.1128/mSystems.00038-17)
Supplement: TABLE S4 [file sys005172144st5.pdf]

sample	description	sampling date	genome equivalent	number of QC nucleotids [Mb]
SRR634694*	full community shot gun metagenome after four transfers	21-May-2013	338.4	
SRR634695*	full community shot gun metagenome after four transfers	21-May-2013	210.0	
SRR636559	full community shot gun metagenome after four transfers	22-May-2013	15741.1	
ERS1696413	after seven transfers; full sample (no MDA)	20-Jan-2014	270.2	15098302
ERS1696414	after seven transfers; full sample (MDA)	20-Jan-2014	235.9	34245392
ERS1696415	after seven transfers; + Epibiont	13-Aug-2013	381.9	34588472
ERS1696416	after seven transfers; - Epibiont	13-Aug-2013	9.4	30221348
ERS1696417	after seven transfers; - Epibiont	13-Aug-2013	516.8	28750174
ERS1696418	after seven transfers; - Epibiont	13-Aug-2013	250.3	32807706
ERS1696419	after seven transfers; + Epibiont	10-Jul-2013	24.0	30397434
ERS1696420	after seven transfers; + Epibiont	10-Jul-2013	24.2	30871468
ERS1696421	after seven transfers; + Epibiont	21-Jul-2013	130.2	27302066
ERS1696422	after seven transfers; + Epibiont	21-Jul-2013	222.4	31446834

* published previously (Tan B, Dong X, Sensen CW, Foght J. Metagenomic analysis of an anaerobic alkane-degrading microbial culture: potential hydrocarbon-activating pathways and inferred roles of community members. Genome / National Research Council Canada = Genome / Conseil national de recherches Canada. 2013;56:599-611.)
