# Supplementary figures and images for: Vitamin and Amino Acid Auxotrophy in Anaerobic Consortia Operating under Methanogenic Conditions
Source: mSystems. 2017 Oct 31;2(5):e00038-17. doi: 10.1128/mSystems.00038-17 (PMC5663940; doi:10.1128/mSystems.00038-17)

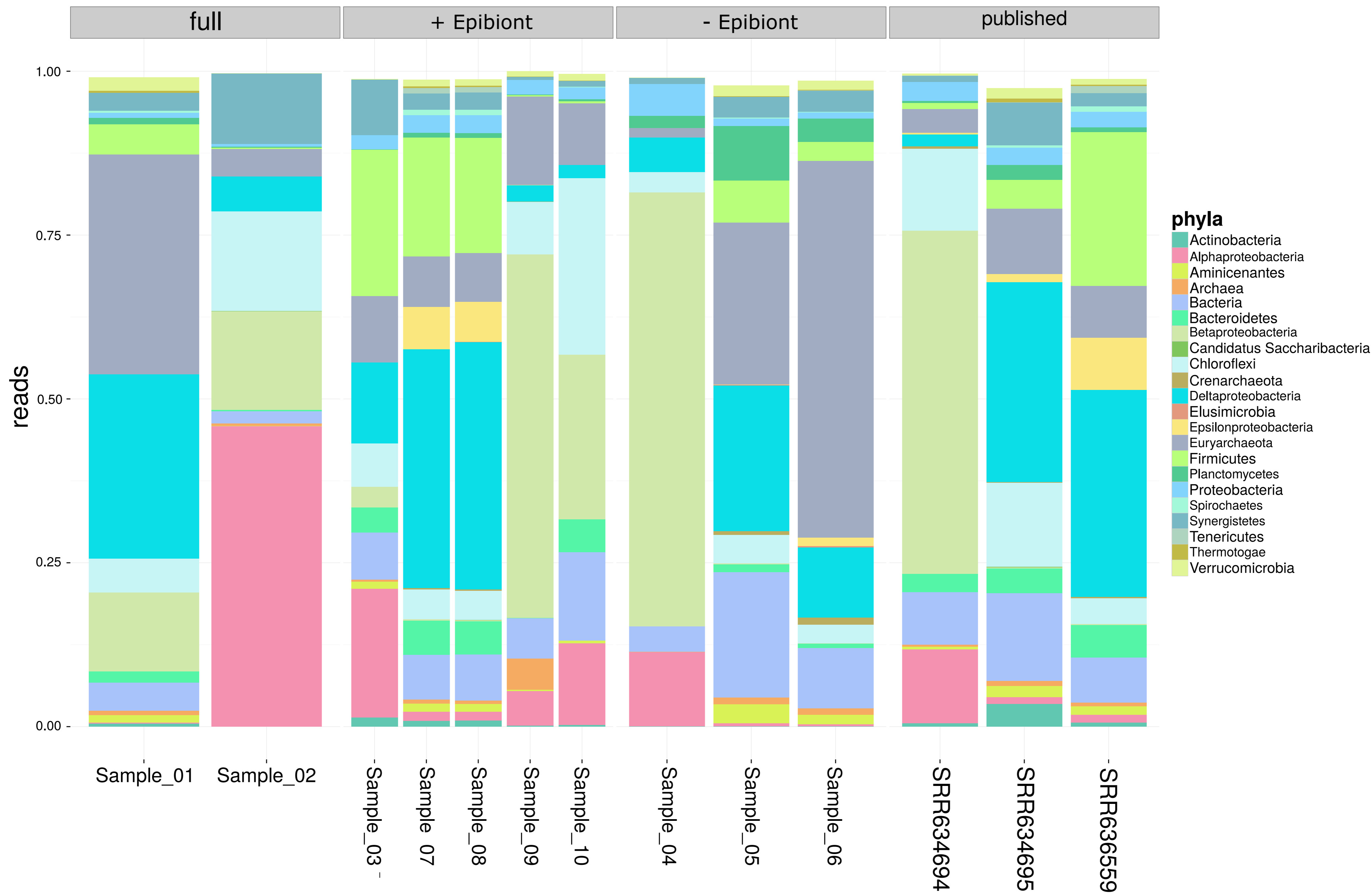

Supplement: FIG S1 [file sys005172144sf1.pdf]
