# Supplementary material for: Vitamin and Amino Acid Auxotrophy in Anaerobic Consortia Operating under Methanogenic Conditions
Source: mSystems. 2017 Oct 31;2(5):e00038-17. doi: 10.1128/mSystems.00038-17 (PMC5663940; doi:10.1128/mSystems.00038-17)
Supplement: TABLE S5 [file sys005172144st6.pdf]

Bin Id	Marker lineage	# genomes	# markers	# marker sets
scadc_bin_184	k__Bacteria (UID2328)	3167	126	75
scadc_bin_010	p__Proteobacteria (UID3061)	115	395	260
scadc_bin_165	k__Bacteria (UID203)	5449	104	58
scadc_bin_264	k__Bacteria (UID203)	5449	103	57
scadc_bin_142	k__Bacteria (UID203)	5449	103	57
scadc_bin_250	k__Bacteria (UID203)	5449	103	57
scadc_bin_200	o__Clostridiales (UID1120)	304	247	141
scadc_bin_033	k__Bacteria (UID2570)	433	273	183
scadc_bin_005	o__Clostridiales (UID1212)	172	263	149
scadc_bin_206	p__Bacteroidetes (UID2605)	350	316	210
scadc_bin_073	k__Bacteria (UID203)	5449	103	57
scadc_bin_092	k__Bacteria (UID203)	5449	104	58
scadc_bin_034	c__Deltaproteobacteria (UID3218)	61	284	169
scadc_bin_096	k__Bacteria (UID2569)	434	278	186
scadc_bin_110	k__Bacteria (UID203)	5449	104	58
scadc_bin_049	p__Euryarchaeota (UID54)	90	234	153
scadc_bin_023	c__Deltaproteobacteria (UID3216)	83	246	155
scadc_bin_064	k__Bacteria (UID2569)	434	278	186
scadc_bin_009	k__Bacteria (UID203)	5449	104	58
scadc_bin_056	k__Bacteria (UID203)	5449	103	57
scadc_bin_157	o__Clostridiales (UID1120)	304	250	143
scadc_bin_151	p__Bacteroidetes (UID2605)	350	316	210
scadc_bin_192	p__Euryarchaeota (UID54)	90	234	153
scadc_bin_158	p__Firmicutes (UID1022)	100	295	158
scadc_bin_195	k__Bacteria (UID2570)	433	273	183
scadc_bin_036	k__Bacteria (UID203)	5449	103	57
scadc_bin_224	k__Bacteria (UID209)	5443	105	59
scadc_bin_018	k__Bacteria (UID203)	5449	103	57
scadc_bin_187	k__Bacteria (UID203)	5449	104	58
scadc_bin_083	k__Bacteria (UID1452)	924	151	101
scadc_bin_129	c__Deltaproteobacteria (UID3216)	83	247	155
scadc_bin_006	c__Deltaproteobacteria (UID3216)	83	247	155
scadc_bin_039	o__Clostridiales (UID1212)	172	263	149
scadc_bin_072	p__Euryarchaeota (UID54)	90	234	153
scadc_bin_126	k__Bacteria (UID2569)	434	278	186
scadc_bin_113	c__Deltaproteobacteria (UID3216)	83	247	155
scadc_bin_053	k__Bacteria (UID2570)	433	273	183
scadc_bin_050	k__Bacteria (UID203)	5449	104	58
scadc_bin_032	k__Bacteria (UID203)	5449	104	58
scadc_bin_001	k__Bacteria (UID203)	5449	104	58
scadc_bin_148	c__Deltaproteobacteria (UID3216)	83	247	155
scadc_bin_221	c__Deltaproteobacteria (UID3216)	83	247	155
scadc_bin_133	k__Bacteria (UID1452)	924	151	101
scadc_bin_154	k__Bacteria (UID2495)	2993	141	87
scadc_bin_127	k__Bacteria (UID2495)	2993	141	87
scadc_bin_091	c__Clostridia (UID1085)	35	420	196
scadc_bin_029	k__Bacteria (UID209)	5443	105	59
scadc_bin_099	c__Clostridia (UID1085)	35	420	196
scadc_bin_228	k__Bacteria (UID203)	5449	104	58
scadc_bin_020	k__Bacteria (UID2495)	2993	142	88
scadc_bin_002	k__Bacteria (UID2495)	2993	141	87
scadc_bin_042	p__Bacteroidetes (UID2605)	350	316	210
scadc_bin_097	k__Bacteria (UID203)	5449	104	58
scadc_bin_218	k__Bacteria (UID203)	5449	104	58
scadc_bin_199	k__Bacteria (UID203)	5449	104	58
scadc_bin_172	p__Euryarchaeota (UID49)	95	227	152

scadc_bin_017	p__Firmicutes (UID1022)	100	295	158
scadc_bin_122	c__Clostridia (UID1085)	35	420	196
scadc_bin_163	c__Deltaproteobacteria (UID3216)	83	247	155
scadc_bin_079	c__Clostridia (UID1085)	35	420	196
scadc_bin_013	c__Deltaproteobacteria (UID3216)	83	247	155
scadc_bin_144	k__Bacteria (UID2565)	2921	152	93
scadc_bin_225	k__Bacteria (UID2495)	2993	147	91
scadc_bin_088	c__Deltaproteobacteria (UID3216)	83	247	155
scadc_bin_147	k__Bacteria (UID1452)	924	163	110
scadc_bin_235	k__Bacteria (UID203)	5449	103	57
scadc_bin_084	f__Rhizobiaceae (UID3564)	78	840	354
scadc_bin_065	c__Deltaproteobacteria (UID3216)	83	247	155
scadc_bin_069	k__Bacteria (UID203)	5449	104	58
scadc_bin_254	k__Bacteria (UID1452)	924	163	110
scadc_bin_226	k__Bacteria (UID203)	5449	104	58
scadc_bin_128	c__Deltaproteobacteria (UID3216)	83	247	155
scadc_bin_100	p__Euryarchaeota (UID4)	45	299	202
scadc_bin_131	k__Bacteria (UID203)	5449	101	57
scadc_bin_239	k__Bacteria (UID1452)	924	163	110
scadc_bin_248	k__Bacteria (UID2495)	2993	142	88
scadc_bin_153	k__Bacteria (UID2495)	2993	143	89
scadc_bin_101	k__Bacteria (UID1452)	924	163	110
scadc_bin_121	k__Bacteria (UID203)	5449	103	57
scadc_bin_063	k__Bacteria (UID2495)	2993	141	87
scadc_bin_211	c__Clostridia (UID1085)	35	420	196
scadc_bin_019	p__Euryarchaeota (UID54)	90	234	153
scadc_bin_070	o__Clostridiales (UID1375)	50	332	124
scadc_bin_255	k__Bacteria (UID203)	5449	104	58
scadc_bin_263	c__Spirochaetia (UID2496)	72	215	125
scadc_bin_087	k__Bacteria (UID203)	5449	104	58
scadc_bin_270	k__Bacteria (UID209)	5443	105	59
scadc_bin_024	p__Bacteroidetes (UID2605)	350	316	210
scadc_bin_156	c__Deltaproteobacteria (UID3216)	83	247	155
scadc_bin_112	k__Bacteria (UID203)	5449	104	58
scadc_bin_059	k__Bacteria (UID203)	5449	104	58
scadc_bin_232	k__Bacteria (UID2570)	433	273	183
scadc_bin_252	k__Bacteria (UID203)	5449	104	58
scadc_bin_021	k__Bacteria (UID1452)	924	163	110
scadc_bin_137	c__Deltaproteobacteria (UID3216)	83	247	155
scadc_bin_205	k__Bacteria (UID203)	5449	104	58
scadc_bin_191	k__Bacteria (UID1452)	924	163	110
scadc_bin_189	o__Clostridiales (UID1120)	304	250	143
scadc_bin_086	c__Deltaproteobacteria (UID3217)	62	280	168
scadc_bin_272	c__Clostridia (UID1085)	35	420	196
scadc_bin_075	k__Bacteria (UID2495)	2993	143	89
scadc_bin_181	k__Bacteria (UID1452)	924	163	110
scadc_bin_230	k__Bacteria (UID203)	5449	103	57
scadc_bin_111	k__Bacteria (UID203)	5449	104	58
scadc_bin_182	k__Bacteria (UID203)	5449	103	57
scadc_bin_296	k__Bacteria (UID203)	5449	104	58
scadc_bin_196	o__Clostridiales (UID1120)	304	247	141
scadc_bin_277	k__Bacteria (UID209)	5443	105	59
scadc_bin_136	c__Deltaproteobacteria (UID3216)	83	247	155
scadc_bin_058	f__Burkholderiaceae (UID4003)	91	596	218
scadc_bin_004	k__Bacteria (UID203)	5449	103	57
scadc_bin_238	k__Bacteria (UID1452)	924	163	110
scadc_bin_125	k__Bacteria (UID3187)	2258	190	119

scadc_bin_185	c__Deltaproteobacteria (UID3216)	83	247	155
scadc_bin_152	k__Bacteria (UID203)	5449	104	58
scadc_bin_008	p__Euryarchaeota (UID54)	90	234	153
scadc_bin_209	c__Deltaproteobacteria (UID3217)	62	280	168
scadc_bin_212	c__Deltaproteobacteria (UID3216)	83	247	155
scadc_bin_078	k__Bacteria (UID1452)	924	163	110
scadc_bin_177	k__Bacteria (UID209)	5443	105	59
scadc_bin_276	o__Actinomycetales (UID1697)	387	330	193
scadc_bin_303	k__Bacteria (UID209)	5443	105	59
scadc_bin_282	p__Euryarchaeota (UID3)	148	187	124
scadc_bin_171	c__Clostridia (UID1085)	35	420	196
scadc_bin_246	k__Bacteria (UID209)	5443	105	59
scadc_bin_279	k__Bacteria (UID2329)	174	149	89
scadc_bin_081	k__Bacteria (UID203)	5449	103	57
scadc_bin_138	k__Bacteria (UID209)	5443	105	59
scadc_bin_275	k__Bacteria (UID203)	5449	104	58
scadc_bin_291	k__Bacteria (UID2982)	88	230	148
scadc_bin_203	c__Deltaproteobacteria (UID3216)	83	247	155
scadc_bin_262	o__Clostridiales (UID1120)	304	247	141
scadc_bin_150	k__Bacteria (UID1452)	924	163	110
scadc_bin_003	k__Bacteria (UID1452)	924	151	101
scadc_bin_140	k__Bacteria (UID203)	5449	104	58
scadc_bin_198	k__Bacteria (UID1452)	924	163	110
scadc_bin_290	k__Bacteria (UID2569)	434	278	186
scadc_bin_240	k__Bacteria (UID203)	5449	104	58
scadc_bin_281	k__Bacteria (UID203)	5449	103	57
scadc_bin_267	p__Euryarchaeota (UID54)	90	234	153
scadc_bin_169	k__Bacteria (UID203)	5449	104	58
scadc_bin_216	k__Bacteria (UID1452)	924	151	101
scadc_bin_294	k__Bacteria (UID1452)	924	163	110
scadc_bin_207	p__Firmicutes (UID239)	1324	173	101
scadc_bin_292	k__Bacteria (UID2495)	2993	147	91
scadc_bin_257	k__Bacteria (UID203)	5449	104	58
scadc_bin_054	k__Bacteria (UID203)	5449	104	58
scadc_bin_168	k__Bacteria (UID203)	5449	103	58
scadc_bin_174	c__Clostridia (UID1085)	35	420	196
scadc_bin_060	k__Bacteria (UID203)	5449	104	58
scadc_bin_044	k__Bacteria (UID2328)	3167	126	75
scadc_bin_273	k__Bacteria (UID203)	5449	104	58
scadc_bin_231	k__Bacteria (UID203)	5449	104	58
scadc_bin_301	k__Bacteria (UID203)	5449	104	58
scadc_bin_268	k__Bacteria (UID1452)	924	163	110
scadc_bin_299	c__Deltaproteobacteria (UID3216)	83	247	155
scadc_bin_089	k__Bacteria (UID203)	5449	104	58
scadc_bin_117	k__Bacteria (UID3187)	2258	188	117
scadc_bin_220	k__Bacteria (UID209)	5443	105	59
scadc_bin_271	k__Bacteria (UID1452)	924	163	110
scadc_bin_035	p__Euryarchaeota (UID54)	90	234	153
scadc_bin_223	k__Bacteria (UID203)	5449	104	58
scadc_bin_041	p__Euryarchaeota (UID54)	90	234	153
scadc_bin_159	k__Bacteria (UID203)	5449	104	58
scadc_bin_107	k__Bacteria (UID203)	5449	104	58
scadc_bin_251	k__Bacteria (UID203)	5449	104	58
scadc_bin_176	k__Bacteria (UID203)	5449	104	58
scadc_bin_106	k__Bacteria (UID203)	5449	104	58
scadc_bin_193	k__Bacteria (UID203)	5449	104	58
scadc_bin_265	p__Proteobacteria (UID3887)	1487	259	162

scadc_bin_066	k__Bacteria (UID203)	5449	103	57
scadc_bin_245	k__Bacteria (UID203)	5449	104	58
scadc_bin_302	k__Bacteria (UID203)	5449	104	58
scadc_bin_124	c__Deltaproteobacteria (UID3216)	83	247	155
scadc_bin_210	root (UID1)	5656	56	24
scadc_bin_307	k__Bacteria (UID2565)	2921	152	93
scadc_bin_186	k__Bacteria (UID1452)	924	163	110
scadc_bin_046	k__Archaea (UID2)	207	149	107
scadc_bin_155	k__Bacteria (UID203)	5449	104	58
scadc_bin_280	k__Bacteria (UID1452)	924	151	101
scadc_bin_293	p__Actinobacteria (UID1454)	732	199	116
scadc_bin_260	k__Bacteria (UID203)	5449	103	57
scadc_bin_242	k__Bacteria (UID3187)	2258	188	117
scadc_bin_028	o__Clostridiales (UID1120)	304	250	143
scadc_bin_283	k__Bacteria (UID1452)	924	163	110
scadc_bin_234	k__Bacteria (UID203)	5449	104	58
scadc_bin_297	k__Bacteria (UID1452)	924	151	101
scadc_bin_256	k__Bacteria (UID3187)	2258	190	119
scadc_bin_304	k__Bacteria (UID203)	5449	104	58
scadc_bin_014	k__Bacteria (UID203)	5449	103	57
scadc_bin_300	k__Bacteria (UID209)	5443	105	59
scadc_bin_132	k__Bacteria (UID203)	5449	104	58
scadc_bin_236	k__Bacteria (UID1452)	924	163	110
scadc_bin_258	k__Bacteria (UID203)	5449	104	58
scadc_bin_037	c__Deltaproteobacteria (UID3216)	83	247	155
scadc_bin_289	k__Bacteria (UID203)	5449	104	58
scadc_bin_045	p__Euryarchaeota (UID54)	90	234	153
scadc_bin_214	k__Bacteria (UID203)	5449	104	58
scadc_bin_261	p__Actinobacteria (UID1454)	732	206	120
scadc_bin_118	k__Bacteria (UID203)	5449	101	57
scadc_bin_077	k__Bacteria (UID203)	5449	103	57
scadc_bin_202	k__Bacteria (UID209)	5443	105	59
scadc_bin_108	k__Bacteria (UID203)	5449	104	58
scadc_bin_217	k__Bacteria (UID203)	5449	104	58
scadc_bin_141	k__Bacteria (UID1452)	924	163	110
scadc_bin_012	k__Archaea (UID2)	207	149	107
scadc_bin_287	k__Bacteria (UID203)	5449	104	58
scadc_bin_286	k__Bacteria (UID203)	5449	104	58
scadc_bin_130	o__Clostridiales (UID1120)	304	250	143
scadc_bin_306	k__Bacteria (UID203)	5449	104	58
scadc_bin_052	k__Bacteria (UID209)	5443	105	59
scadc_bin_233	k__Bacteria (UID203)	5449	104	58
scadc_bin_188	c__Deltaproteobacteria (UID3216)	83	247	155
scadc_bin_229	k__Bacteria (UID203)	5449	104	58
scadc_bin_067	k__Bacteria (UID203)	5449	104	58
scadc_bin_237	k__Bacteria (UID203)	5449	104	58
scadc_bin_215	k__Bacteria (UID203)	5449	104	58
scadc_bin_288	p__Firmicutes (UID1022)	100	295	158
scadc_bin_285	k__Bacteria (UID203)	5449	104	58
scadc_bin_183	k__Bacteria (UID203)	5449	104	58
scadc_bin_204	k__Bacteria (UID2329)	174	149	89
scadc_bin_305	k__Bacteria (UID203)	5449	104	58
scadc_bin_027	k__Archaea (UID2)	207	149	107
scadc_bin_175	k__Bacteria (UID1452)	924	163	110
scadc_bin_266	k__Bacteria (UID1452)	924	160	109
scadc_bin_219	k__Bacteria (UID203)	5449	104	58
scadc_bin_227	k__Bacteria (UID203)	5449	104	58

scadc_bin_160	k__Bacteria (UID203)	5449	103	57
scadc_bin_180	c__Deltaproteobacteria (UID3217)	62	280	168
scadc_bin_011	k__Archaea (UID2)	207	149	107
scadc_bin_269	k__Bacteria (UID203)	5449	104	58
scadc_bin_095	k__Bacteria (UID203)	5449	103	57
scadc_bin_115	k__Bacteria (UID203)	5449	104	58
scadc_bin_166	k__Archaea (UID2)	207	149	107
scadc_bin_146	k__Bacteria (UID203)	5449	102	56
scadc_bin_098	p__Euryarchaeota (UID49)	95	228	153
scadc_bin_026	k__Bacteria (UID203)	5449	104	58
scadc_bin_222	k__Bacteria (UID203)	5449	104	58
scadc_bin_278	k__Bacteria (UID203)	5449	104	58
scadc_bin_134	c__Spirochaetia (UID2496)	72	215	125
scadc_bin_249	k__Bacteria (UID203)	5449	103	57
scadc_bin_145	k__Bacteria (UID203)	5449	101	57
scadc_bin_208	k__Bacteria (UID203)	5449	104	58
scadc_bin_161	k__Bacteria (UID203)	5449	104	58
scadc_bin_030	k__Bacteria (UID203)	5449	104	58
scadc_bin_016	k__Bacteria (UID203)	5449	104	58
scadc_bin_022	k__Bacteria (UID203)	5449	104	58
scadc_bin_104	k__Bacteria (UID203)	5449	104	58
scadc_bin_048	c__Deltaproteobacteria (UID3216)	83	247	155
scadc_bin_119	k__Bacteria (UID203)	5449	104	58
scadc_bin_139	k__Archaea (UID2)	207	149	107
scadc_bin_080	k__Bacteria (UID203)	5449	104	58
scadc_bin_194	k__Archaea (UID2)	207	149	107
scadc_bin_178	root (UID1)	5656	56	24
scadc_bin_167	k__Bacteria (UID2495)	2993	142	88
scadc_bin_123	root (UID1)	5656	56	24
scadc_bin_082	root (UID1)	5656	56	24
scadc_bin_061	root (UID1)	5656	56	24
scadc_bin_143	k__Bacteria (UID203)	5449	104	58
scadc_bin_043	k__Bacteria (UID203)	5449	104	58
scadc_bin_284	k__Bacteria (UID203)	5449	102	56
scadc_bin_120	k__Bacteria (UID203)	5449	104	58
scadc_bin_295	k__Archaea (UID2)	207	149	107
scadc_bin_090	k__Bacteria (UID203)	5449	102	56
scadc_bin_164	root (UID1)	5656	56	24
scadc_bin_057	k__Archaea (UID2)	207	149	107
scadc_bin_274	k__Bacteria (UID203)	5449	104	58
scadc_bin_298	k__Bacteria (UID203)	5449	104	58
scadc_bin_105	k__Bacteria (UID203)	5449	104	58
scadc_bin_170	k__Bacteria (UID203)	5449	104	58
scadc_bin_103	k__Bacteria (UID203)	5449	103	57
scadc_bin_094	k__Bacteria (UID203)	5449	103	57
scadc_bin_197	o__Clostridiales (UID1120)	304	247	141
scadc_bin_025	k__Archaea (UID2)	207	148	106
scadc_bin_190	k__Archaea (UID2)	207	149	107
scadc_bin_173	k__Bacteria (UID203)	5449	104	58
scadc_bin_047	k__Bacteria (UID203)	5449	104	58
scadc_bin_031	root (UID1)	5656	56	24
scadc_bin_244	k__Archaea (UID2)	207	149	107
scadc_bin_162	c__Deltaproteobacteria (UID3217)	62	280	168
scadc_bin_253	k__Bacteria (UID203)	5449	104	58
scadc_bin_213	root (UID1)	5656	56	24
scadc_bin_247	k__Bacteria (UID203)	5449	104	58
scadc_bin_109	k__Bacteria (UID203)	5449	104	58

scadc_bin_259	root (UID1)	5656	56	24
scadc_bin_243	root (UID1)	5656	56	24
scadc_bin_241	root (UID1)	5656	56	24
scadc_bin_201	root (UID1)	5656	56	24
scadc_bin_179	root (UID1)	5656	56	24
scadc_bin_149	root (UID1)	5656	56	24
scadc_bin_135	root (UID1)	5656	56	24
scadc_bin_116	root (UID1)	5656	56	24
scadc_bin_114	root (UID1)	5656	56	24
scadc_bin_102	root (UID1)	5656	56	24
scadc_bin_093	root (UID1)	5656	56	24
scadc_bin_085	root (UID1)	5656	56	24
scadc_bin_076	root (UID1)	5656	56	24
scadc_bin_074	root (UID1)	5656	56	24
scadc_bin_071	root (UID1)	5656	56	24
scadc_bin_068	root (UID1)	5656	56	24
scadc_bin_062	root (UID1)	5656	56	24
scadc_bin_055	root (UID1)	5656	56	24
scadc_bin_051	root (UID1)	5656	56	24
scadc_bin_040	root (UID1)	5656	56	24
scadc_bin_038	root (UID1)	5656	56	24
scadc_bin_015	root (UID1)	5656	56	24
scadc_bin_007	root (UID1)	5656	56	24
--------------------	-----------------------------------	------------	------------	----------------

0	1	2	3	4	5+	Completeness	Contamination	Strain heterogeneity
0	105	18	3	0	0	100.00	20.83	0.00
0	382	13	0	0	0	100.00	2.83	0.00
1	14	78	9	2	0	99.66	103.70	58.12
1	4	9	6	16	67	99.12	403.10	0.68
1	10	89	3	0	0	99.12	89.11	28.57
2	51	34	10	5	1	98.96	63.16	0.00
2	201	39	3	2	0	98.94	17.89	1.67
2	260	10	1	0	0	98.91	5.92	0.00
2	258	3	0	0	0	98.66	1.57	0.00
4	259	50	3	0	0	98.33	21.43	1.69
1	87	15	0	0	0	98.25	2.39	0.00
2	9	29	57	7	0	98.12	150.30	25.62
11	268	5	0	0	0	97.70	2.66	0.00
7	260	11	0	0	0	97.31	4.57	0.00
3	39	54	8	0	0	97.26	84.45	50.00
6	224	4	0	0	0	97.01	1.99	0.00
25	210	11	0	0	0	96.85	2.88	18.18
9	256	13	0	0	0	96.77	6.18	0.00
2	65	29	8	0	0	96.55	27.90	18.87
2	4	38	56	2	1	96.49	149.84	29.82
9	238	3	0	0	0	96.36	2.10	0.00
8	301	7	0	0	0	96.19	1.48	0.00
34	94	90	15	1	0	95.70	66.51	17.02
10	285	0	0	0	0	95.25	0.00	0.00
10	248	15	0	0	0	95.25	5.68	13.33
11	19	70	3	0	0	95.22	88.79	73.42
3	98	4	0	0	0	94.92	4.39	0.00
3	55	45	0	0	0	94.74	45.96	35.56
4	36	34	26	4	0	94.67	91.50	27.21
8	141	2	0	0	0	94.22	1.98	0.00
9	223	15	0	0	0	94.19	7.57	0.00
12	225	10	0	0	0	94.11	3.34	0.00
13	249	1	0	0	0	93.96	0.67	0.00
13	206	15	0	0	0	93.90	3.12	46.67
12	238	26	2	0	0	93.82	12.15	0.00
12	188	44	3	0	0	93.55	23.85	7.55
21	220	32	0	0	0	93.16	15.21	34.38
7	28	45	15	8	1	93.10	134.17	21.57
4	20	67	12	1	0	93.10	73.77	74.31
4	75	24	1	0	0	93.10	14.89	7.41
12	200	35	0	0	0	92.90	13.66	0.00
17	190	33	6	1	0	92.42	20.03	1.75
9	138	4	0	0	0	92.41	2.38	0.00
13	125	3	0	0	0	91.97	1.36	0.00
7	127	7	0	0	0	91.95	6.13	42.86
25	392	3	0	0	0	91.67	0.34	0.00
5	85	15	0	0	0	91.53	15.18	26.67
33	380	7	0	0	0	91.45	2.11	0.00
6	4	5	30	30	29	91.38	255.83	19.97
8	133	1	0	0	0	90.91	1.14	0.00
10	115	16	0	0	0	90.23	12.07	25.00
30	241	42	3	0	0	90.12	13.90	21.57
30	55	19	0	0	0	89.66	8.62	5.26
7	60	21	11	5	0	89.50	33.10	1.19
7	39	41	12	5	0	89.50	63.41	0.93
22	200	5	0	0	0	89.39	2.30	20.00

29	242	24	0	0	0	89.31	8.24	4.17
45	370	5	0	0	0	88.10	1.38	0.00
22	225	0	0	0	0	88.03	0.00	0.00
46	361	11	2	0	0	87.92	3.47	5.88
49	146	48	4	0	0	87.85	25.22	43.33
12	137	3	0	0	0	87.63	2.15	0.00
15	119	10	3	0	0	87.55	12.53	0.00
22	217	8	0	0	0	87.10	5.16	12.50
17	94	45	5	2	0	87.02	43.12	13.89
10	48	38	7	0	0	86.52	39.14	1.69
145	695	0	0	0	0	86.32	0.00	0.00
31	193	22	1	0	0	86.30	8.99	36.00
8	67	22	7	0	0	86.21	19.00	2.33
19	86	40	15	2	1	85.45	61.66	3.74
10	53	25	7	5	4	85.34	93.57	5.76
29	191	22	5	0	0	84.78	15.83	5.41
43	235	20	0	1	0	84.73	10.14	34.62
44	19	34	4	0	0	84.37	63.16	28.26
20	101	35	7	0	0	84.00	31.79	10.71
55	81	6	0	0	0	83.52	6.82	0.00
18	122	3	0	0	0	83.15	2.81	0.00
20	119	22	2	0	0	82.73	14.90	32.14
13	15	71	4	0	0	82.14	73.02	55.42
22	109	10	0	0	0	82.08	5.12	10.00
77	302	37	1	1	2	81.92	13.35	0.00
42	191	1	0	0	0	80.72	0.05	0.00
66	106	134	22	4	0	80.40	72.15	71.43
25	49	21	9	0	0	80.33	33.86	2.08
43	168	4	0	0	0	80.06	3.20	25.00
15	7	57	25	0	0	79.86	95.61	16.67
22	70	13	0	0	0	79.43	13.36	23.08
51	254	11	0	0	0	78.89	3.83	45.45
45	169	31	2	0	0	78.76	15.64	0.00
15	17	65	7	0	0	77.43	58.39	11.63
14	90	0	0	0	0	77.01	0.00	0.00
79	164	26	3	1	0	76.81	16.39	4.88
18	37	46	2	1	0	76.07	47.96	18.97
31	107	21	2	2	0	75.96	21.86	5.13
52	177	18	0	0	0	75.91	4.48	44.44
53	47	4	0	0	0	75.86	6.03	0.00
37	118	7	1	0	0	75.76	6.21	0.00
73	125	43	6	3	0	75.65	30.03	0.00
58	205	17	0	0	0	75.57	3.42	76.47
110	199	90	17	3	1	75.43	30.66	0.59
27	115	1	0	0	0	75.18	1.12	0.00
32	127	4	0	0	0	75.00	3.64	0.00
17	23	46	17	0	0	74.40	66.36	43.30
18	65	21	0	0	0	73.98	28.45	33.33
54	42	7	0	0	0	73.68	11.40	0.00
25	43	26	9	0	1	73.59	50.29	0.00
60	186	1	0	0	0	73.40	0.02	0.00
43	53	8	1	0	0	73.04	10.63	0.00
50	181	15	1	0	0	72.90	4.21	5.56
171	423	2	0	0	0	71.40	0.57	0.00
17	35	43	8	0	0	71.05	43.14	34.33
48	101	12	1	1	0	71.04	10.06	0.00
50	128	12	0	0	0	70.75	6.49	33.33

60	172	15	0	0	0	70.59	5.52	0.00
22	55	26	1	0	0	69.67	18.53	37.93
81	106	41	5	1	0	69.20	24.04	4.84
71	191	18	0	0	0	68.73	7.04	0.00
93	129	24	1	0	0	68.21	12.04	11.11
39	112	12	0	0	0	68.18	8.03	41.67
55	47	3	0	0	0	68.10	5.08	0.00
109	211	10	0	0	0	67.47	3.02	0.00
31	47	21	3	3	0	67.26	29.92	0.00
58	123	5	0	1	0	66.29	3.69	45.45
134	267	17	2	0	0	66.05	4.88	8.70
47	36	17	5	0	0	66.02	38.98	6.25
43	104	2	0	0	0	65.15	1.69	0.00
60	37	6	0	0	0	64.91	8.77	0.00
43	45	13	4	0	0	64.38	22.65	4.00
24	34	36	10	0	0	64.34	31.86	6.06
81	107	30	9	3	0	63.76	22.79	0.00
82	160	5	0	0	0	63.58	2.90	20.00
115	125	6	1	0	0	63.36	4.31	11.11
74	85	4	0	0	0	62.61	3.64	0.00
44	101	5	1	0	0	62.18	5.61	25.00
27	69	8	0	0	0	61.05	5.99	25.00
72	68	20	3	0	0	60.66	17.82	27.59
102	174	2	0	0	0	58.06	1.08	0.00
51	32	17	4	0	0	55.83	15.00	0.00
40	38	25	0	0	0	54.86	16.06	0.00
104	114	14	2	0	0	54.40	10.51	10.00
46	37	12	3	2	4	54.36	17.24	0.00
55	80	16	0	0	0	53.76	10.73	0.00
71	68	16	7	1	0	52.64	23.06	13.95
61	82	22	7	1	0	51.75	26.97	12.24
66	80	1	0	0	0	51.73	1.10	0.00
63	30	9	2	0	0	51.44	13.56	33.33
46	50	7	1	0	0	50.68	10.34	0.00
68	35	0	0	0	0	50.31	0.00	0.00
207	201	10	2	0	0	50.26	3.57	0.00
34	45	25	0	0	0	50.00	17.24	4.00
60	65	1	0	0	0	49.75	1.33	0.00
73	29	2	0	0	0	46.55	2.59	50.00
34	56	14	0	0	0	46.39	7.84	0.00
43	38	16	5	2	0	45.30	13.82	0.00
94	53	10	6	0	0	44.56	17.01	7.14
125	118	4	0	0	0	43.51	1.94	25.00
43	51	10	0	0	0	43.10	4.70	0.00
112	75	1	0	0	0	42.64	0.43	0.00
61	30	8	5	1	0	42.01	17.18	3.45
86	65	10	2	0	0	41.28	7.01	0.00
146	78	10	0	0	0	40.58	4.02	60.00
73	26	2	3	0	0	40.44	13.79	0.00
141	79	14	0	0	0	40.30	6.81	78.57
73	29	2	0	0	0	39.26	2.59	0.00
71	25	7	1	0	0	39.04	9.09	0.00
73	30	1	0	0	0	39.03	1.72	0.00
42	31	25	6	0	0	37.93	14.32	0.00
70	33	1	0	0	0	37.67	1.72	0.00
71	26	7	0	0	0	37.54	6.21	42.86
143	100	14	2	0	0	37.53	6.29	0.00

67	29	7	0	0	0	37.05	8.77	14.29
76	28	0	0	0	0	36.99	0.00	0.00
74	26	4	0	0	0	36.90	5.17	0.00
145	87	15	0	0	0	35.76	5.81	33.33
46	6	1	3	0	0	35.42	29.17	20.00
94	45	11	2	0	0	35.38	6.01	0.00
110	53	0	0	0	0	35.00	0.00	0.00
102	44	3	0	0	0	34.74	1.87	100.00
81	21	2	0	0	0	34.48	1.72	50.00
95	49	6	1	0	0	33.93	7.43	0.00
119	67	11	1	1	0	33.51	4.73	0.00
79	21	3	0	0	0	33.49	5.26	0.00
126	47	15	0	0	0	33.44	8.55	6.67
154	86	9	1	0	0	33.28	4.66	0.00
105	45	10	3	0	0	32.63	8.09	0.00
80	24	0	0	0	0	32.37	0.00	0.00
91	55	5	0	0	0	31.57	3.96	0.00
122	58	10	0	0	0	31.48	7.07	10.00
72	32	0	0	0	0	31.35	0.00	0.00
47	39	16	1	0	0	30.70	9.06	5.26
69	28	6	1	1	0	30.68	13.59	0.00
79	22	3	0	0	0	30.63	3.61	0.00
110	43	8	2	0	0	30.44	7.62	7.14
73	29	2	0	0	0	30.25	2.59	0.00
184	62	1	0	0	0	30.16	0.65	0.00
64	30	8	1	1	0	30.04	9.75	5.88
135	99	0	0	0	0	29.48	0.00	0.00
86	18	0	0	0	0	29.31	0.00	0.00
134	58	11	2	1	0	28.65	7.50	0.00
48	8	45	0	0	0	28.07	15.79	66.67
86	17	0	0	0	0	28.07	0.00	0.00
49	56	0	0	0	0	27.12	0.00	0.00
62	24	17	1	0	0	26.41	7.08	45.00
86	17	1	0	0	0	25.86	1.72	0.00
117	37	8	1	0	0	24.46	5.40	0.00
97	31	13	8	0	0	24.38	13.32	64.86
78	24	2	0	0	0	22.69	1.44	0.00
87	16	1	0	0	0	22.59	1.72	0.00
191	56	3	0	0	0	22.49	1.63	0.00
85	16	3	0	0	0	21.21	2.30	0.00
83	19	3	0	0	0	20.85	3.39	0.00
88	14	2	0	0	0	20.27	3.45	0.00
175	72	0	0	0	0	19.40	0.00	0.00
92	11	1	0	0	0	19.31	1.72	0.00
91	13	0	0	0	0	19.12	0.00	0.00
92	12	0	0	0	0	18.97	0.00	0.00
91	13	0	0	0	0	18.97	0.00	0.00
230	59	5	1	0	0	18.80	3.12	0.00
83	16	5	0	0	0	18.39	4.48	0.00
91	13	0	0	0	0	18.26	0.00	0.00
120	25	1	2	1	0	18.05	1.28	0.00
89	13	2	0	0	0	18.03	3.45	0.00
123	24	2	0	0	0	17.91	1.40	50.00
132	30	1	0	0	0	17.61	0.91	0.00
134	25	1	0	0	0	16.54	0.92	0.00
83	21	0	0	0	0	15.91	0.00	0.00
88	16	0	0	0	0	15.73	0.00	0.00

88	15	0	0	0	0	15.39	0.00	0.00
220	60	0	0	0	0	15.28	0.00	0.00
129	20	0	0	0	0	15.10	0.00	0.00
90	12	1	1	0	0	14.58	5.17	0.00
91	8	3	1	0	0	14.51	6.14	16.67
94	10	0	0	0	0	14.37	0.00	0.00
130	15	2	2	0	0	13.71	2.49	0.00
85	17	0	0	0	0	13.69	0.00	0.00
181	42	5	0	0	0	12.82	0.53	0.00
92	10	2	0	0	0	12.70	1.72	0.00
92	10	2	0	0	0	12.67	1.72	0.00
96	5	3	0	0	0	12.23	3.61	33.33
178	37	0	0	0	0	11.80	0.00	0.00
95	7	1	0	0	0	11.23	1.75	0.00
94	7	0	0	0	0	10.53	0.00	0.00
95	9	0	0	0	0	10.50	0.00	0.00
97	6	1	0	0	0	10.34	1.72	0.00
96	8	0	0	0	0	10.34	0.00	0.00
96	8	0	0	0	0	10.34	0.00	0.00
90	14	0	0	0	0	10.14	0.00	0.00
93	11	0	0	0	0	9.40	0.00	0.00
207	39	1	0	0	0	8.93	0.02	0.00
98	6	0	0	0	0	8.62	0.00	0.00
136	13	0	0	0	0	8.55	0.00	0.00
93	11	0	0	0	0	8.54	0.00	0.00
139	9	1	0	0	0	8.41	0.93	0.00
54	2	0	0	0	0	8.33	0.00	0.00
93	49	0	0	0	0	8.33	0.00	0.00
54	1	1	0	0	0	8.33	4.17	100.00
54	2	0	0	0	0	8.33	0.00	0.00
54	2	0	0	0	0	8.33	0.00	0.00
99	5	0	0	0	0	7.76	0.00	0.00
96	8	0	0	0	0	7.74	0.00	0.00
96	6	0	0	0	0	7.47	0.00	0.00
89	15	0	0	0	0	7.24	0.00	0.00
139	9	1	0	0	0	7.17	0.93	0.00
96	6	0	0	0	0	6.41	0.00	0.00
53	3	0	0	0	0	6.25	0.00	0.00
142	7	0	0	0	0	6.07	0.00	0.00
100	4	0	0	0	0	6.03	0.00	0.00
97	7	0	0	0	0	5.64	0.00	0.00
97	6	1	0	0	0	5.64	0.16	0.00
97	7	0	0	0	0	5.33	0.00	0.00
96	7	0	0	0	0	5.26	0.00	0.00
100	3	0	0	0	0	5.26	0.00	0.00
224	23	0	0	0	0	4.96	0.00	0.00
143	5	0	0	0	0	4.72	0.00	0.00
144	5	0	0	0	0	4.67	0.00	0.00
101	3	0	0	0	0	4.31	0.00	0.00
96	6	2	0	0	0	4.23	2.59	100.00
55	1	0	0	0	0	4.17	0.00	0.00
141	8	0	0	0	0	3.80	0.00	0.00
248	32	0	0	0	0	3.55	0.00	0.00
100	4	0	0	0	0	2.19	0.00	0.00
54	2	0	0	0	0	2.08	0.00	0.00
101	3	0	0	0	0	2.04	0.00	0.00
102	2	0	0	0	0	1.88	0.00	0.00

56	0	0	0	0	0	0.00	0.00	0.00
56	0	0	0	0	0	0.00	0.00	0.00
56	0	0	0	0	0	0.00	0.00	0.00
56	0	0	0	0	0	0.00	0.00	0.00
56	0	0	0	0	0	0.00	0.00	0.00
56	0	0	0	0	0	0.00	0.00	0.00
56	0	0	0	0	0	0.00	0.00	0.00
56	0	0	0	0	0	0.00	0.00	0.00
56	0	0	0	0	0	0.00	0.00	0.00
56	0	0	0	0	0	0.00	0.00	0.00
56	0	0	0	0	0	0.00	0.00	0.00
56	0	0	0	0	0	0.00	0.00	0.00
56	0	0	0	0	0	0.00	0.00	0.00
56	0	0	0	0	0	0.00	0.00	0.00
56	0	0	0	0	0	0.00	0.00	0.00
56	0	0	0	0	0	0.00	0.00	0.00
56	0	0	0	0	0	0.00	0.00	0.00
56	0	0	0	0	0	0.00	0.00	0.00
56	0	0	0	0	0	0.00	0.00	0.00
56	0	0	0	0	0	0.00	0.00	0.00
56	0	0	0	0	0	0.00	0.00	0.00
56	0	0	0	0	0	0.00	0.00	0.00
56	0	0	0	0	0	0.00	0.00	0.00
-----	-------	------	----	------	-----	---------------	----------------	-----------------------
